# The Giant Cretaceous Coelacanth (Actinistia, Sarcopterygii) *Megalocoelacanthus dobiei* Schwimmer, Stewart & Williams, 1994, and Its Bearing on Latimerioidei Interrelationships

**DOI:** 10.1371/journal.pone.0049911

**Published:** 2012-11-27

**Authors:** Hugo Dutel, John G. Maisey, David R. Schwimmer, Philippe Janvier, Marc Herbin, Gaël Clément

**Affiliations:** 1 UMR 7207 CNRS-MNHN, Centre de Recherches sur la Paléobiodiversité et les Paléoenvironnements (CR2P), Département Histoire de la Terre, Muséum national d'Histoire naturelle, Paris, France; 2 UMR 7179 CNRS-MNHN, Mécanismes adaptatifs: des Organismes aux Communautés, Département Écologie et Gestion de la Biodiversité, Muséum national d'Histoire naturelle, Paris, France; 3 Division of Paleontology, American Museum of Natural History, City of New York, New York, United States of America; 4 Department of Earth and Space Sciences, Columbus State University, Columbus, Georgia, United States of America; University of Maryland, United States of America

## Abstract

We present a redescription of *Megalocoelacanthus dobiei*, a giant fossil coelacanth from Upper Cretaceous strata of North America. *Megalocoelacanthus* has been previously described on the basis of composite material that consisted of isolated elements. Consequently, many aspects of its anatomy have remained unknown as well as its phylogenetic relationships with other coelacanths. Previous studies have suggested that *Megalocoelacanthus* is closer to *Latimeria* and *Macropoma* than to *Mawsonia*. However, this assumption was based only on the overall similarity of few anatomical features, rather than on a phylogenetic character analysis. A new, and outstandingly preserved specimen from the Niobrara Formation in Kansas allows the detailed description of the skull of *Megalocoelacanthus* and elucidation of its phylogenetic relationships with other coelacanths. Although strongly flattened, the skull and jaws are well preserved and show many derived features that are shared with Latimeriidae such as *Latimeria*, *Macropoma* and *Libys*. Notably, the parietonasal shield is narrow and flanked by very large, continuous vacuities forming the supraorbital sensory line canal. Such an unusual morphology is also known in *Libys*. Some other features of *Megalocoelacanthus*, such as its large size and the absence of teeth are shared with the mawsoniid genera *Mawsonia* and *Axelrodichthys*. Our cladistic analysis supports the sister-group relationship of *Megalocoelacanthus* and *Libys* within Latimeriidae. This topology suggests that toothless, large-sized coelacanths evolved independently in both Latimeriidae and Mawsoniidae during the Mesozoic. Based on previous topologies and on ours, we then review the high-level taxonomy of Latimerioidei and propose new systematic phylogenetic definitions.

## Introduction

*Megalocoelacanthus dobiei* Schwimmer, Stewart & Williams, 1994 is a giant marine coelacanth discovered in 1987 [1] in the Upper Cretaceous Bluffown Formation, southeastern USA. With an estimated length of 3.5 m, *Megalocoelacanthus* is among the largest known coelacanths. Similar dimensions (i.e. more than 2.0 m in total length) are reached by the late Jurassic-mid Cretaceous genus *Mawsonia* from North Africa and South America [2]–[7], and the Early Cretaceous genus *Axelrodichthys* from Brazil [5] also attained a large size. Previous phylogenetic analyses [8]–[14] supported a sister-group relationship of *Mawsonia* and *Axelrodichthys* within Mawsoniidae Schultze, 1993 [15]. This close affinity between toothless, large-sized Mesozoic coelacanths raised the question whether these features could be synapomorphies of a putative clade or have evolved independently in several lineages. The evolution of large sized, toothless coelacanths has been briefly discussed by Schwimmer *et* *al.* [1] who suggested that these features evolved independently in *Mawsonia* and *Megalocoelacanthus*. The authors have suggested that *Megalocoelacanthus* is closer to *Macropoma* and *Latimeria* than to *Mawsonia*. However, their assumption was only based on the comparison of meristic data of some anatomical features (table 2 in [1]), but not on a phylogenetic analysis.

Since its discovery, the relationships of *Megalocoelacanthus* with other coelacanths have never been investigated. Here we present a description of cranial and postcranial skeleton of *Megalocoelacanthus* based on new and holotype material. A phylogenetic analysis of 39 taxa and 110 characters is performed to clarify the position of *Megalocoelacanthus* among coelacanths, and its bearings on Mesozoic coelacanth's interrelationships are subsequently discussed. Implications for the coelacanth taxonomy will also be discussed and the application of phylogenetic definitions to coelacanth taxonomy will be proposed based on our novel topology.

## Materials and Methods

### 1. Geological context

**Figure 1:**
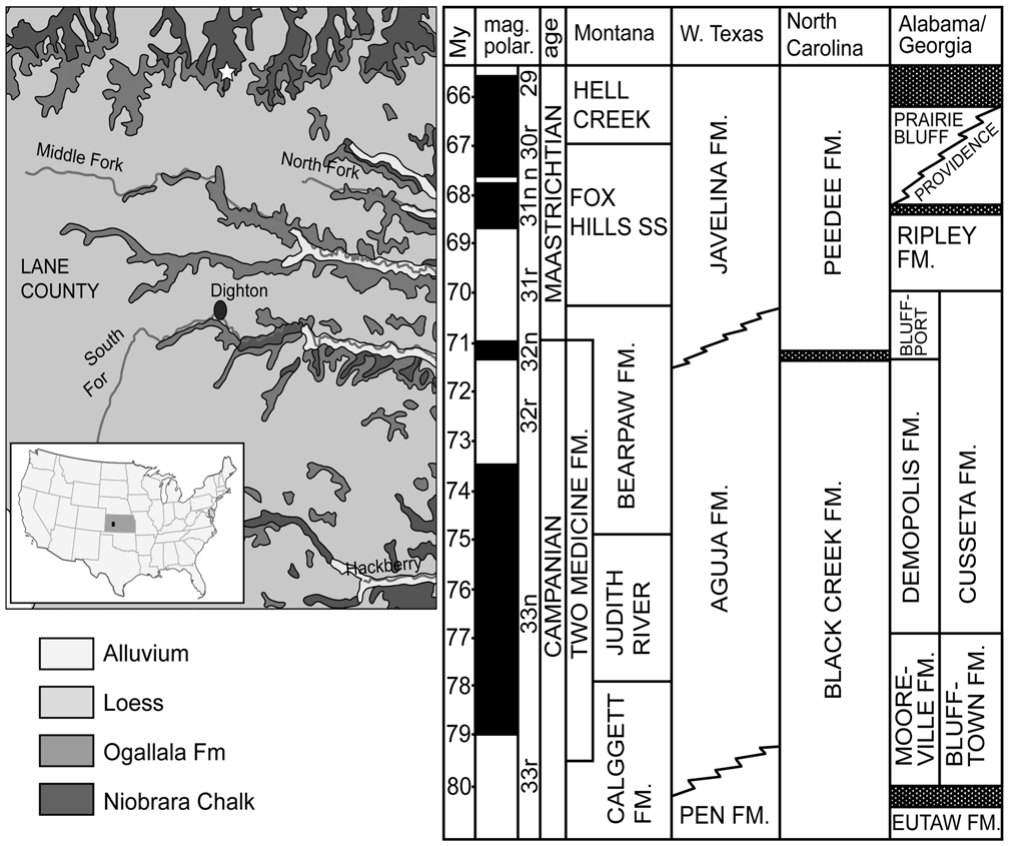
Geological context of *Megalocoelacanthus dobiei* Schwimmer, Stewart & Williams, 1994. **Left,** white star indicates the geographic location of the locality of AMNH FF 20267 in the Niobrara chalk of Lane County Kansas, USA (modified from http://www.kgs.ku.edu/General/Geology/County/klm/lane.html). **Right,** stratigraphic correlation chart between the principal stratigraphic units from North America (taken from [1]). The Niobrara Formation in Lane County, Kansas, is correlated with the Blufftown, Mooreville, and Eutaw Formations in Alabama and Georgia, where the first occurrences of *Megalocoelacanthus* were reported.

*Megalocoelacanthus* remains are known only from the United States. The holotype CCK 88-2-1 consists of cranial and branchials elements [1], and was found in the lower part of the Blufftown Formation in eastern Alabama, that is early Campanian in age (Figure 1). Remains of *Megalocoelacanthus* were also first reported in five additional regional localities from the southeastern states of Alabama and Georgia [1], along with a single coronoid fragment from New Jersey. Subsequently, unpublished specimens have been collected in coeval strata in Mississippi (Schwimmer, Earl Manning, pers. comm.), and Kansas. All known fossils of *Megalocoelacanthus* with well-known stratigraphic associations are of late Santonian to mid-Campanian age [1], except the New Jersey specimen which is in a late Campanian-early Maastrichtian deposit. However, the latter fossil is a highly ablated principal coronoid fragment, preserved in a near shore lag deposit, which may have been reworked from older material: its age is thus uncertain.

### 2. Material

This redescription of *Megalocoelacanthus* is mainly based on AMNH FF 20267, which was collected in 2007 in the Niobrara Formation, in the Northern Lane County, Kansas (Figure 1). It is early Campanian in age and thus approximately coeval with the holotype CCK 88-2-1. The new specimen consists of skull (both ethmosphenoid and otoccipital portions), snout, lower jaws, gular plates, branchial arches, urohyal, hyoid skeleton, and shoulder girdle. Although most of these isolated remains are strongly flattened laterally, they are outstandingly preserved. Significant elements from the holotype specimen CCK 88-2-1 are also included in the description for further comments.

### 3. Nomenclatural Acts

The electronic version of this document does not represent a published work according to the International Code of Zoological Nomenclature (ICZN), and hence the nomenclatural acts contained in the electronic version are not available under that Code from the electronic edition. Therefore, a separate edition of this document was produced by a method that assures numerous identical and durable copies, and those copies were simultaneously obtainable (from the publication date noted on the first page of this article) for the purpose of providing a public and permanent scientific record, in accordance with Article 8.1 of the Code. The separate print-only edition is available on request from PLoS by sending a request to *PLoS ONE*, Public Library of Science, 1160 Battery Street, Suite 100, San Francisco, CA 94111, USA along with a check for $10 (to cover printing and postage) payable to “Public Library of Science”.

In addition, this published work and the nomenclatural acts it contains have been registered in ZooBank, the proposed online registration system for the ICZN. The ZooBank LSIDs (Life Science Identifiers) can be resolved and the associated information viewed through any standard web browser by appending the LSID to the prefix “http://zoobank.org/”. The LSID for this publication is: urn:lsid:zoobank.org:pub:6F452EF9-02A6-4B3F-9E0B-3E4C14BADC80.

## Results

### 1. Anatomical description

#### 1.1 Dermal bones of the skull roof

**Figure 2:**
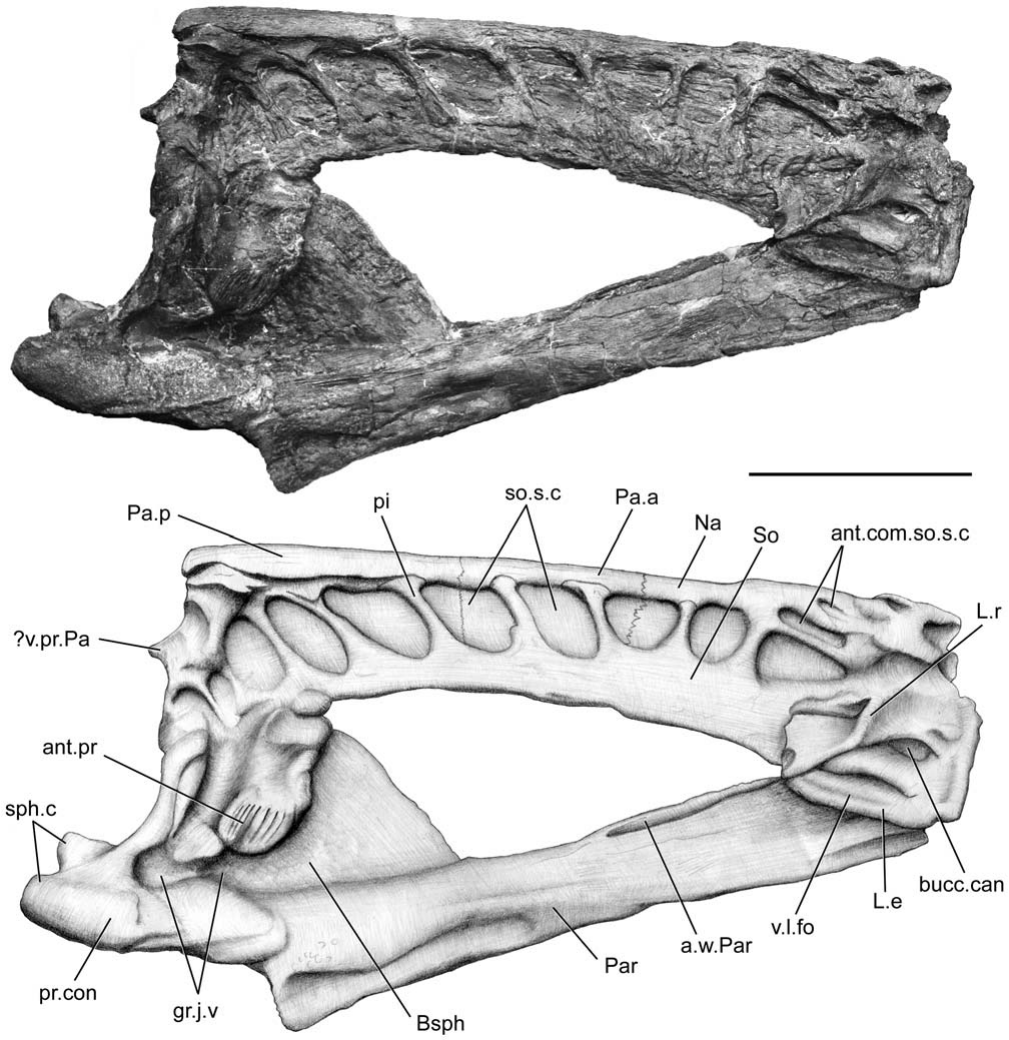
*Megalocoelacanthus dobiei* Schwimmer, Stewart & Williams, 1994, AMNH FF 20267 from lower Campanian of the Niobrara Formation. Ethmosphenoid portion of the skull in right lateral view. Abbreviations: **ant.com.so.s.c,** anterior commissure of supraorbital sensory line canal; **ant.pr,** antotic process; **a.w.Par,** ascending wing of parasphenoid; **Bsph,** basisphenoid; **bucc.can,** buccal canal; **gr.j.v,** groove for jugular vein; **L.e,** lateral ethmoid; **L.r,** lateral rostral; **Na,** nasal; **Pa.a,** anterior parietal; **Pa.p,** posterior parietal; **Par,** parasphenoid; **pi,** pillar; **pr.con,** processus connectens; **sph.c,** sphenoid condyle; **So,** supraorbital series; **so.s.c,** supraorbital sensory line canal; **v.l.fo,** ventrolateral fossa; **?v.pr.Pa,** ventral (descending) process of the parietal. Scale bar  = 10 cm.

**Figure 3:**
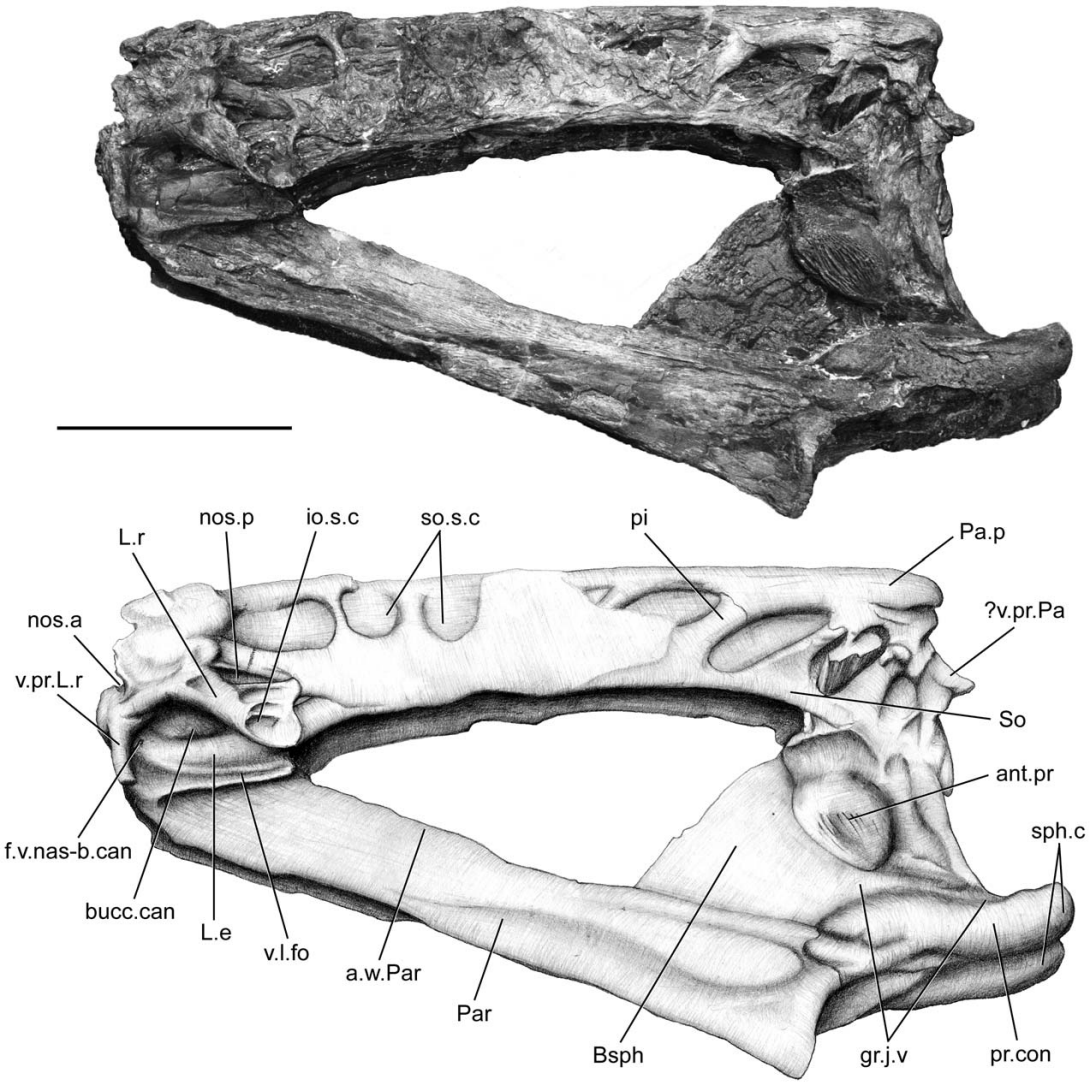
*Megalocoelacanthus dobiei* Schwimmer, Stewart & Williams, 1994, AMNH FF 20267 from lower Campanian of the Niobrara Formation. Ethmosphenoid portion of the skull in left lateral view. Abbreviations: **ant.pr,** antotic process; **a.w.Par,** ascending wing of parasphenoid; **Bsph,** basisphenoid; **bucc.can,** buccal canal; **f.v.nas-b.can,** foramen for ventral branch of naso-basal canal; **gr.j.v,** groove for jugular vein; **io.s.c,** infraorbital sensory line canal; **L.e,** lateral ethmoid; **L.r,** lateral rostral; **Na,** nasal; **nos.a,** anterior nostril; **nos.p,** posterior nostril; **Pa.p,** posterior parietal; **Par,** parasphenoid; **pi,** pillar; **pr.con,** processus connectens; **sph.c,** sphenoid condyle; **So,** supraorbital series; **so.s.c,** supraorbital sensory line canal; **v.l.fo,** ventrolateral fossa; **v.pr.L.r,** ventral (descending) process of the lateral rostral; **?v.pr.Pa,** ventral (descending) process of the parietal. Scale bar  = 10 cm.

**Figure 4:**
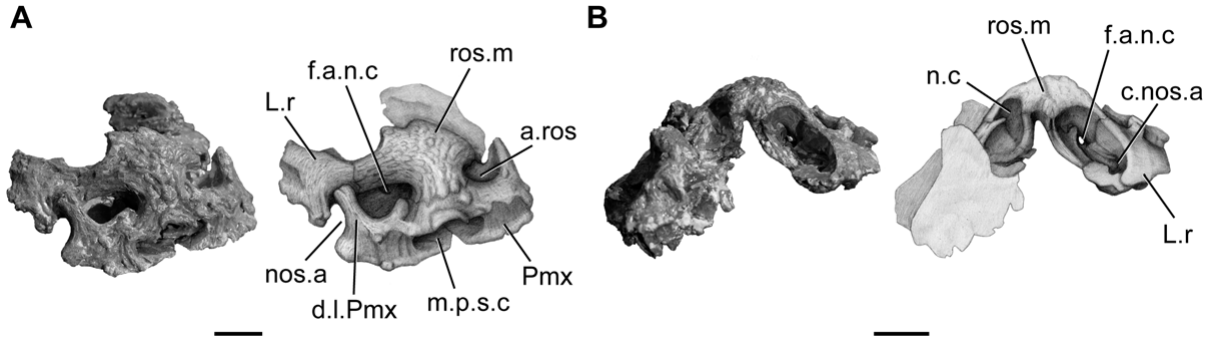
*Megalocoelacanthus dobiei* Schwimmer, Stewart & Williams, 1994, AMNH FF 20267 from lower Campanian of the Niobrara Formation. Isolated snout. **A,** right anterolateral view; **B,** posterior view. Abbreviations: **ant.ros,** anterior opening for the rostral organ; **c.nos.a,** canal for the anterior nostril; **d.l.Pmx,** dorsal lamina of the premaxilla; **f.a.n.c,** anterior foramen of the nasal capsule; **L.r,** lateral rostral; **m.p.s.c,** median pore for the sensory line canal; **n.c,** nasal capsule; **nos.a,** anterior nostril; **Pmx,** premaxilla. Scale bar  = 1 cm.

**Figure 5:**
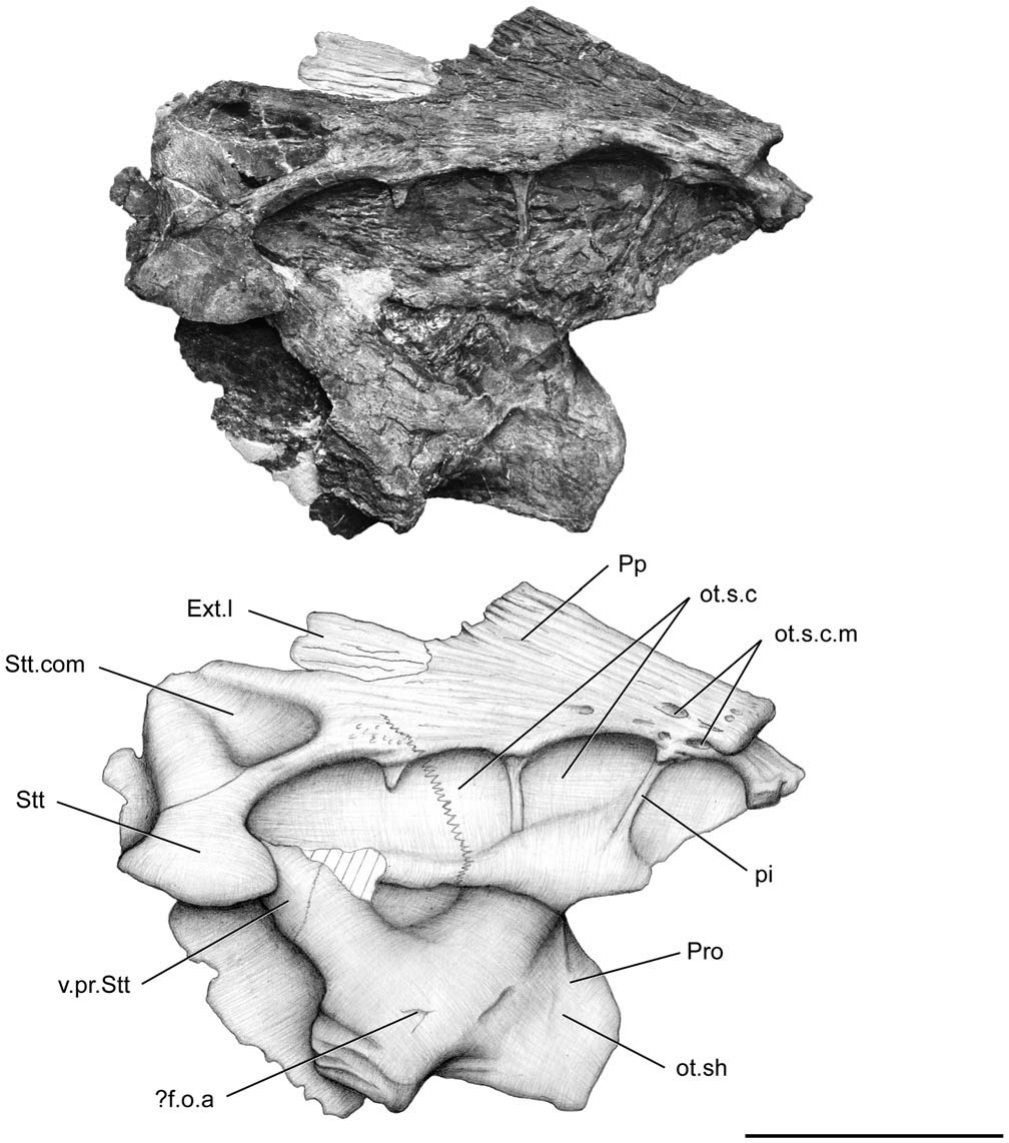
*Megalocoelacanthus dobiei* Schwimmer, Stewart & Williams, 1994, AMNH FF 20267 from lower Campanian of the Niobrara Formation. Otoccipital portion of the skull in right lateral view. Abbreviations: **Ext.l,** lateral extrascapular; **?f.o.a,** foramen for the orbitonasal artery; **ot.s.c,** otic sensory line canal; **ot.s.c.m,** medial branch of the otic sensory line canal; **ot.sh,** otic shelf; **pi,** pilar; **Pp,** postparietal; **Pro,** prootic; **Stt,** supratemporal; **Stt.com,** supratemporal commissure; **v.pr.Stt,** ventral (descending) process of the supratemporal. Scale bar  = 10 cm.

**Figure 6:**
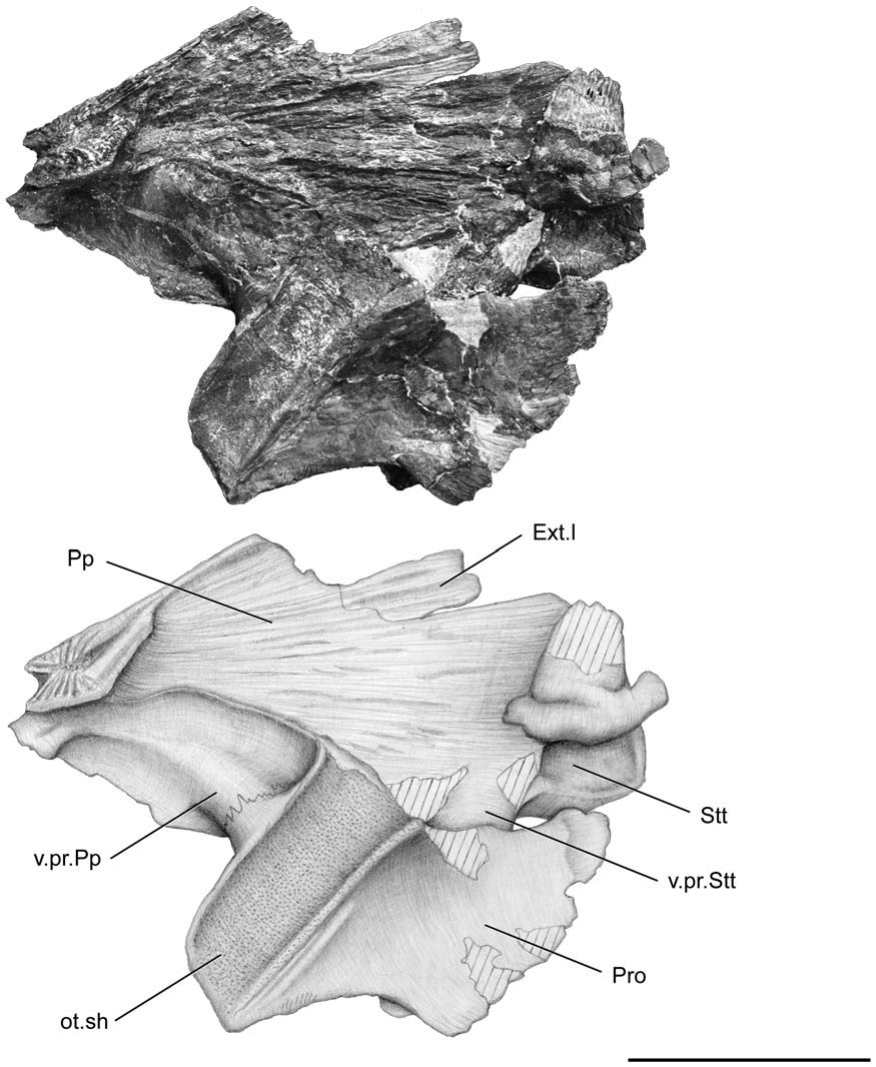
*Megalocoelacanthus dobiei* Schwimmer, Stewart & Williams, 1994, AMNH FF 20267 from lower Campanian of the Niobrara Formation. Otoccipital portion of the skull in right medial view. Abbreviations: **Ext.l,** lateral extrascapular; **ot.sh,** otic shelf; **Pp,** postparietal; **Pro,** prootic; **Stt,** supratemporal; **v.pr.Pp,** ventral (descending) process of the postparietal; **v.pr.Stt,** ventral (descending) process of the supratemporal. Scale bar  = 10 cm.

**Figure 7:**
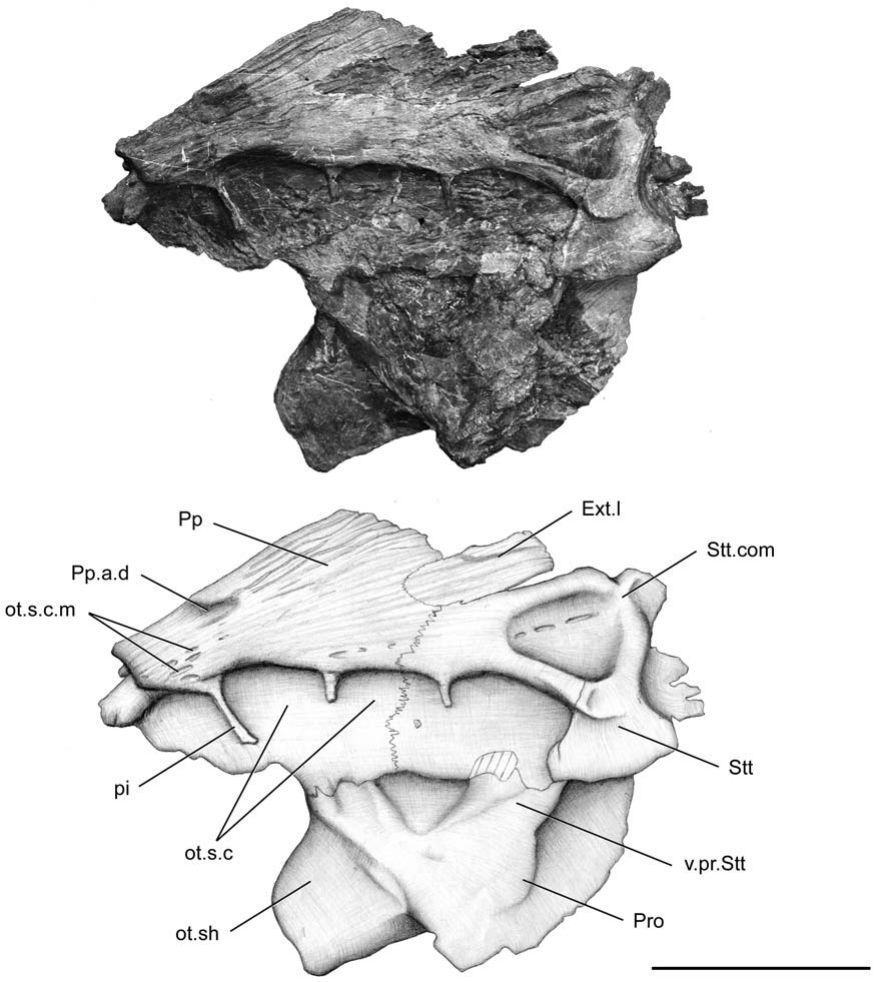
*Megalocoelacanthus dobiei* Schwimmer, Stewart & Williams, 1994, AMNH FF 20267 from lower Campanian of the Niobrara Formation. Otoccipital portion of the skull in left lateral view. Abbreviations: **Ext.l,** lateral extrascapular; **ot.s.c,** otic sensory line canal; **ot.s.c.m,** medial branch of the otic sensory line canal; **ot.sh,** otic shelf; **pi,** pillar; **Pp,** postparietal; **Pp.a.d,** anterior depression of the postparietal; **Pro,** prootic; **Stt,** supratemporal; **Stt.com,** supratemporal commissure; **v.pr.Stt,** ventral (descending) process of the supratemporal. Scale bar  = 10 cm.

**Figure 8:**
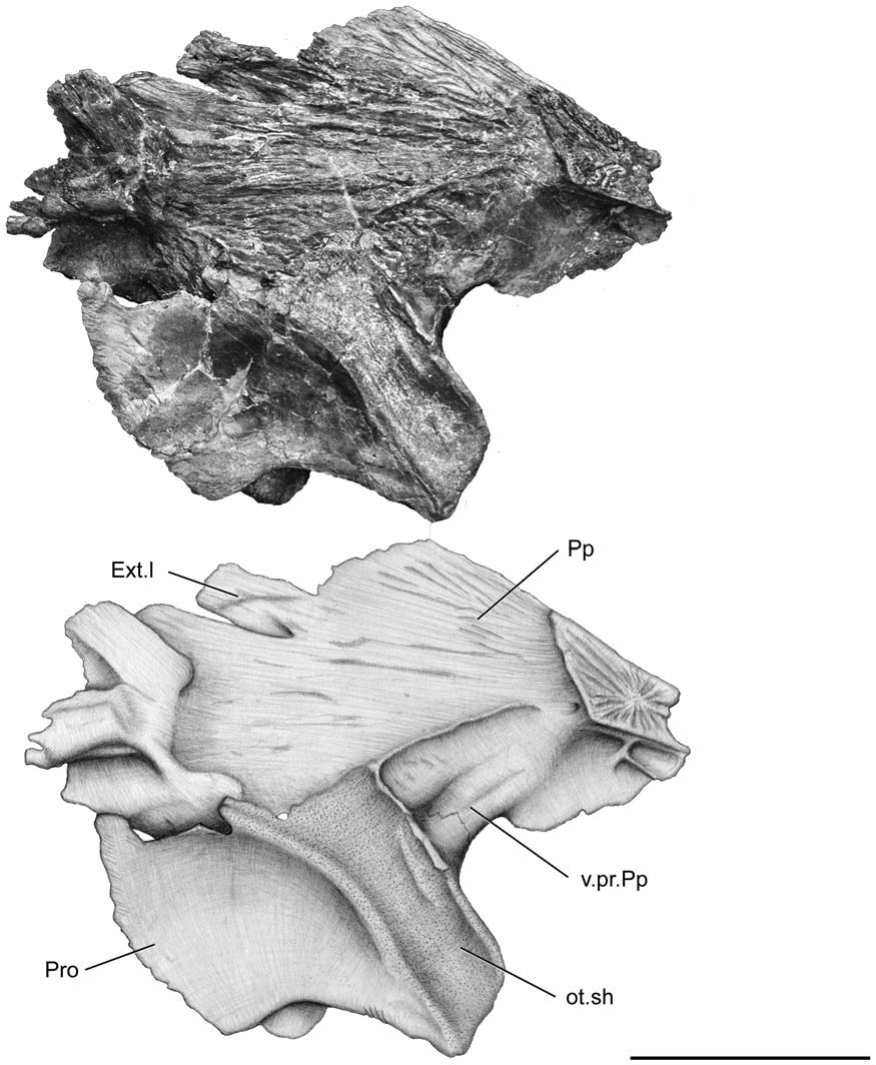
*Megalocoelacanthus dobiei* Schwimmer, Stewart & Williams, 1994, AMNH FF 20267 from lower Campanian of the Niobrara Formation. Otoccipital portion of the skull in left medial view. Abbreviations: **Ext.l,** lateral extrascapular; **ot.sh,** otic shelf; **Pp,** postparietal; **Pro,** prootic; **v.pr.Pp,** ventral (descending) process of the postparietal. Scale bar  = 10 cm.

Despite strong lateral compression, all elements are very well preserved and allow detailed description. The parietonasal shield (Figures 2, 3, 4) is longer than the postparietal shield (Figures 5, 6, 7, 8) very narrow and straight in lateral view. The intracranial joint is transversely straight. The parietonasal series consists of two pairs of parietals (Pa.a, Pa.p, Figures 2, 3), but the number of nasals cannot be assessed, and only the posteriormost nasal (Na, Figure 2) can be observed. However, its relationships with the elements of the anteriormost part of the ethmosphenoid portion of the skull are very difficult to observe. The bones of the parietonasal series are slender and elongated. The posterior parietals are the largest of the series as in *Macropoma*, *Holophagus* [12] and *Swenzia* [8], [16], and their center of ossification is situated posteriorly. The posterior parietals meet the anterior parietals in a transverse indented suture. The median suture between the two paired parietals is straight and only leaves a narrow gap on the midline, probably due to the settling of the bones under lateral constraints during fossilization. The sutures between the different parietonasal elements (posterior parietal/anterior parietal; anterior parietal/nasal) extend far laterally, and extend into the vacuity of the supraorbital sensory line canal at the level of the contact between the dermal bones (Figure 2). The supraorbital series can be distinguished by the presence of sutures between to the parietonasal series and the pillars that are crossing the supraorbital sensory line canal (So, pi, so.s.c, Figures 2, 3). However their precise number cannot be assessed due to the poor preservation of this area of the skull roof. On both side of the skull, an elbowed process (?v.pr.Pa, Figures 2, 3) extends just below the margin of joint between the posterior pair of parietal and the postparietal. This process could correspond to the pedicel of the ventral process of the parietal, which forms a ridge that slides in the groove on the postparietal.

The lateral rostral (L.r, Figures 2, 3) is a large bone which extends posteroventrally as a relatively short and flattened tube that encloses the infraorbital sensory line canal. There is no evidence of grooves on its dorsal surface, unlike in *Diplurus*, *Macropoma*, *Latimeria*, *Laugia* [12], *Swenzia* [8], and *Rhabdoderma* [17]. The lateral rostral usually separates the anterior nostril from the posterior nostril. Anteriorly, the ventral process of the lateral rostral (v.pr.L.r, Figures 3) is sutured to the lateral ethmoid, and its anterior margin is notched and corresponds to the posterior margin of the opening for the anterior nostril (nos.a, Figure 3) as in *Macropoma* (figure 6.10 in [12]). On both sides, the posterior margin of the lateral rostral is notched dorsally to the tube enclosing the infraorbital sensory line canal. A space is clearly observable on the left side, between the lateral rostral and the supraorbital series and suggests that the position of the opening of the posterior nostril (nos.p, Figure 3) was similar to that in other coelacanths. On the left side, a conspicuous suture is observable between the lateral rostral and the supraorbital series.

The tip of the snout is preserved in three dimensions, but is isolated from the rest of the skull (Figure 4). The snout is partially fused and consists of a pair of premaxilla, a median rostral, and the right anterior portion of the lateral rostral (Pmx, ros.m, L.r, Figure 4A). It shows no trace of strong distortion: its shape may thus reflect the actual width of the skull roof as in *Whiteia* [12] and *Macropoma* (figures 3.15, 3.19A in [12]). The tip of the snout appears to be strongly consolidated due to the tight suture between the premaxilla and the median rostral (Figure 4A). Although the snout is heavily ossified, it may have been loosely attached to the lateral ethmoid, anterior nasal, and tectal (if present). This condition is also observed in *Macropoma lewesiensis* (BMNH 4207). The entire surface of the snout is ornamented with coarse rugosities, making it difficult to observe the suture between the bones precisely. The premaxilla is robust, bears no teeth, and is separated at the symphysis from its antimere by a large trapezoidal, median pore of the sensory line canal (m.p.s.c, Figure 4A). Such a condition is also observed in *Macropoma* (figure 3.20B in [12]) and *Latimeria* (figure 8 p.s.m.e.m in [18]), but the median pore of the sensory line canal is much larger in *Megalocoelacanthus*. The ventral half of the premaxilla is very broad and the base of the bone extends medially as a thin flattened surface that closes ventrally the median pore of the sensory line canal (Figure 4A). The base of the premaxilla meets its antimere in a recess, posterior to the opening of the median pore of the sensory line canal. The anteroventral margin of the premaxilla is slightly folded ventrally, and crenate. Ventrally, the right premaxilla presents a raised area pierced by a pore that is directed posteroventrally. The dorsal lamina of the premaxilla (d.l.Pmx, Figure 4A) is well expanded and forms the lateral margin of the opening of the anterior tube of the rostral organ (a.ros, Figure 4A).

The median rostral (ros.m, Figure 4A, 4B), usually poorly preserved in coelacanths, is here complete and in its natural position. It is cross-shaped, shorter than broad, and shows no indication of strong distortion. It is raised anteroposteriorly, and its surface is ornamented with coarse rugosities. On both side, it shows a curved, posteroventral extension towards the dorsal lamina of the premaxilla. The better-preserved right side of the snout allows the description of the relations between these bones (Figure 4A). The median rostral reaches the lateral tectal medial to the premaxilla, and forms the dorsal margin of the opening of the anterior tube of the rostral organ. Posteriorly, the median rostral caps the anterior wall of a large and deep ovoid cavity (n.c, Figure 4B). The lateral wall, formed anteriorly by the lateral extension of the median rostral, is then extended by the lateral rostral that curves medially in its posterior portion. The ovoid cavity is paired, but not separated from its antimere by a medial septum. Indeed, it is clearly individualized on either side of the snout by a medial, bell-shaped cavity (Figure 4B). It is unlikely that this paired cavity corresponds to the rostral organ cavity of *Latimeria*, because the latter is median, and usually situated more posteriorly along the body axis, posterodorsally to the nasal capsule. Consequently, the anterior portion of the rostral organ lies dorsal to the olfactory capsules and the internasal septum. The very anterior position, the individualization of the lateral cavities, and their openings towards the exterior, suggests that they housed the nasal capsules. As in *Latimeria*, the cavities are oriented anteromedially. The right nasal cavity is opened by a canal directed laterally, that pierces the lateral rostral (c.nos.a, Figure 4B) and opens ventrally to the notch formed by the contact of the dorsal lamina of the premaxilla and the lateral rostral. This opening is interpreted as being the anterior nostril (nos.a, Figure 4A). Another foramen pierces the anterior wall of the nasal capsule towards the opening of the anterior tube of the rostral organ (f.a.n.c, Figure 4A, 4B). The medial cavity that separates the nasal capsules is partly closed ventrally by the flattened base of the premaxillaries, and opens anteriorly through the median pore of the sensory line canal.

The postparietal shield of the skull is divided medially into two strongly flattened halves (Figures 5, 6, 7, 8). Although it is slightly broken posteriorly and medially, the postparietal shield is shorter than the parietonasal shield. The anterior tip of the postparietal is thick and narrow, leaving no gap between the two halves when they are put in contact. In its anteriormost portion, the postparietal shield is thus as broad as the parietonasal shield. It is most probable that the postparietal shield was strongly vaulted and broad over most of the otoccipital portion as in *Macropoma*, *Libys*, *Holophagus* [12] and *Swenzia* [8], and narrow anteriorly. The center of ossification of the postparietal is situated very close to the joint margin (Figures 6, 8), as in *Libys* [12]. The anterior thickening of the postparietal bears a concave facet (Figure 8), which probably matched the contour of the parietal descending process. The ventral surface of the anterior thickening of the postparietal exhibits a ridge that is oriented anteroposteriorly and flanked by tiny pores. The dorsal surface of the postparietal (Pp, Figures 5, 7) shows well-marked longitudinal grooves, especially in its posterior portion. Similar grooves are also present in *Macropoma*, *Latimeria* [12] and *Swenzia* [8], and were interpreted as the anterior branches of the supratemporal sensory line canal commissure. However, the same interpretation is difficult to make because of the poor preservation of the surface of the skull roof. The anterior portion of the postparietal presents a semi-circular depression (Pp.a.d, Figure 7) as in *Swenzia* [8]. The suture between the postparietals and supratemporals runs anteroventrally on both sides (Figures 5, 7). It extends far ventrolaterally through the third opening of the sensory line canal and terminates at the level of its ventral margin: such a condition is also observable in *Libys* (figure 3.17 in [12]). The supratemporal (Stt, Figures 5, 6, 7) is relatively large and represents almost half of the surface of the postparietal shield. On the left side of the skull, the lateral extrascapular (Ext.l, Figures 5, 6, 7, 8) is present, dorsal to the suture between the postparietal and the supratemporal.

#### 1.2 Sensory line canals

The otic sensory line canal opens through remarkably large vacuities along the supraorbital series and flanks the parietonasal series laterally (so.s.c, Figures 2, 3). The ethmosphenoid portion of the skull bears nine vacuities and the otoccipital portion five. Slender pillars (pi, Figures 2, 3, 5, 7) separate adjacent vacuities. Several pillars display a clear separation between their dorsal edge and the parietonasal shield. This could be attributed to the lateral compression, or it could suggest that most of the pillars may have been sutured to the parietonasal shield. We support the latter assumption based on the presence of clear suture between the anteriormost pillar and the lateral rostral on both side of the skull. Consequently, we identify the pillars as expansions of the supraorbital series. The condition observed in *Megalocoelacanthus* is thus very similar to that of the Jurassic genus *Libys*, where the pillars forming the large vacuity of the supraorbital sensory line canal are interpreted as elements of the supraorbital series (Dutel pers. obs. on BMNH P.3337). A twist in the orientation of the pillars is observable along the antero-posterior axis (Figures 2, 3). The first three pillars are oriented anterodorsally whereas the more posterior ones are oriented posterodorsally. The supratemporal bears one pillar, the posterior parietal four, the anterior parietal two, and the anteriormost ones are borne by the nasals. A vacuity occurs between the edges of adjacent bones of the parietonasal series (i.e. posterior parietal/anterior parietal; anterior parietal/nasal). The suture between the bones of the parietonasal series extends through the vacuity and is subsequently overlapped by the ventral edge of the cavity (Figure 2). This suggests that the parietonasal series could have extended laterally on both sides of the skull, so that it was overlapped by the supraorbital series. The first three vacuities are more dorsal in position than the posterior one. Their shapes are also different along the antero-posterior axis (Figure 2): the anteriormost vacuities are dorsoventrally flattened while the posteriormost are anteroposteriorly flattened. This suggests that on either side, the branches of the sensory line canal were oriented anteromedially along the ethmosphenoid portion of the skull, meeting dorsomedially behind the lateral rostral to form the antorbital sensory line canal commissure (ant.com.so.s.c, Figure 2). The position of the antorbital commissure is thus at the same level along the shield as in *Latimeria* [19] and *Macropoma* (figure 3.18 in [12]).

The course of the sensory line canal along the anteriormost portion of the skull is more difficult to reconstruct. The lateral rostral (L.r, Figures 2, 3) shows no pores on its dorsal surface, as in *Diplurus*, *Macropoma*, *Latimeria*, *Laugia* [12], *Swenzia* [8] and *Rhabdoderma* [17]. Consequently, the course of the infraorbital sensory line canal cannot be followed throughout the surface of the bone. *Megalocoelacanthus* presents, as in *Macropoma*, *Rhabdoderma*, and *Whiteia*, a small median opening between the premaxillae that was interpreted as the median pore of the sensory line canal (m.p.s.c, Figure 4A). Forey [12] suggested that this opening was related to the ethmoid commissure. Following the interpretation of Forey [12], the presence of such a pore in *Megalocoelacanthus* could suggest the presence of an ethmoid commissure running beneath the bones, as in *Latimeria* [19] and *Macropoma* [12], and emitting canals that are directed towards the tip of the snout as in *Latimeria.*

In the otoccipital portion, the sensory line canal passes within the postparietal and the supratemporal (Figures 5, 7). The vacuities present on the otoccipital portion of the skull are larger than those of the ethmosphenoid portion and nearly square. The posteriormost vacuity of the otoccipital portion opens throughout the supratemporal. It is triangular and that of the left side is pierced by two foramina that are aligned along the anteroposterior axis (Figure 7). A slight swelling on the dorsal surface suggests that the otic sensory line canal, lateral sensory line canal and supratemporal commissure (Stt.com, Figures 5, 7) met near the posterior edge of the supratemporal, as in *Libys*. A few pits lie on the dorsal surface of the postparietal (ot.s.c.m, Figures 5, 7), close to the joint margin. As in *Holophagus* (figure 3.18 in [12]), *Libys* (figure 3.17 in [12]), *Macropoma* (figure 3.21 in [12]) these pits could be related to the medial branch of the otic sensory line canal. Pit lines are not observed on the postparietal.

#### 1.3 Neurocranium

**Figure 9:**
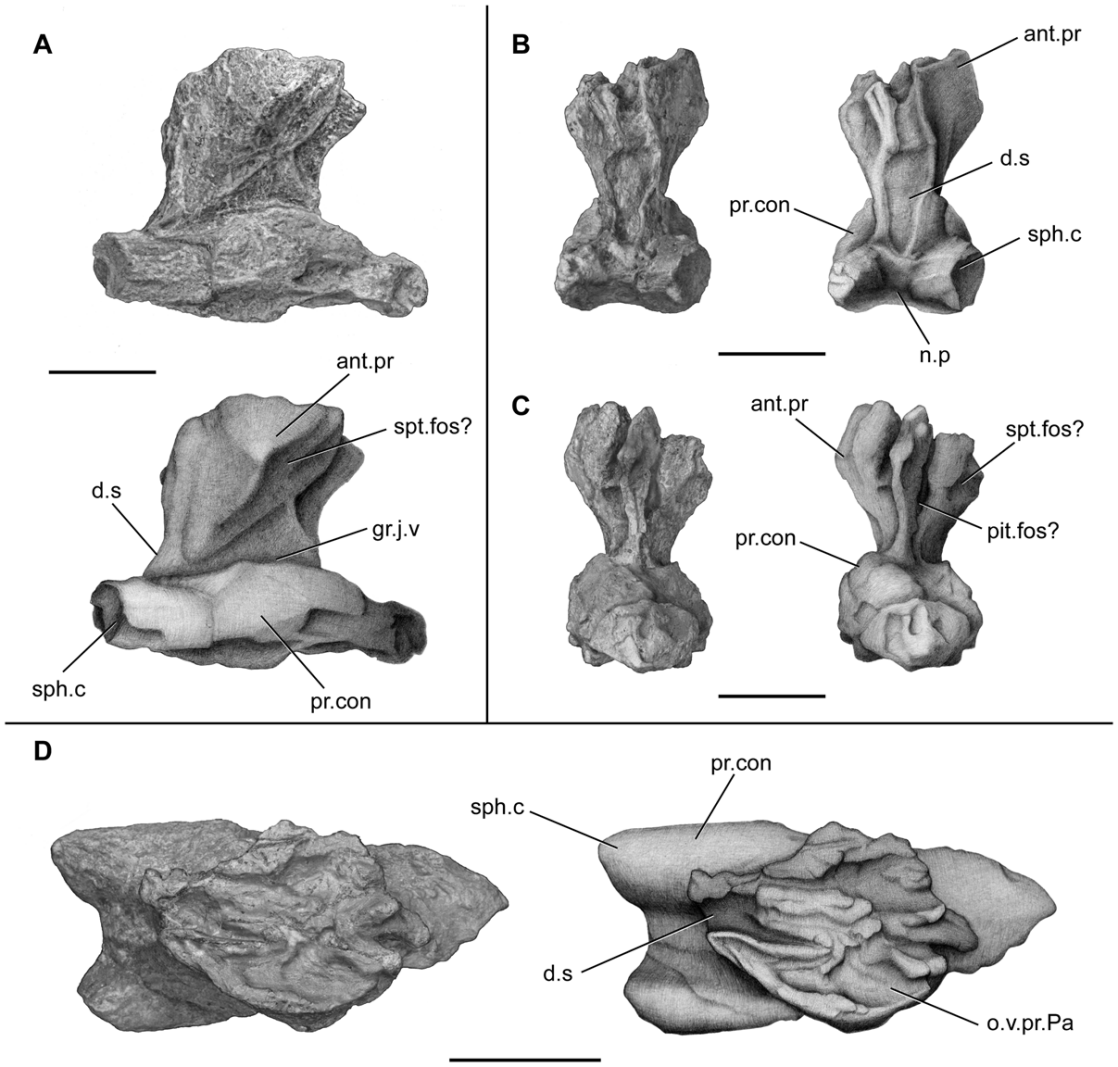
*Megalocoelacanthus dobiei* Schwimmer, Stewart & Williams, 1994, holotype specimen CCK 88-2-1 from lower Campanian of the Blufftown Formation. Isolated basisphenoid. **A,** right lateral view; **B,** posterior view; **C,** anterior view; **D,** dorsal view. Abbreviations: **ant.pr,** antotic process; **d.s,** dorsum sellae; **gr.j.v,** groove for jugular vein; **n.p,** notochordal pit; **o.v.pr.Pa,** overlapping surface for descending process of parietal; **pit.fos?,** pituitary fossa; **pr.con,** processus connectens; **sph.c,** sphenoid condyle; **spt.fos?,** suprapterygoid fossa. Scale bar  = 5 cm.

**Figure 10:**
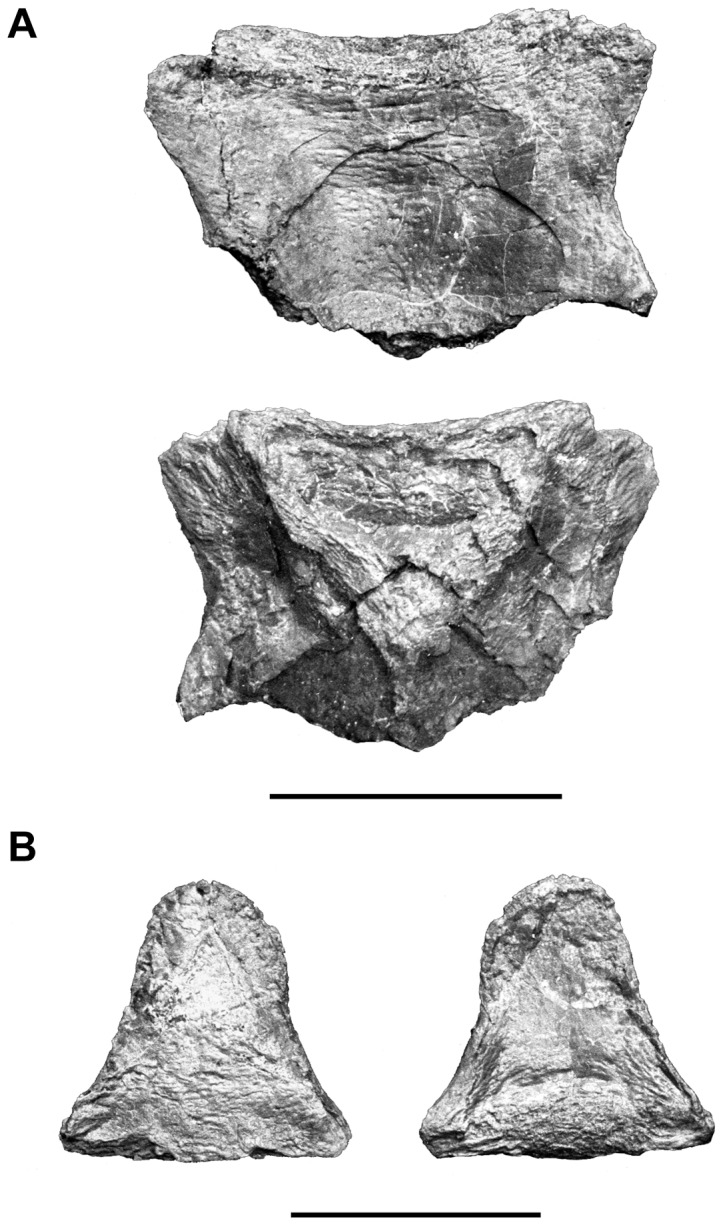
*Megalocoelacanthus dobiei* Schwimmer, Stewart & Williams, 1994, AMNH FF 20267 from lower Campanian of the Niobrara Formation. **A,** anterior catazygal. **B,** posterior catazygal.

The neurocranium of *Megalocoelacanthus* is extremely well preserved although strongly flattened laterally. It is extensively ossified, and completely divided into ethmosphenoid (Figures 2, 3, 4) and otoccipital portions (Figures 5, 6, 7, 8), which are articulated through a transversally straight intracranial joint. The basisphenoid of the holotype specimen CCK 88-2-1 (Figure 9) is isolated and well preserved. Anterior and posterior catazygals are preserved in AMNH FF 20267 (Figure 10).

The ethmosphenoid portion (Figures 2, 3) is robust, longer than the otoccipital portion, but its anterior end is broken at the level of the anterior margin of the lateral rostral. It consists of a large basisphenoid tightly sutured to the parasphenoid, and a pair of lateral ethmoids. The anterior face of the lateral ethmoid (L.e, Figures 2, 3) is roughened and sutured to the ventral process of the lateral rostral. Posterior to this area the lateral ethmoid is notched to form the ventral margin of the buccal canal (bucc.can, Figures 2, 3). The lateral ethmoid enlarges dorsally towards its posterior end, which is partially overlapped by the lateral rostral. A large foramen (f.v.nas-b.can, Figure 3), interpreted as the point of emergence of the ventral branch of the naso-basal canal, pierces the lateral ethmoid beneath the contact with the ventral process of the lateral rostral. Such a foramen is also described in *Undina* and *Macropoma* [12]. The shallow ventrolateral fossa (v.l.fo, Figures 2, 3) is clearly marked by an oblique, dorsal depression.

**Figure 11:**
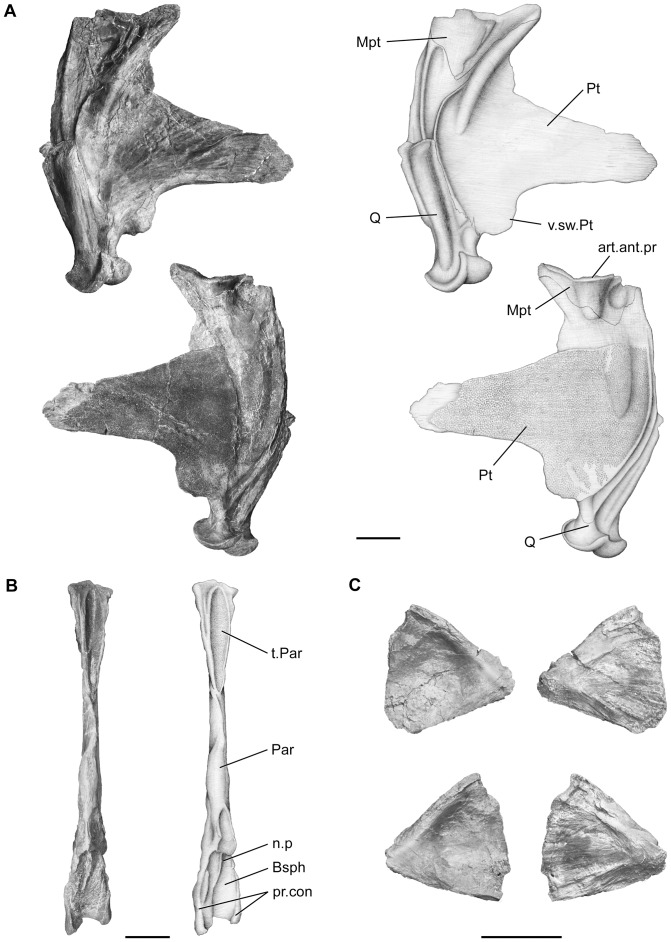
*Megalocoelacanthus dobiei* Schwimmer, Stewart & Williams, 1994, AMNH FF 20267 from lower Campanian of the Niobrara Formation. Palate bones. **A,** right palatoquadrate in lateral (top) and medial (bottom) views. **B,** parasphenoid in ventral view. **C,** right (top) and left (bottom) autopalatines in lateral (left) and medial (right) views. Abbreviations: **art.ant.pr,** surface for articulation with the antotic process; **Bsph,** basisphenoid; **Mpt,** metapterygoid; **n.p,** notochordal pit; **Par,** parasphenoid; **pr.con,** processus connectens; **Pt,** pterygoid; **Q,** quadrate; **t.Par,** toothed area of parasphenoid; **v.sw.Pt,** ventral swelling of pterygoid. Scale bar  = 5 cm.

The basiphenoid is preserved in connection with the whole ethmosphenoid portion of the skull in AMNH FF 20267 (Figures 2, 3), and as an isolated element in the holotype specimen CCK 88-2-1 (Figure 9). In AMNH FF 20267, the basisphenoid is relatively large compared to the entire ethmosphenoid portion of the skull. The paired processus connectens (pr.con, Figures 2, 3, 9) are robust, well developed, and posteriorly elongated. The angle between the parasphenoid and the processus connectens is smaller than what can be observed in *Latimeria* and *Macropoma* (Figures 2, 3). The surface of the processus connectens is rugose and was probably capped by cartilage to articulate with the otic shelf of the prootic. The area between the processus connectens and the antotic process is marked by a deep groove (gr.j.v, Figures 2, 3, 9A) that houses the jugular vein in *Latimeria* [18]. The antotic process (ant.pr, Figures 2, 3, 9A, 9B, 9C) is prominent, oriented anteroventrally, and covered with strong ridges in AMNH FF 20267 on which may have been anchored the adductor palatini muscle. In AMNH FF 20267 and CCK 88-2-1 the anterodorsal part of the antotic process is notched and marks the posterior margin of the suprapterygoid fossa (spt.fos, Figures 2, 3, 9B, 9C). No trace of foramina can be observed on both specimens, probably due to the artefact of the strong lateral compression of the bones. Although both basisphenoids are flattened laterally, the one of the holotype specimen CCCK 88-2-1 (Figure 9) is less so and enables further comparisons with other coelacanths. The basisphenoid of *Megalocoelacanthus* is higher and narrower than that of *Axelrodichthys*, *Mawsonia* and *Diplurus* [5], [20]. The dorsum sella (d.s, Figure 9) is much elongated in *Megalocoelacanthus* compared with these genera. As in other coelacanths [12], the basisphenoid was certainly pierced medially by the buccohypophyseal canal (?pit.fos, Figure 9C). However, it fails to pierce the ventral surface of the parasphenoid in AMNH FF 20267 (Figure 11B), contrary to what is observed in basal coelacanths such as *Diplocercides*, *Euporosteus* [12], *Miguashaia* [21], and *Styloichthys* [22], [23]. This condition observed in *Megalocoelacanthus* seems to be derived among coelacanths and also observed in *Axerodichthys* (AMNH 14026L), *Latimeria* (MNHN C24), *Macropoma* (figure 6.10 in [12]), *Undina* (BSPG 1870 XIV 508). In posterior and dorsal views, the basisphenoid of the holotype specimen CCK 88-2-1 presents an overlapping surface for the descending process of the parietal (o.v.pr.Pa, Figure 9B). The sphenoid condyles (sph.c, Figure 9A, 9C, 9D) are well separated by the concave posterior margin of the basisphenoid. The condition observed in *Megalocoelacanthus* is very similar to that of *Latimeria* [18] and *Macropoma* (figure 6.12B, D in [12]), rather than to that of *Mawsonia* or *Axelrodichthys* where the sphenoid condyles are very close medially and separated by a deep notch (figures 1A, 18A in [5]).

Two halves of the otoccipital portion are preserved, but extremely flattened (Figures 5, 6, 7, 8). The prootic (Pro, Figures 5, 6, 7, 8) is short, ventrally oriented, and shows a very short and narrow otic shelf whose inner surface is covered by tiny rugosities (ot.sh, Figures 6, 8). It is enlarged posteriorly and its posterior end is broken on both sides. The inclination of the otic shelf is less prominent than in *Macropoma*, but this may be due to deformations of the specimen.

The prootic presents two roughened areas. The anterior one (the so-called prefacial eminence) is sutured on the inner side with the postparietal descending process (v.pr.Pp, Figures 6, 8), and the posterior one is sutured with the supratemporal descending process (v.pr.Stt, Figures 5, 6, 7). This latter suture can be observed only on the lateral part of the right side (Figure 5). Between these two areas, temporal excavation is marked by a slight concavity on both sides (Figures 5, 7), and seems to be lined with bones like in *Axelrodichthys* and *Mawsonia* [4]. The condition observed in *Megalocoelacanthus* is thus different from that of *Latimeria* and *Macropoma*, where the temporal excavation is cartilaginous. In the inner part of both sides (Figures 6, 8), the suture between the descending process of the postparietal and the prootic runs anteroventrally from the dorsal edge of the otic shelf to the anterior margin of the otoccipital portion. The path of this suture is unclear on the lateral part of both sides because it is completely overlapped by the postparietal shield on the right side, and only the anteriormost portion of the suture can be observed on the left. However, it seems that the suture is directed posterodorsally on the lateral side. The contact between the prootic and the descending process of the postparietal (Figure 5) appears to be much more ventral that what can be observed in other coelacanths, but this is probably an artefact due to the strong deformation of this portion of the skull.

The course of the arteries and nerves through the prootic is quite variable among coelacanths [12]. In *Macropoma*, the lateral surface of the prootic is pierced by several foramina that are lacking in *Latimeria*: the palatine nerve emerges ventrally to the prefacial eminence, and the orbital artery opens ventral to the contact with the supratemporal descending process. The identification of such foramina is very difficult in *Megalocoelacanthus*. Most probably, evidence for a foramen could be found on the lateral face of the right moiety of the prootic (?f.o.a, Figure 5). Like in *Macropoma* and *Mawsonia* a foramen opens ventrally to the roughened area contacting the supratemporal descending process, which allows us to suggest that it could correspond to the foramen for the orbital artery.

Posterior to the otic shelf, the lateral wall extending posteroventrally is broken on both sides of the skull. The saccular chamber is completely flattened between the postparietal shield and the medial wall that usually separates it from the notochordal canal.

In coelacanths, the base of the otoccipital portion of the neurocranium is poorly ossified and composed of several elements that embed the notochord ventrally. The basioccipital alone is sutured to the posterior wing of the prootic, whereas the anazygal, and the anterior and posterior catazygals occupy the basicranial fenestra and lie free from the rest of the neurocranium. In *Latimeria*, these elements are firmly attached to the neurocranium by mean of strong ligaments (Dutel pers. obs. on MNHN C24). The anterior and the posterior catazygals are preserved in AMNH FF 20267 (Figure 10). The anterior catazygal (Figure 10A) is the largest one, semi-circular in shape, wider than long, with concave lateral margins. The posterior catazygal (Figure 10B) is much smaller, longer than wide, and bell-shaped in dorsal/ventral views. Its anterior margin is straight whereas the posterior is rounded and narrower. Although these elements are rarely preserved in fossil coelacanths, they are well known in *Mawsonia* [2], [3]. Anterior catazygals referred to this genus are butterfly-shaped whereas the posterior catazygal is semi-lunar in shape [2]. Catazygals are unossified in *Axelrodichthys* [2]. The catazygals described in *Megalocoelacanthus* are very different to that of *Mawsonia*, but rather resemble those of *Holophagus* (figure 6.9 in [12]) *Macropoma* (figure 6.10 in [12]) and *Latimeria* [18].

#### 1.4 Palate

The parasphenoid (Par, Figures 2, 3, 11) is very narrow and deep. As in *Latimeria*, its anterior half is marked by low and elongated lateral wings (a.w.Par, Figures 2, 3), which are connected to the ventral margin of the lateral ethmoid. In ventral view (Figure 11), the parasphenoid is strongly compressed laterally along its posterior half, and expands on both sides at the level of the anterior half. The anterior portion is ovoid in shape, ventrally concave and covered by tiny villiform teeth (t.Par, Figure 11). In lateral views (Figures 2, 3), the posterior half of the parasphenoid rises steeply, so that its posteriormost dorsal surface reaches the anterior margin of the processus connectens. This condition is unknown in *Macropoma*, *Latimeria* (figures 6.1, 6.11 in [12]) and other Mesozoic coelacanths such as *Axelrodichthys* (Maisey 1986), but is present in *Rhabdoderma* (figure 6.5 in [12]). The parasphenoid ends posteriorly abruptly below the anterior side of the processus connectens, with a slightly curved ventral margin.

The palatoquadrate (Figure 11A) consists of the pterygoid anteriorly, the metapterygoid posterodorsally, and the quadrate posteroventrally. The palatoquadrate is triangular in shape, short and very deep. Its general shape is thus proportionally similar to that of *Latimeria*, *Macropoma*, and *Holophagus* [12], whereas *Mawsonia* and *Axelrodichthys* possess a longer and shallower palatoquadrate [5]. The anteriormost part of both pterygoids (Pt, Figure 11A) of AMNH FF 20267 as well as that of the holotype specimen CCK 88-2-1 (figure 2F in [1]) is thin and covered with striations. In these specimens as well as in AUMP 3834, and FMNH P27524 (Schwimmer pers. obs.) the anterior termination is thus similar, suggesting that it was poorly ossified or capped by a cartilage layer that was connecting it to the autopalatines which are preserved separately in AMNH FF 20267 (Figure 11C). The pterygoid (Pt, Figure 11A) is triangular, shallow and short, and presents a ventral swelling (v.sw.Pt, Figure 11A) anterior to the quadrate that is more pronounced than that of *Macropoma* or *Latimeria*. The pterygoid forms a narrow and straight edge along the anterior margin of the metapterygoid. On the medial side of the palatoquadrate, the pterygoid also overlaps the metapterygoid and forms its posterior edge. The medial surface of the pterygoid is ornamented with tubercular shagreen.

The metapterygoid (Mpt, Figure 11A) is short but very large compared to that in other coelacanths. As in *Latimeria*, it is saddle-shaped and its dorsal surface was articulating with the antotic process (art.ant.pr, Figure 11A). When considering the entire palatoquadrate, its size is proportionally quite small. Contrary to what can be observed in *Mawsonia* or *Axelrodichthys*, the metapterygoid displays a marked ventral recess ventral to the dorsal edge of the pterygoid on the medial side.

The quadrate (Q, Figure 11A) is straight vertically and extends dorsally up to the level of the ventral half of the pterygoid. It finishes dorsally as an open-end prolonged by an anterodorsally curved ridge extending onto the pterygoid and metapterygoid. This suggests the presence of a posterior cartilage joining the dorsal end of the quadrate to the metapterygoid. The double condyle of the quadrate is large and robust, as in *Latimeria* and *Mawsonia*.

#### 1.5 Lower jaw, coronoids and gular plates

**Figure 12:**
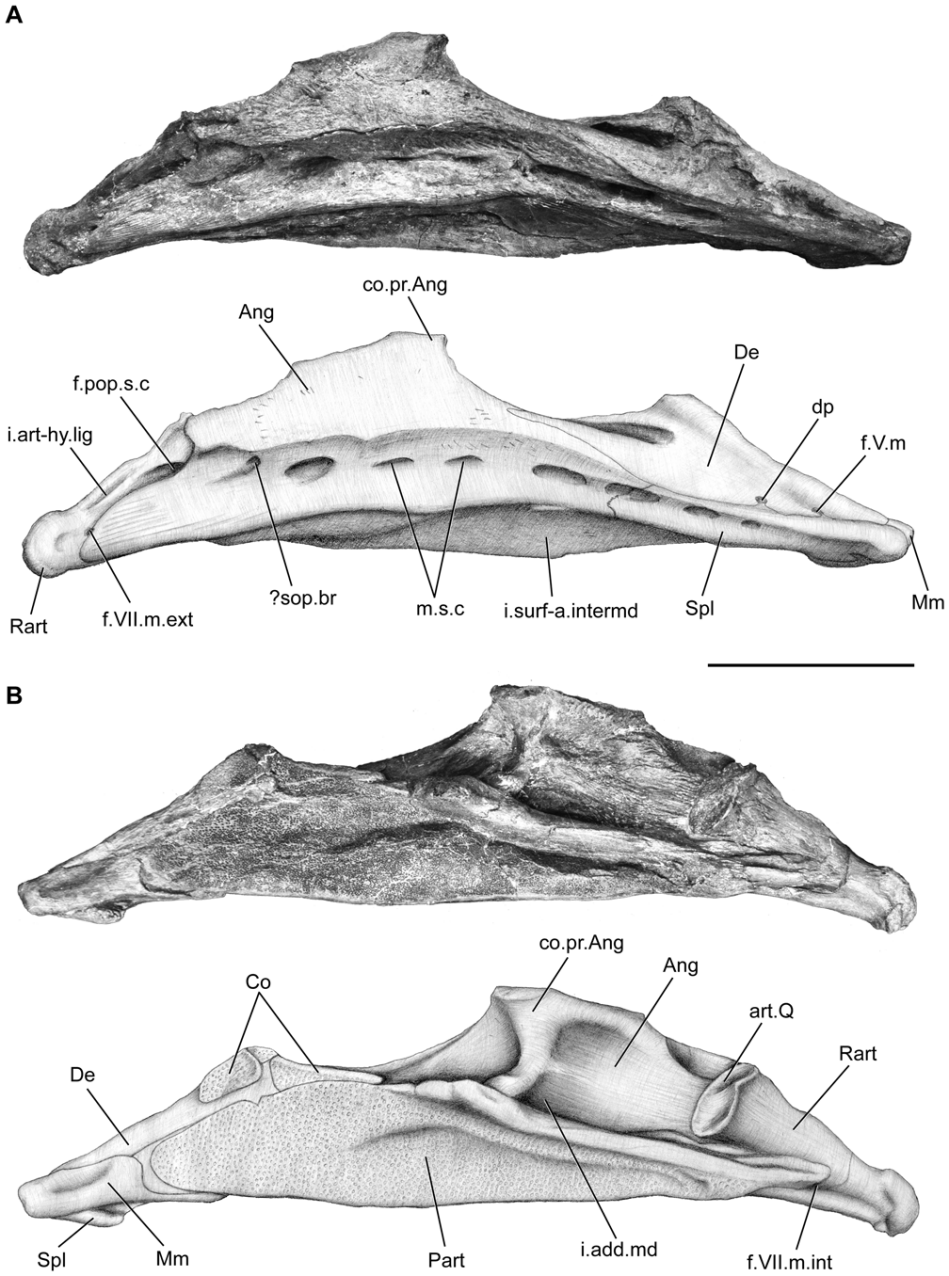
*Megalocoelacanthus dobiei* Schwimmer, Stewart & Williams, 1994, AMNH FF 20267 from lower Campanian of the Niobrara Formation. Right lower jaw. **A,** lateral view. **B,** medial view. Abbreviations: **Ang,** angular; **art.Q,** surface for articulation with the quadrate; **Co,** coronoids; **co.pr.Ang,** coronoid process of the angular; **De,** dentary; **d.p,** enlarged sensory pore within the dentary; **f.pop.s.c,** opening for the preopercular sensory line canal; **f.V.m,** foramen for the mandibular ramus of the trigeminal nerve; **f.VII.m.ext,** foramen for the external mandibular ramus of the facial nerve; **f.VII.m.int,** foramen for the internal mandibular ramus of the facial nerve; **i.add.md,** insertion point for the adductor mandibulae muscle; **i.art-hy.lig,** insertion point for the articular-hyomandibular ligament; **i.surf-a.intermd,** insertion surface for the anterior ramus of intermandibular muscle; **Mm,** mentomeckelian; **m.s.c,** mandibular sensory line canal; **Part,** prearticular; Rart, retroarticular; **?sop.br,** subopercular branch of the preopercular canal; **Spl,** splenial. Scale bar  = 10 cm.

**Figure 13:**
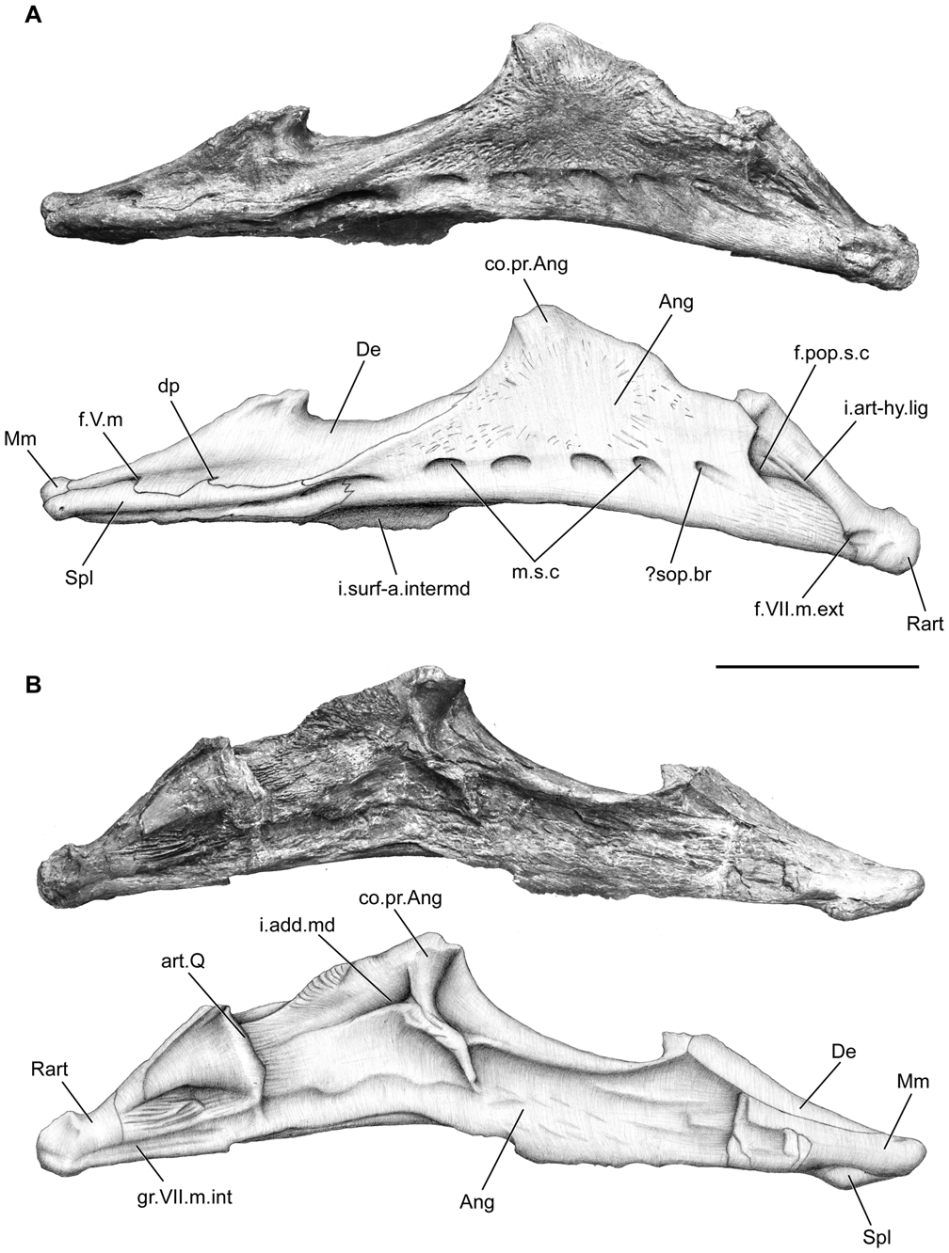
*Megalocoelacanthus dobiei* Schwimmer, Stewart & Williams, 1994, AMNH FF 20267 from lower Campanian of the Niobrara Formation. Left lower jaw. **A,** lateral view. **B,** medial view. Abbreviations: **Ang,** angular; **art.Q,** surface for articulation with the quadrate; **co.pr.Ang,** coronoid process of the angular; **De,** dentary; **d.p,** enlarged sensory pore within the dentary; **f.pop.s.c,** opening for the preopercular sensory line canal; **f.V.m,** foramen for the mandibular ramus of the trigeminal nerve; **f.VII.m.ext,** foramen for the external mandibular ramus of the facial nerve; **gr.VII.m.int,** groove for the internal mandibular ramus of the facial nerve; **i.add.md,** insertion point for the adductor mandibulae muscle; **i.art-hy.lig,** insertion point for the articular-hyomandibular ligament; **i.surf-a.intermd,** insertion surface for the anterior ramus of intermandibular muscle; **Mm,** mentomeckelian; **m.s.c,** mandibular sensory line canal; **Rart,** retroarticular; **?sop.br,** subopercular branch of the preopercular canal; **Spl,** splenial. Scale bar  = 10 cm.

Both lower jaws are well preserved (Figures 12, 13). The lower jaw is long and shallow throughout, and resembles that of *Undina*, *Holophagus*, *Latimeria* and *Macropoma*. The dentary (De, Figures 12A, 13A) is long and narrow, and its proportion relative to the total jaw length is close to that seen in *Undina* and *Holophagus*, where it reaches about 40% of the total jaw length [12]. The dentary overlaps the angular and possesses a hook-shaped process extending posterodorsally. The distal part of this hook-shaped process is broken, suggesting that it may have been more prominent and elongated, comparable to that of *Libys*, *Undina*, *Holophagus* and *Macropoma*. The splenial is narrow, and slender. It forms the ventral edge and ventrolateral side of the anterior portion of the jaw, and extends posteriorly to the hook-shaped process of the dentary (Figures 13A). It presents no traces of ornamentation on its surface. Ventrally, the splenial (Spl, Figures 12, 13) forms a hump that winds around the ventral edge of the mentomeckelian (Mm, Figures 12B, 13B). The mentomeckelian forms the anteriormost part of medial side of the jaw (Figures 12B, 13B). It is rectangular in shape, with a posterior finger-like expansion ventrally to the prearticular (Part, Figure 12B). It is inwardly curved, so that its anteriormost portion overlaps the splenial in lateral view (Figures 12A, 13A), to form the mandibular symphysis.

The angular (Ang, Figures 12A, 13A) is triangular in shape and slightly concave ventrally. It is deeper than in *Holophagus*, *Latimeria*, *Macropoma*, *Undina*, and *Swenzia*. Just behind its contact with the dentary, it shows a prominent dorsal, anteriorly bent extension that forms the coronoid process (co.pr.Ang, Figures 12, 13). The dorsal margin of the angular is concave posteriorly, and then runs straight anteriorly up to the blunt process. This condition is very different from that of the latimeriids *Latimeria*, *Macropoma*, and *Swenzia* where the dorsal edge of the angular is convex and regular throughout, and of the mawsoniids *Mawsonia* and *Axelrodichthys* where it forms a deep hump. The center of ossification of the angular can be observed at the level of its deepest portion. A long and broad surface for the insertion of the anterior and posterior ramus of the intermandibular muscle is seen on the ventral margin of lower jaw (i.surf-a.intermd, Figures 12A, 13A) as in *Latimeria*. No oral pit lines are observed on the angular.

**Figure 14:**
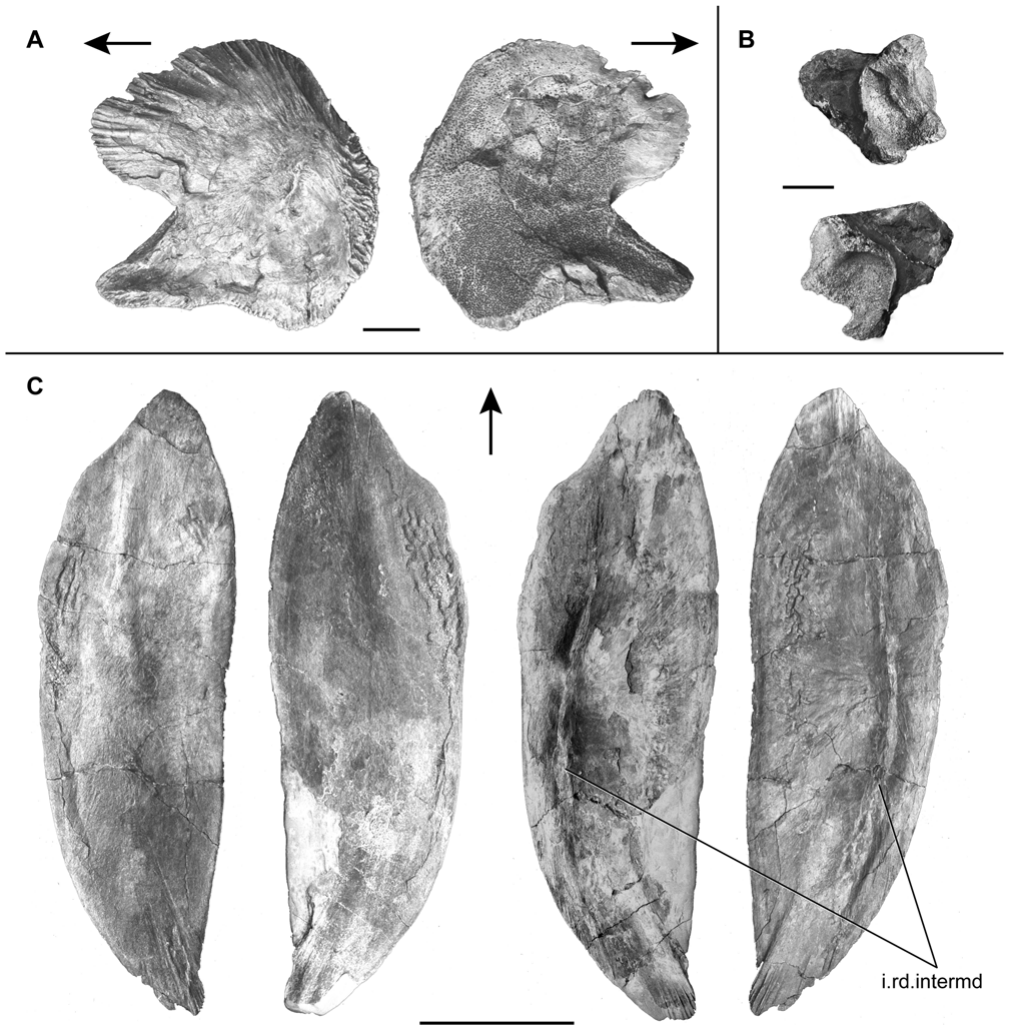
*Megalocoelacanthus dobiei* Schwimmer, Stewart & Williams, 1994, AMNH FF 20267 from lower Campanian of the Niobrara Formation. **A,** left principal coronoid in lateral (left) and medial (right) views. **B,** articulars of the left (top) and right (bottom) lower jaws. Scale bar  = 2 cm. **C,** gular plates in external (left) and internal (right) views. Abbreviation: **i.rd.intermed:** insertion ridge for the intermandibular muscle. Scale bar  = 10 cm. Arrows oriented anteriorly.

The coronoid series is poorly preserved and few elements of the series are only observed on the medial side of the right lower jaw (Co, Figure 12B). The coronoid posterior to the hook-shaped process of the dentary is elongated and closely associated with the dentary. The surface of the coronoid is covered with shagreen tubercles, and does not bear any enlarged teeth. Anteriorly to the hook-shaped process of the dentary, only a smaller coronoid is preserved. However, the rugous dorsal surface of the dentary suggests that the coronoid series were extending anteriorly up to the level of the mentomeckelian. The left principal coronoid (Figure 14A) is preserved as an isolated element. Its general shape is rounded with a broad base that extends posteriorly as a finger-like process. The dorsal portion of the principal coronoid is curved and much longer than in *Macropoma* and *Libys* where it is developed as a narrow dorsal process [12]. Deep furrows mark the entire length of its ventrolateral edge. Digitations are present on the posterodorsal edge, from which long grooves extend radially. The medial surface of this coronoid is covered by tiny, villiform teeth that form a shagreen area.

The prearticular is preserved only on the right jaw (Part, Figure 12B). It consists in a slender, long and shallow bone that covers most of the medial side of the lower jaw, and like the principal coronoid, is covered with tiny villiform teeth. The articulars (Figure 14B) are preserved as isolated elements and consist of a single ossification. They possess two concave articulatory facets, one dorsal and one ventral that are anteromedially inclined. The retroarticular (Rart, Figures 12B, 13B) possesses a double articulatory facet that faces the articular. Posteriorly, the retroarticular bears a small facet for the articulation of the symplectic. As in other coelacanths, the posterior portion of the retroarticular may have been capped with cartilage. A ridge interpreted as the insertion point for the articular-hyomandibular ligament is observable on the lateral surface of the retroarticular (i.art-hy.lig, Figure 12). Longitudinal ridges are present on the medial surface of the retroarticular.

The course of the main sensory line canal in *Megalocoelacanthus* seems to be very similar to that observed in *Latimeria* and *Macropoma*. The mandibular sensory line canal is a large canal that opens ventrally through large pores on the surface of the angular and splenial (m.s.c, Figures 12A, 13A). The pores are oriented ventrally on both bones. Four pores are clearly observed on the splenial and five on the angular. The anteriormost pores open on the angular are oriented anteroventrally whereas the posterior ones are oriented posteroventrally. Posteriorly, a marked notch is present at the margin of the angular, lateral and posterior to the articular glenoid. A foramen can be clearly observed in this area on the right mandible of AMNH FF 20267 (f.pop.s.c, Figure 13B) as well as on holotype CCK88-2-1, suggesting that the main mandibular sensory line canal runs down from the preopercular and enters the angular at this level, as in *Latimeria* [18], [19], [24], [25] and *Macropoma* [12]. Anteriorly, a long and slender foramen lies along the suture between the splenial and the dentary at the level of the hook-shaped process of the dentary (dp, Figures 12A, 13A). Such a foramen is also present in the same position in *Macropoma, Holophagus* and *Undina* [12]. This foramen could correspond in *Latimeria* to the enlarged pore of the sensory line canal that pierces the splenial just beneath the suture with the dentary. This pore is connected to the main mandibular canal that runs through the angular. In *Macropoma* and *Holophagus*, the subopercular branch of the preopercular canal exits posteriorly on the angular immediately beneath the foramen for the external ramus of the mandibular branch of the facial nerve [12]. Such a foramen has not been observed in this area in *Megalocoelacanthus*. If the subopercular branch of the preopercular is present in *Megalocoelacanthus*, the posterior orientation of the posteriormost pore in the angular would mark its point of exit (?sop.br, Figures 12A, 13A). This condition would thus be similar to that in *Latimeria* [18].

Anterior to the enlarged pore of the sensory line canal, another foramen pierces the dentary just above the suture with the splenial (f.V.m, Figures 12A, 13A). As in *Latimeria*, this foramen may correspond to the exit of the mandibular branch of the trigeminal nerve for innervating the skin of the lower lip. The relative position of the enlarged pore of the sensory line canal and the foramen for the mandibular branch of the trigeminal nerve differs from that of *Latimeria*. In this genus the enlarged pore of the sensory line canal is ventral to the foramen for the mandibular branch of the trigeminal nerve, whereas both are aligned antero-posteriorly along the dentary-splenial suture in *Megalocoelacanthus*.

A foramen for the external mandibular ramus of the facial nerve (f.VII.m.ext, Figures 12A, 13A) is present on the lateral surface of the jaw between the retroarticular and the angular. In *Latimeria*, the internal ramus of the mandibular branch of the facial nerve penetrates the mandible by a foramen situated between the prearticular and the retroarticular. In *Megalocoelacanthus* a ventral groove can be observed on the anterior margin of the retroarticular, at the level of the articulation facet (Figures 12B, 13B). The absence of the prearticular on the left lower jaw shows the course of this groove within the mandible (gr.VII.m.int, Figure 13A). Forey [12] also observed such a groove in *Macropoma* and interpreted it as the mark of the path of the internal mandibular ramus of the trigeminal nerve within the mandible.

Both gular plates are preserved with little deformation (Figures. 14C). Their shape is very similar to those of *Latimeria*, and their surface is slightly concave dorsally. Their anterolateral edge is slightly swollen and the anterior tips diverge strongly along the posterior midline. The lateral edge of the gular plates is curved in its posterior half, so that the posterior tips meet medially. On the internal surface of the gular plate, a ridge runs along the anteroposterior axis parallel to the lateral edge (i.rd.intermd, Figures. 14C). In *Latimeria*, this ridge corresponds to the insertion point of the anterior and posterior ramus of intermandibular muscle (Dutel pers. obs. on MNHN C24). Neither their internal nor their dorsal surfaces are ornamented and the gular pit-lines are not observed.

#### 1.6 Cheek bones and opercular

Cheek bones are poorly preserved in AMNH FF 20267. An isolated element presenting a sensory line canal crossed by pillars could be interpreted as a fragment from the lachrymojugal or the postorbital. This suggests that the sensory line canal was opening through cheek bones by large vacuities as on the skull roof. Consequently, the condition in *Megalocoelacanthus* would have been identical to that of *Libys* were the large sensory line canal is opening through a large continuous groove crossed by pillars on the lachrymojugal and postorbital (BSPG 1860 XIV 502). Like in this genus and other latimerioids [12], it is probable that cheek bones were well separated from each other in *Megalocoelacanthus*, explaining the poor preservation of this complex.

**Figure 15:**
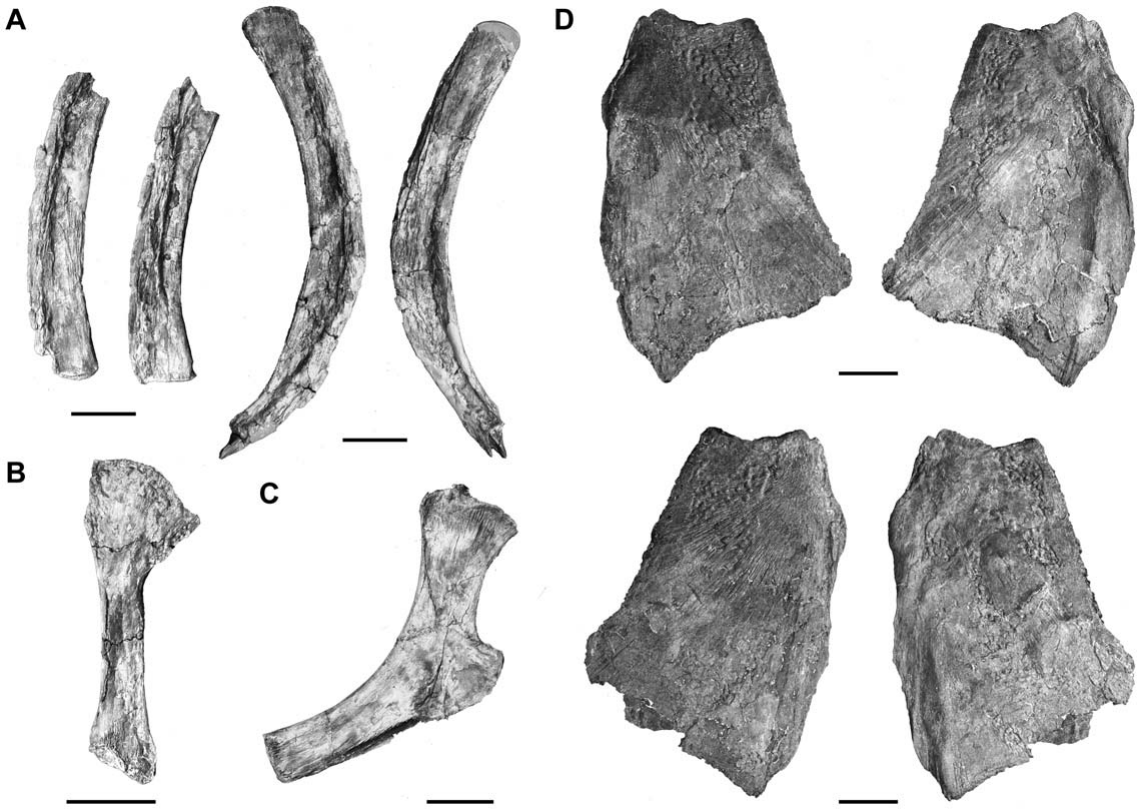
*Megalocoelacanthus dobiei* Schwimmer, Stewart & Williams, 1994, AMNH FF 20267 from lower Campanian of the Niobrara Formation. **A,** branchial arches; **B,** symplectic; **C,** ceratohyal; **D,** left (top) and right (bottom) operculars in lateral (left) and medial (right) views. Scale bar  = 5 cm.

Both operculars are preserved (Figure 15D). They are partly broken posteriorly and ventrally, but seem to be deeper than long as in *Latimeria*, *Holophagus*, and *Macropoma*. Their center of ossification is situated anterodorsally, and both their lateral and medial surfaces are ornamented by isolated tubercles. The anterodorsal edge of the operculars is notched and similar in shape to that of *Macropoma* and *Holophagus* [12].

#### 1.7 Hyoid arch

The hyoid arch of coelacanths consists of hyomandibular, interhyal, ceratohyal, hypohyal and symplectic. Only one ceratohyal and the left symplectic are preserved here.

The left symplectic (Figure 15B) is entirely preserved but strongly compressed laterally. Its shape is typical, with an upper part enlarged posteriorly. As in other coelacanths, both ends were probably cartilaginous and its actual length may have been longer.

The ceratohyal (Figure 15C) has a typical shape for coelacanth. It is laterally compressed, curved posteriorly, and its posteriorly directed elbow-like expansion is hook-shaped like in *Axelrodichthys* (AMNH 13962 R), *Latimeria* [18], *Macropoma* [12] and *Mawsonia* (AMNH 11758). The ventral expansion is slender and slightly convex, and its posterior portion bears a groove that may have been capped with cartilage, as well as the anterior margin of the bone that finishes in a dead-end. In AMNH FF 20267 as well as in the holotype specimen CCK 88-2-1 (figure 2L in [1]), the ceratohyal is much more curved, and its ventral expansion appears to be shorter than that of *Axelrodichthys* (FMNH FM11856), *Latimeria* [18], and *Mawsonia* (AMNH 11758). The dorsal end and the elbow-like expansion are broken, but they are usually also capped by cartilage.

#### 1.8 Branchial arches, urohyal

The branchial skeleton is poorly known in fossil coelacanths. The branchial skeleton of *Megalocoelacanthus* consists of a complete urohyal, and a basibranchial associated with tooth plates and ceratobranchials.

**Figure 16:**
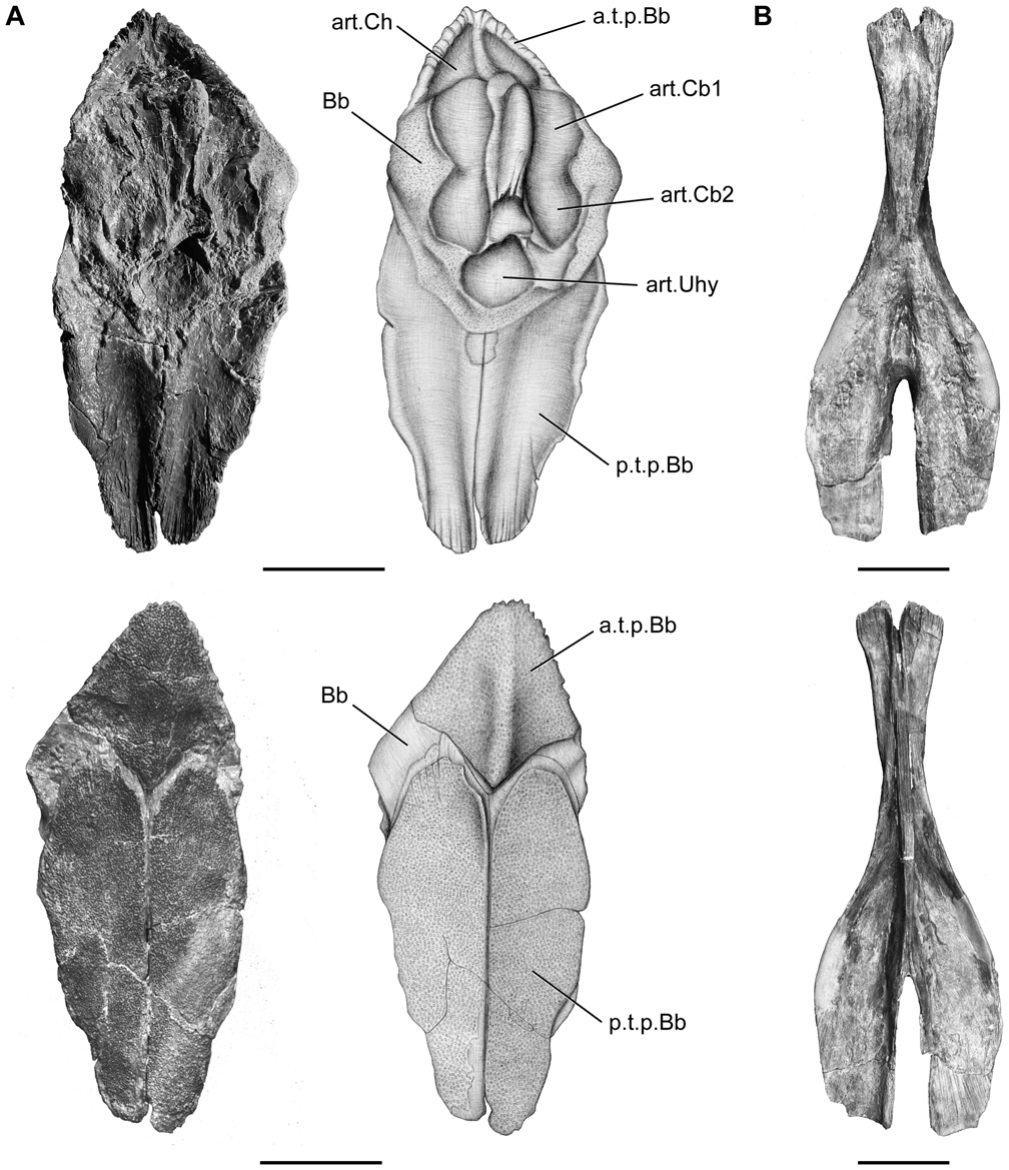
*Megalocoelacanthus dobiei* Schwimmer, Stewart & Williams, 1994, AMNH FF 20267 from lower Campanian of the Niobrara Formation. **A,** basibranchial and basibranchial tooth plate in dorsal view (top), and ventral view (bottom). **B,** urohyal in dorsal view (top), and ventral view (bottom). Abbreviations: **a.t.p.Bb,** anterior tooth plate of the basibranchial; **art.Cb1,** surface for articulation with the first ceratobranchial; **art.Cb2,** surface for articulation with the second ceratobranchial; **art.Ch,** surface for articulation with the ceratohyal; **art.Uhy,** surface for articulation with the urohyal; **Bb,** basibranchial; **p.t.p.Bb,** posterior tooth plate of the basibranchial. Scale bar  = 5 cm.

The basibranchial (Bb, Figure 16A) is large and rounded in shape. It is fused with three tooth plates (p.t.p.Bb, a.t.p.Bb, Figure 16A). As in *Macropoma* [12], the basibranchial consists in a central embedded ossification surrounded by a perimeter of cartilage. Its ventral surface is marked by a posterior median pit for the articulation of the urohyal (art.Uhy, Figure 16A), and by paired lateral pits that were probably articulated with the first two ceratobranchials as in *Latimeria* and *Macropoma* (art.Cb1, art.Cb2, Figure 16A). Tooth plates associated with the basibranchial consist in an anterior median diamond-shape plate (a.t.p.Bb, Figure 16A) and a pair of large plates, extending symmetrically posteriorly along the midline (p.t.p.Bb, Figure 16A). The ventral surface of the latter displays a paired concavity that is interpreted for the articulation of the ceratohyal (art.Ch, Figure 16A). Tiny villiform teeth cover all the tooth plates. Although basibranchial tooth plates are rarely found *in situ*, previous studies support that the trend in basibranchial tooth plate evolution in coelacanths was towards a mid-line fusion of many paired plates into larger tooth plates [12], [26]. The pattern observed in *Megalocoelacanthus* is also observed in *Latimeria* and *Undina* where the tooth plates associated with the basibranchial consist of large paired plates posteriorly to a smaller median plate, while *Diplurus* and *Axelrodichthys* bear, respectively, three and two pairs of large plates [12].

The urohyal (Figure 16B) is very well preserved and shows no evidence of strong deformation. The general shape of the urohyal is characteristic of what can be observed in other coelacanths. The bifid posterior end is broad and form two semi-lunar wings. They arise at the level of the first third of the bone, making them more prominent than those of *Latimeria* and *Macropoma*. The slit between the posterior wings is straight, narrow and prolonged anteriorly by a broad groove. These conditions are thus different from that observed in *Axelrodichthys* (FMNH FM11856) in which the lateral wing extends straightly and the slit is V-shaped. The slit is more expended anteriorly than that of *Macropoma* (figure 7.7 in [12]) and *Latimeria* (figure 7.6 in [12]). The ventral surface of the urohyal is flat, except on its anterior portion where the edges are slightly raised. The anterior end is also bifid with a hump on each side. The dorsal surface of the posterior wings is concave in its anterior portion and flat in its posterior portion. A long and deep septum covered by small grooves extends medially. Although some notable differences are observed, the general shape of the urohyal of *Megalocoelacanthus* is much more similar to that of the latimeriids *Macropoma* and *Latimeria*, than to that of the mawsoniids *Mawsonia* and *Axelrodichthys*.

**Figure 17:**
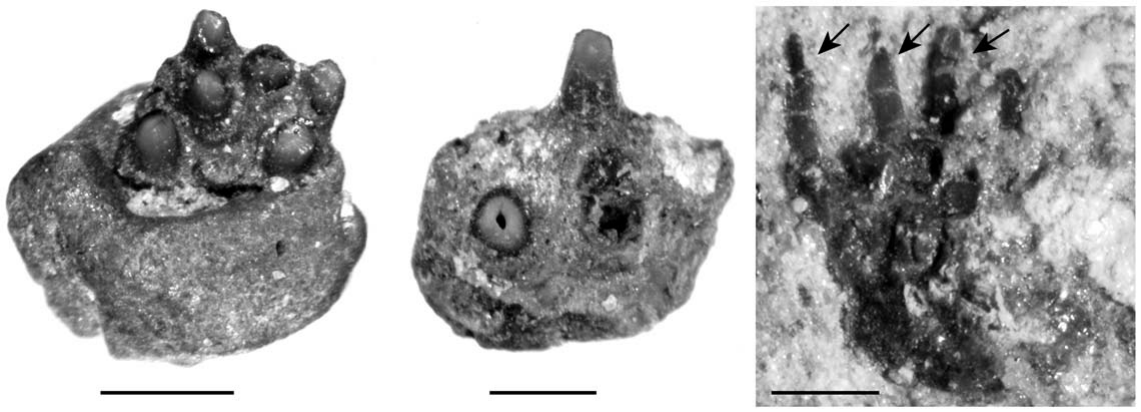
*Megalocoelacanthus dobiei* Schwimmer, Stewart & Williams, 1994, holotype specimen CCK 88-2-1 from lower Campanian of the Blufftown Formation. Close-up view of branchial denticles present on the edge of the gill arches of the holotype specimen. Arrows indicate the denticles embedded in the matrix. Scale bar  = 1 mm.

As usual in fossil coelacanths, little remains of the gill arches. Four ceratobranchials are preserved (Figure 15A), and two of them are almost complete. However, it is difficult to determine their position in the branchial series. Ceratobranchials are curved and compressed laterally. The ventral extremity is rounded and was probably articulated with the basibranchial. The bone narrows dorsally into a sharp dorsal end. Isolated small, sharp, denticles measuring about 1–2 mm are preserved in the matrix at the edge of the ceratobranchial of the holotype specimen CCK 8-22-1 (Figure 17). These denticles were recognized as branchial teeth by Schwimmer [27] and we here follow this interpretation. When compared to the size of the ceratobranchial, these denticles are significantly smaller than those of *Latimeria* and *Axelrodichthys* (FMNH FM11856).

#### 1.9 Postcranial skeleton and scales

Very few elements of the postcranial skeleton are preserved. All axial skeleton and fins are missing, and only the pectoral girdle and some isolated scales are present.

**Figure 18:**
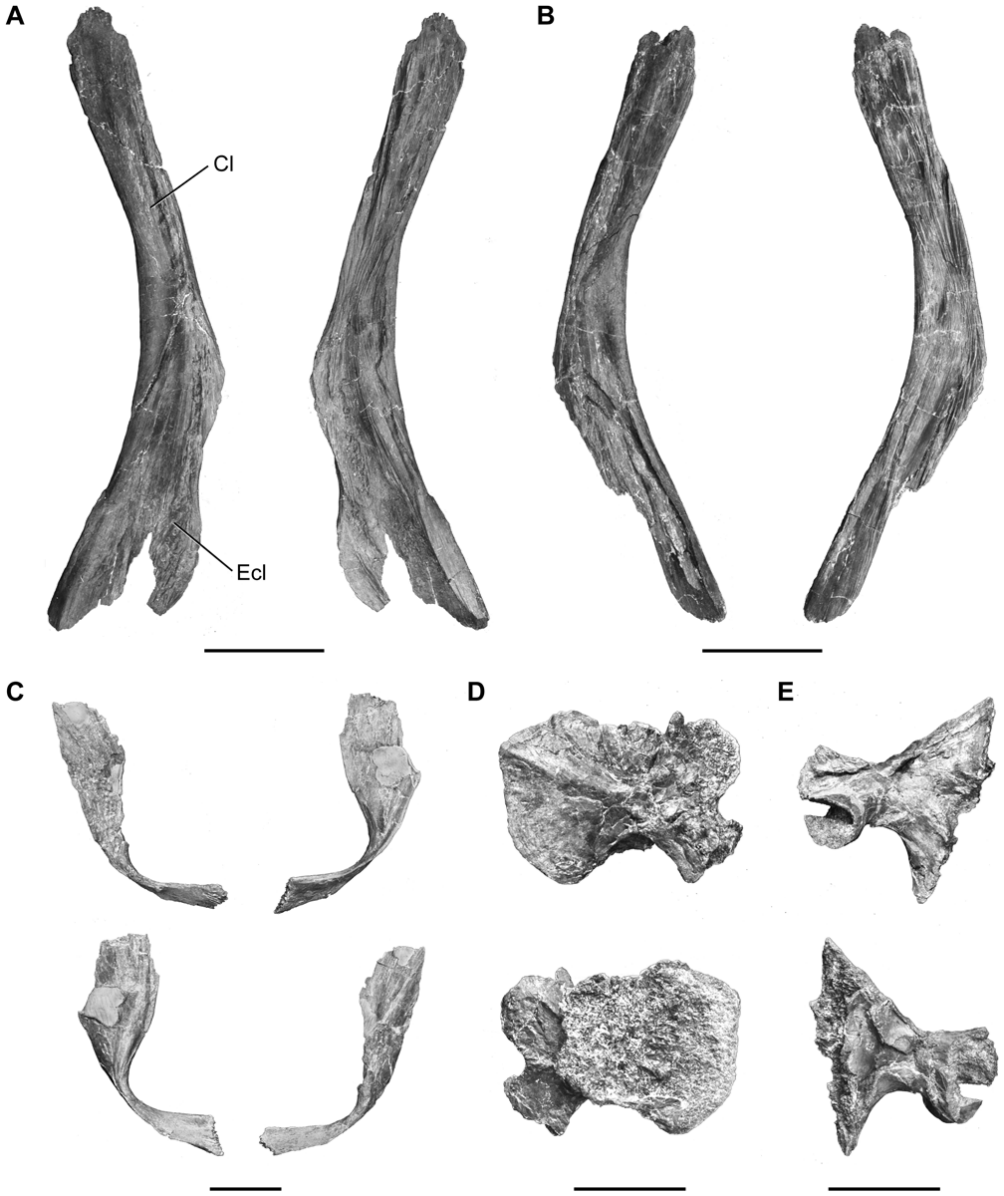
*Megalocoelacanthus dobiei* Schwimmer, Stewart & Williams, 1994, AMNH FF 20267 from lower Campanian of the Niobrara Formation. Shoulder girdle. **A,** Left cleithrum in lateral (left) and medial (right) views. **B,** right cleithrum in lateral (left) and medial (right) views. **C,** clavicle in posterior (top) and anterior (bottom) views. **D,** right scapulocoracoid in medial (top) and lateral (bottom) views. **E,** left scapulocoracoid in medial (top) and lateral (bottom) views. Abbreviations: **Cl,** cleithrum; **Ecl,** extracleithrum. Scale bar  = 10 cm (**A–B**), 5 cm (**C–E**).

The shoulder girdle (Figure 18) is narrow and is represented by the cleithrum, the extracleithrum, the clavicle and the scapulocoracoid. The pectoral girdle of coelacanths usually includes one supplementary dermal bone, the anocleithrum, which is not preserved here.

The cleithrum (Figures 18A, B) is compressed laterally and bent anteriorly. The left cleithrum (Figure 18A) is the best preserved with the extracleithrum sutured on the lateral side, and only lacks its dorsal and ventral tips. It is elbow-shaped posteriorly and presents a ventral and dorsal half very distinct in shape. The latter is slender, and more developed and straight than in *Latimeria* [18], whereas the former is broad and rounded posteriorly. Contrary to what can be observed in *Mawsonia* and *Axelrodichthys* [12], there is no broad medial extension of the cleithrum, and its general shape is more similar to what can be observed in *Latimeria* or *Macropoma*.

The most remarkable feature is the very small size of the clavicle (Figure 18C) compared to that of the cleithrum. As is typical, the clavicle twists medially from the leading edge of the cleithrum. The distal tip of the clavicle presents marked, irregular digitations, and the contact with its antimere was probably made through cartilage as in *Latimeria* [18]. Dorsally, the clavicle enlarges as a thin, medially concave layer of bone that was overlapping the lateral side of the cleithrum.

Both scapulocoracoids are preserved, but strongly flattened (Figures 18D, E). Each consists of a single, ossified element with a broad and flat proximal portion articulated with the cleithrum, and a short distal portion bearing the glenoid surface for the first axial mesomere. Although it is partially crushed, the best preserved scapulocoracoid presents a concave glenoid surface, covered with coarse rugosities, and which may have been capped with cartilage in life (Figure 18E).

**Figure 19:**
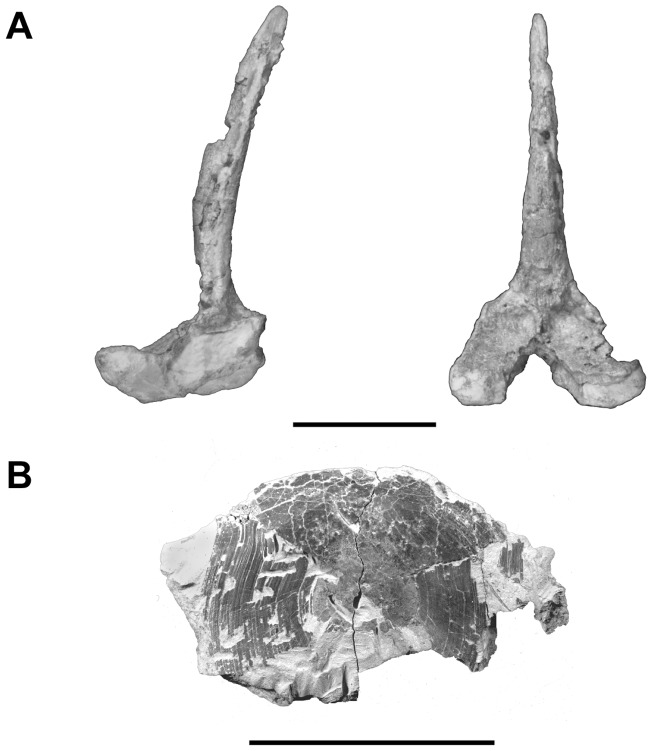
*Megalocoelacanthus dobiei* Schwimmer, Stewart & Williams, 1994. **A,** neural spine of the holotype specimen CCK 88-2-1 from lower Campanian of the Blufftown Formation in lateral (left) and anterior (right) views. **B,** scale of AMNH FF 20267 from lower Campanian of the Niobrara Formation. Scale bar  = 5 cm.

The axial skeleton of *Megalocoelacanthus* is only known from an isolated and well preserved vertebra found on the holotype specimen CCK 8-2-22 (Figure 19A). It is very similar to that of other coelacanths: the neural arch is forked and co-ossified with a median neural spine. In coelacanths, the anterior neural spines are short, and gradually increase in height posteriorly. Here, the neural spine is bended posteriorly and relatively short compared to those situated posteriorly to the D1 in other coelacanths. Thus, the vertebra preserved here was most probably situated anteriorly along the body axis.

The best-preserved body scale of AMNH FF 20267 is subcircular, and is about 5 cm in diameter (Figure 19B). Only its overlapped portion is preserved. As in other coelacanths, the exposed portion seems to represent here less than one third of the total surface of the scale. The scale shows concentric ridges and presents no evidence of pore canal system or lateral line system.

### 2. Phylogenetic analysis

#### 2.1 Revised matrix with new characters and taxa

The recent phylogenetic analyses investigating the relationships of coelacanths were made by Clément [8], Friedman & Coates [13], Yabumoto [14], Geng *et* *al*. [9], and Wendruff & Wilson [28] and are based on the data matrix of Forey [12] with several corrections and additions. Clément (2005) corrected scoring for character 31 “preopercular absent (0), present (1)” in the original Forey's matrix. Yabumoto (2008) subsequently changed Clément's scoring for *Mawsonia* to “1”. According to Friedman & Coates [13], the state of character 54 “dentary teeth fused to the dentary (0)” cannot be assessed for *Allenypterus* because its mandible is edentulous, and has to be scored as question mark. We here follow these scorings. Character 52 “sclerotic ossicles absent (0), present (1)” was scored as “1” for *Libys* by Forey [12]. This character is here scored as “0” because the respective holotypes of *L. superbus* and *L. polypterus* do not present sclerotic ossicles [29]. Forey [12] and other authors [5], [17], [21], [29] considered the visceral calcified structure present in fossil coelacanths as a calcified swim bladder. However, recent histological studies on the structure of the calcified organ in different specimens of *Axelrodichthys* and comparison with the fatty organ of the extant coelacanth *Latimeria*, suggest that this is instead an ossified bladder with a respiratory function rather than a buoyancy function [30]. Considering this interpretation we modified character 107 “swim bladder not ossified (0), swim bladder ossified (1)” into “ossified bladder absent (0), present (1)”. We also reviewed the coding of this character for several taxa: it was coded “0” for *Polyosteorhynchus*, *Allenypterus*, and “?” for *Mawsonia* [12]. We here code this character “1” for these three genera based on Lund & Lund [17] and Brito *et* *al*. [30]. Character 23 “supraorbital sensory line canal opening through bones as a single large pore (0), bifurcating pores (1), many tiny pores (2)” was scored “0” for *Libys*, *Latimeria*, *Diplurus*, *Laugia*, and *Whiteia*. The supraorbital sensory line canal of *Libys* and *Megalocoelacanthus* is a continuous groove crossed by pillars that are probably formed by the supraorbitals. This condition is thus very different from that of *Latimeria* (figure 3.1 in [12]), *Diplurus* (figure 4 in [31]), *Laugia* (figure 3.8 in [12]), and *Whiteia* (figure 3.15 in [12]) and we do not consider it can be coded under the same state of character. Consequently, we integrated an additional state to character 23: “supraorbital sensory line canal opening through bones as a single large pore (0), bifurcating pores (1), many tiny pores (2), a large and continuous groove crossed by pillars (3)”. Character 50 “Infraorbital, jugal and preopercular sensory canals opening through many tiny pores (0), opening through a few large pores (1)” was coded “1” for *Libys*. However, in this genus (BSPG 1870 XIV 502) the infraorbital and preopercular sensory line canal displays the same condition as the supraorbital sensory canal, i.e. a large and continuous groove crossed by pillars. Based on an isolated element of the lachrymojugal, the same condition was probably present in *Megalocoelacanthus*. We thus propose an additional state for character 50 “Infraorbital, jugal and preopercular sensory canals opening through many tiny pores (0), opening through a few large pores (1), a large, continuous groove stretched by pillars (2)”.

**Figure 20:**
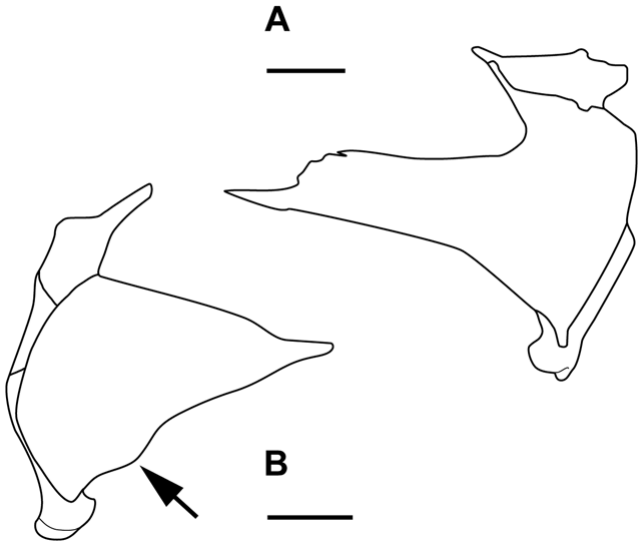
Comparison of the palatoquadrate of two actinistians showing the presence/absence of the ventral swelling of the pterygoid (arrow) coded as character 110. **A,** *Axelrodichthys*, right palatoquadrate in medial view (modified from Maisey 1986). **B,** *Latimeria*, left palatoquadrate in medial view (modified from Forey 1998). Scale bar  = 20 mm.

Additional characters and taxa are added in the matrix taken from Forey [12]. Character 109 “ventral keel scales absent (0), present (1)”, was proposed by Friedman & Coates [13]. Furthermore, we propose one new character: character 110 “ventral swelling of the palatoquadrate absent (0), present (1)” (Figure 20).

Finally, the data matrix includes the coelacanths recently described: *Piveteauia* [32], *Swenzia* [8], *Holopterygius* [13], *Parnaibaia* [14], *Guizhoucoelacanthus* [9], *Rebellatrix* [28], and several taxa (*Axelia*, *Euporosteus*, *Indocoelacanthus*, *Lualabaea*, *Ticinepomis*, and *Wimania*) that were coded by Forey [12] but excluded from his final analysis because of their high amount of missing data or the instability they were raising in the topology.

#### 2.2 Searching methods

The data matrix (Information S2) was constructed in Mesquite 2.74 [33]. It comprises 39 taxa and 110 anatomical characters including 88 cranial and 22 postcranial anatomical characters (Information S1). Maximum parsimony analyses were carried out using the software PAUP 4.0b10 [34]. A heuristic search was performed using the tree-bisection-reconnection branch swapping algorithm (TBR) with 10,000 random addition-sequence replicates. We ran the analysis with all characters unweighted and multistate characters unordered. Branches with a maximum length of zero were collapsed, so that any branch supported by ambiguous synapomorphies is conserved. Bootstraps values were calculated with this program using heuristic searches and 1,000 bootstrap replicates, with 100 random sequence additions per replicate. Bremer decay indices were calculated by combining PAUP 4.0b10 [34] and TreeRot v.3 [35]. Optimizations of ambiguous states of characters were performed using the software WINCLADA 1.00.08 [36]. The tree is rooted by two outgroups, porolepiforms and actinopterygians.

**Figure 21:**
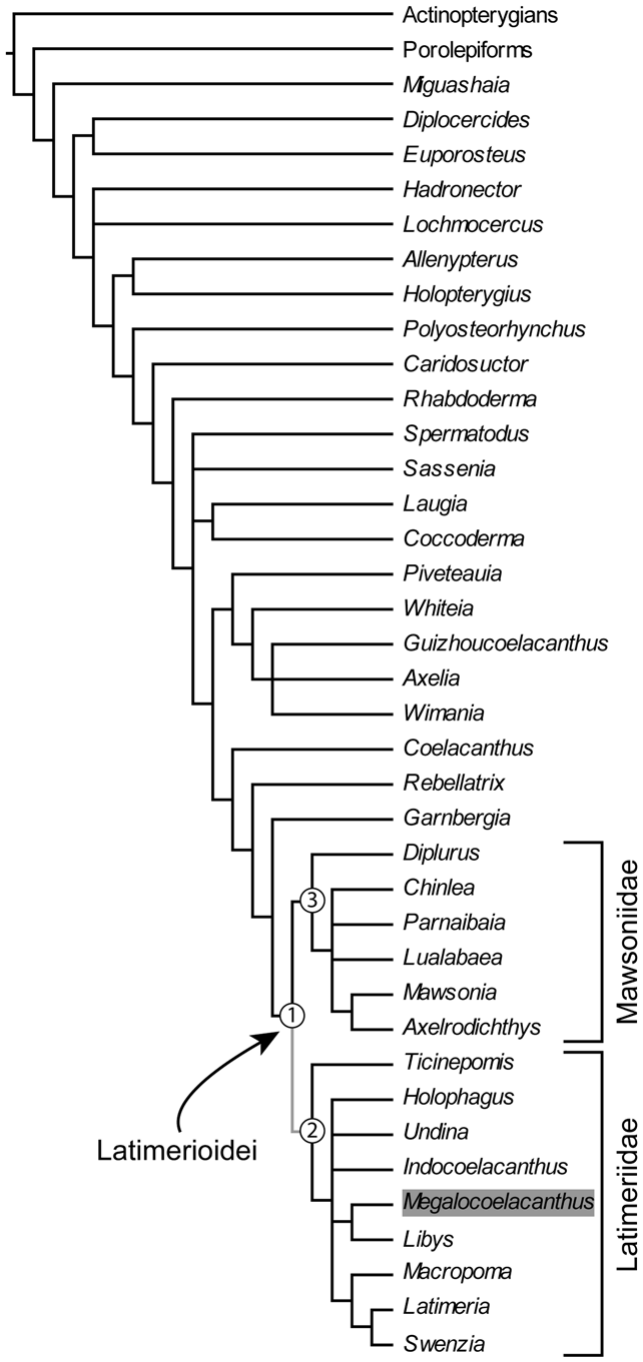
Result of the first phylogenetic analysis based on 39 taxa and 110 characters. Strict consensus tree of the 584 equally parsimonious trees (length  = 288; consistency index  = 0.4132; retention index  = 0.6938). Branch in grey is supported only by ambiguous synapomorphies.

**Figure 22:**
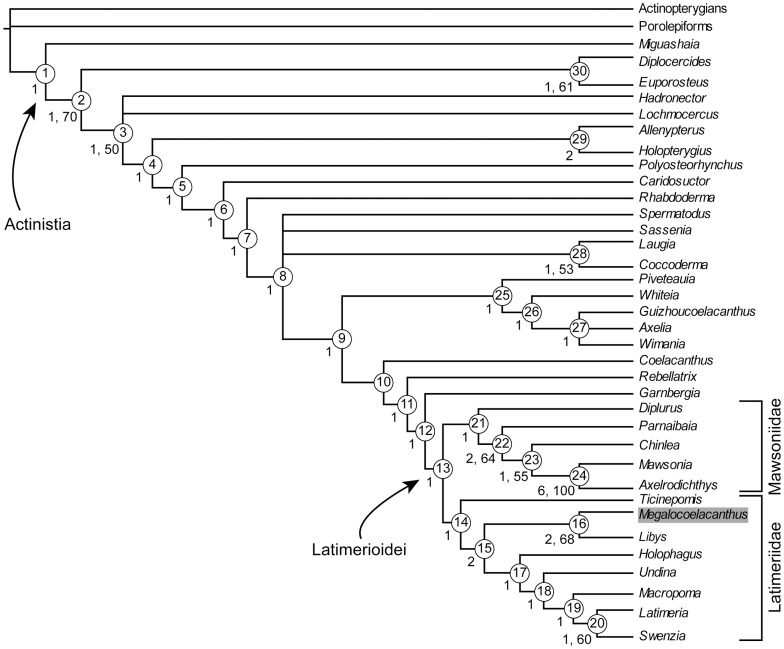
Result of the second phylogenetic analysis based on 37 taxa and 110 characters. Strict consensus tree of the 22 shortest trees (length  = 287; consistency index  = 0.4146; retention index  = 0.6929). Nodes are numbered from 1 to 30, and the list of apomorphies for each node and terminal taxon is given in Information S5. Numbers on the left of the node indicate the Bremer decay indices. Bootstrap values are indicated after the Bremer decay indices if superior to 50%.

Two analyses were carried out. The first analysis (Information S3) was run with all the taxa of the data matrix. The strict consensus tree (Figure 21) of the 584 equally parsimonious trees (length  = 288; consistency index  = 0.4132; retention index  = 0.6938) showed two areas of conflict within clade 2 and clade 3. The irresolution of the phylogenetic relationships is due to the instability of *Indocoelacanthus* (clade 2), and *Lualabaea* (clade 3). The instability of these taxa has already been noted by Forey [12] and attributed to the high percentage of missing values (*Indocoelacanthus*, 80%; *Lualabaea*, 99%) due to incompleteness of the fossil material. A second analysis was then conducted without these two taxa (Information S4). The strict consensus tree is shown Figure 22, and it will be used for presenting the following results. The diagnostic information for the nodes and terminal taxa are presented in Information S5.

#### 2.3 Phylogenetic results

The topology of the strict consensus tree (Figure 22) obtained from the 22 shortest trees (length  = 287; consistency index  = 0.4146; retention index  = 0.6929) places *Megalocoelacanthus* as the sister-taxon of *Libys* within clade 16. Three unambiguous synapomorphies support the node [*Megalocoelacanthus* + *Libys*]: a supraorbital sensory line canal opening through a large and continuous groove (23[3], that is a non-homoplastic synapomorphy), the infraorbital, jugal, and preopercular sensory line canals opening through a large and continuous groove crossed by pillars (50[2]), and a robust prearticular and principal coronoid, marked with fine striations (68[1]). Additionally, clade 16 is supported by six ambiguous synapomorphies: the presence of snout bones consolidated (2[1]), the presence of a preoperculum developed as a posterior tube-like canal-bearing portion and an anterior blade-like portion (39[1]), the absence of ornamentation upon cheek bones (49[0]), the optic foramen lying within an interorbital ossification or cartilage separate from the basisphenoid (70[1]), the presence of a forked anocleithrum (89[1]). The ambiguity arises from the fact that characters 2 and 70 are known in *Megalocoelacanthus* but not in *Libys*, and that characters 39, 49 and 70 are known in *Libys* but not in *Megalocoelacanthus*. Depending on the optimization the state of each character cited can be a synapomorphy of clade 16 (FAST optimization) or an autapomorphy of the terminal taxa in which it is known (SLOW optimization).

The question of the phylogenetic affinities of *M. dobei* with other coelacanths sheds a new light on the phylogenetic position of its sister-taxon. Forey [12] supported a sister-group relationship with the clade [*Diplurus* + *Mawsonia*] based on a single homoplastic synapomorphy: the reduction of the ornament (49[0]). The position of *Libys* was undetermined within Latimerioidei in Clément [8] and Friedman & Coates [13]. Clément [8] presented different topologies where *Libys* was alternatively the sister-group of the clade [*Diplurus* [*Chinlea* [*Mawsonia* + *Axelrodichthys*]]], the sister-group of the clade [*Macropoma* [*Swenzia* + *Latimeria*]], or the sister-group of *Garnbergia*. The last two hypotheses are corroborated in our topology, suggesting that *Libys* is more closely related to *Latimeria* than to *Mawsonia*. However, these last two topologies suggested that *Latimeria* is more closely related to *Libys* than it is to both *Holophagus* and *Undina*. This is inconsistent with our results and those of Geng *et* *al*. [9] where *Libys* is the sister group of a clade including *Holophagus*, *Undina*, *Macropoma*, *Swenzia* and *Latimeria*.

Our analysis provides new insights in the interrelationships of Latimerioidei. It provides new information on the unsolved relationships between taxa that are well informed, and on the affinities of taxa that were traditionally considered as problematic, and excluded from the analysis because of the presence of many missing data.

The position of *Garnbergia* was unstable in previous analyses: it was alternatively placed within Mawsoniidae [8], [12], as the sister-group of *Libys* within Latimeriidae [8], or as the sister group of the least inclusive clade containing *Latimeria*, *Mawsonia* and *Coelacanthus* [10]. In contrast, sister-group relationship between *Garnbergia* and Latimerioidei is suggested by the present analysis. This hypothesis is supported by a single unambiguous synapomorphy: the presence of 8 to 9 fin rays on the first dorsal fin (96[1]). This synapomorphy is homoplastic and also represents an apomorphy of *Laugia*. Within clade 13, this character is well documented and in all taxa where it is known (except *Mawsonia*, *Megalocoelacanthus* and *Swenzia*) possess 8 to 9 fin rays on the first dorsal fin. Nevertheless, this character should be taken with caution: fine rays can be easily lost after the death of the animal, and the number of fine rays is variable between individuals in the extant coelacanth *Latimeria*.

Clade 13 is supported by a single unambiguous synapomorphy: the presence of denticles on the fin rays of the D1 (98[1]). This clade was recognized as Latimerioidei in previous studies. The presence of a postparietal descending process (13[1]) that was previously considered as a synapomorphy of this clade [9], [12] is here an ambiguous synapomorphy of clade 13. The ambiguity comes from the lack of information for this character in *Garnbergia* and *Rebellatrix* the most closely related taxa of latimerioids. Consequently, the presence of the descending process of the postparietal could be a synapomorphy of clade 11 [*Rebellatrix* [*Garnbergia* [Latimerioidei]]] under FAST optimization or a synapomorphy of clade 13 (Latimerioidei) under SLOW optimization.

Taxa that have been previously considered as mawsoniids (*Diplurus*, *Chinlea*, *Mawsonia*, *Axelrodichthys*, *Parnaibaia*) are here retained within clade 21. This clade is supported by four unambiguous synapomorphies: the absence of a supratemporal descending process (14[0]), the presence of an unmodified posterior end of the coronoid (56[0]), the presence of ossified ribs (92[1]), a feature unique to this clade, and the presence of differentiated scale ornaments (104[1]). The absence of the supratemporal descending process is interpreted as a secondary loss because its presence is here a synapomorphy of clade 3 (FAST optimization) or clade 5 (SLOW optimization). The unambiguous synapomorphies that support clade 21 were also supporting the clade recognised as Mawsoniidae (that is [*Diplurus* [[*Mawsonia* + *Axelrodichthys*] + [*Chinlea* + *Parnaibaia*]]]) in the unconstrained analysis of Yabumoto [14]. Additional synapomorphies included the loss of the suboperculum (32[0]) and of the subopercular branch of the mandibular sensory line canal (60[0]). However, these reversions are actually symplesiomorphies that were misinterpreted because of the lack of resolution in other latimerioids relationships. Indeed, other latimerioids that have a suboperculum (*Holophagus*, *Libys*, and *Latimeria*) and a subopercular branch of the mandibular sensory line canal (*Holophagus*, *Latimeria*, *Libys*, *Macropoma* and here *Megalocoelacanthus*) formed a polytomy with Mawsoniidae in Yabumoto's (2008) results. Consequently, the most parsimonious scenario in the phylogenetic analysis of Yabumoto [14] was favouring a reversion in Mawsoniidae instead of the retention of the plesiomorphic condition in this clade, while *Holophagus*, *Libys*, *Macropoma*, and *Latimeria* would have acquired convergently the apomorphic condition. Thanks to the better resolution the relationships of these taxa, these putative synapomorphies should be there considered as symplesiomorphies.

Within clade 21, the sister-group relationship between *Mawsonia* and *Axelrodichthys* is retained, and strongly supported by six unambiguous synapomorphies, including three non-homoplastic ones: the extrascapulars forming part of the skull roof (16[1]), the presence of a ventral process on the postorbital (41[1]), and the principal coronoid sutured to the angular (66[1]). The relationships between *Lualabaea*, *Parnaibaia*, *Chinlea* and the clade [*Mawsonia* + *Axelrodichthys*] were unresolved in the strict consensus tree from the first analysis (Figure 21) due to the instability of *Lualabaea* (Figure 21B). The first strict consensus tree proposed by Yabumoto (2008) supported a sister-group relationship between *Parnabaia* and *Chinlea* based on the presence of two homoplastic synapomorphies, the presence of anterior and posterior parietals of similar size (8[0]), and the presence of an angle on the anterior end of the lachrymojugal (36[1]). Otherwise, its second consensus tree resulting from successive weighting procedure was supporting *Parnaibaia* as the sister-group of [*Mawsonia* + *Axelrodichthys*]. Our strict consensus tree is inconsistent with these hypotheses and rather supports the sister-group relationship of *Chinlea* and [*Mawsonia* + *Axelrodichthys*] based on three homoplastic synapomorphies: the presence of coarse rugosities on the parietals and postparietals (27[2]), the presence of coarse rugosity on cheek bones (49[2]) and rugose scales (106[1]).

*Ticinepomis* branches at the base of clade 14 (Figure 22). Its position changed dramatically compared to Cloutier's hypothesis (clade F in [10]) in which it was the sister-group of a clade including *Wimania*, *Axelia*, and *Coelacanthus*. This group appears here to be polyphyletic. *Ticinepomis* was subsequently excluded by Forey [12] from the analysis because it was raising instability in the intrarelationships of the sister-group of the clade [*Coccoderma* + *Laugia*]. As pointed out by Forey [12], the irresolution resulting from the inclusion of *Ticinepomis* arose from the contradiction in the distribution of its character states rather than to the lack of data. The position of *Ticinepomis* is here supported by a single unambiguous synapomorphy: the presence of expanded median fin rays (103[1]). The same position was found in the strict consensus tree of the first analysis we performed (Figure 21), but the node was only supported by ambiguous synapomorphies. The presence of expanded median fin rays on the D1 (103[1]) is only found in *Ticinepomis*, *Libys*, and *Holophagus*. *Undina*, *Macropoma*, and *Latimeria* display unexpanded median fin rays (103[0]) and this condition is a synapomorphy of clade 18 interpreted as a reversal toward the ancestral condition of actinistians.

Additionally, clade 14 is supported by six ambiguous synapomorphies: the presence of a several median rostrals (3[1]), the presence of an anterior branch of the supratemporal commissure (22[1]), the absence of a spiracular (30[0]), the presence of a subopercular branch of the mandibular sensory line canal (60[1]), the presence of an ascending lamina of the parasphenoid (79[1]), and the presence of a ventral swelling of the palatoquadrate (110[1]).

The snout is generally a poorly preserved region of the skull in fossil actinistians [12]. Among Latimerioidei, it is only observed in *Axelrodichthys* and *Diplurus*, which possess a single rostral (3[0]), and in *Macropoma*, *Latimeria* and *Parnaibaia* which possess several median rostrals (3[1]). This condition cannot be assessed for other genera of the clade. The presence of several rostrals (3[1]) is plesiomorphic in actinistians, and lost at most in clade 3, the least inclusive clade containing *Hadronector*, *Lochmocercus* and *Latimeria*. Depending on character optimization, reversion toward the ancestral state occurs independently in *Parnaibaia* and *Latimeria* (SLOW optimization), or in *Parnaibaia* and clade 14 (FAST optimization). The latter hypothesis would support Forey's analysis [12] where the presence of several rostrals is a synapomorphy of Latimeriidae. Then, it would also suggest that *Megalocoelacanthus* possessed such a condition. Re-examination of *Libys* specimens, and better-preserved material of *Ticinepomis* and other latimeriids are needed to better understand the polarity of this character.

Among actinistians, the presence of an anterior branch of the supratemporal commissure (22[1]) over the postparietal is only observed in *Macropoma*, *Swenzia*, and *Latimeria*. The ambiguity of the polarity of this character arises from the fact that all other genera belonging to clade 14 are scored with a question mark for this character. FAST optimization supposes that the presence of the supratemporal commissure is a synapomorphy of clade 14, whereas SLOW optimization supports it as a synapomorphy of clade 19 [*Macropoma* [*Latimeria* + *Swenzia*]]. This character is unknown in *Undina* and *Ticinepomis* [12], [37], [38]. In *Megalocoelacanthus*, the posterior portion of the postparietal is covered by grooves directed anteriorly (Figures 5, 7), but the state of preservation of the skull roof does not enable to clearly identify them as anterior branches of the supratemporal commissure. In its sister-group *Libys polypterus*, the supratemporal commissure does not present anterior branches (figure 3.17 in [12]). The presence of such a feature has been considered in *Holophagus gulo* based on the presence of longitudinal grooves extending from the extrascapulars (figure 3.18 in [12]). If the presence of anterior branches of the supratemporal commissure is confirmed in *Holophagus*, our topology would imply that this feature could be a synapomorphy of clade 17.

Characters (30), (60), (79), and (110) have an ambiguous polarity within clade 14 caused by the lack of data regarding *Ticinepomis*. The presence of a spiracular (30[1]), for instance, is plesiomorphic for actinistians. The loss of the spiracular (30[0]) is convergent in clade 28 [*Laugia* + *Coccoderma*], in clade 24 [*Mawsonia* + *Axelrodichthys*], and in clade 14. A reversion to the ancestral state (30[1]) occurred in clade 20 [*Latimeria* + *Swenzia*]. The spiracular is absent in *Libys* and this condition is inferred for *Megalocoelacanthus* according to our analysis. There is a subopercular branch of the mandibular sensory line canal (60[1]) in *Megalocoelacanthus*, *Libys*, *Holophagus*, *Macropoma*, and *Latimeria*. This condition is thus a non-homoplastic synapomorphy of at least clade 15 (SLOW optimization), and is therefore inferred in *Undina* and *Swenzia*. Potentially, it could be shared by *Ticinepomis* (FAST optimization) but the available material is too poorly preserved to assess its condition [38]. The ascending lamina of the parasphenoid is originally absent in actinistians (79[0]). Its presence (79[1]) is observed in *Megalocoelacanthus*, *Undina*, *Macropoma*, *Swenzia*, and *Latimeria*, and is therefore a non-homoplastic synapomorphy of at least clade 15 (SLOW optimization). The presence of the ascending lamina of the parasphenoid is inferred in *Ticinepomis* under FAST optimization. The ventral swelling of the palatoquadrate (110) is here a newly recognised character. It is only observed in *Holophagus*, *Latimeria*, *Libys*, *Macropoma*, *Megalocoelacanthus*, and *Undina*, and unknown in *Ticinepomis* and *Swenzia*. Like previous characters, the presence of this ventrally swelling is either a synapomorphy of clade 15 (SLOW optimization) or of clade 14 (FAST optimization).

Clade 15 [[*Megalocoelacanthus* + *Libys*] + [*Holophagus* + *Undina* + [*Macropoma* [*Latimeria* + *Swenzia*]]]] is supported by a two unambiguous synapomorphies: the presence of a hook-shaped dentary (57[1]), and the presence of multiple opening for the lateral line on scales (105[1]). the presence of a hook-shaped dentary (57[1]) was previously considered as a non-homoplastic synapomorphy of the clade [*Whiteia* + Latimerioidei] in the constrained analysis of Forey [12]. This is not consistent with our analysis in which this change is convergent for clades 15, 22, and in *Whiteia*. As noted before, the relationships between *Indocoelacanthus*, *Undina*, *Holophagus*, and the clade [*Macropoma* [*Latimeria* + *Swenzia*]] were unresolved in the strict consensus tree of first analysis (Figure 21) because of the unstable position of *Indocoelacanthus* due to its incompleteness. *Undina* is more closely related to [*Macropoma* [*Latimeria* + *Swenzia*]] than *Holophagus* based on two unambiguous synapomorphies, the presence of the oral pit line removed from the center of ossification (59[1]), and the presence of unexpanded median fin rays (103[0]). This is inconsistent with previous hypotheses [9], [12], [14] that were suggesting a sister-group relationship between *Holophagus* and *Undina*. Within clade 15, the sister-group relationship between the clade [*Latimeria* + *Swenzia*] and *Macropoma* is retained in both analyses we performed (Figures 21, 22). This sister-group relationship is supported by three unambiguous synapomorphies: the presence of a preoperculum developed as a posterior tube-like canal-bearing portion and an anterior blade-like portion (39[1]), the presence of an anterodorsal excavation in the postorbital (40[1]), that is a feature exclusive to this clade, and the presence of less than eight fin rays on the first dorsal fine (96[2]). Additionally, this clade is supported by two ambiguous synapomorphies. The presence of snout bones consolidated (2[1]) is a synapomorphy of clade 19 under FAST optimization that is subsequently lost in *Latimeria*, or is a convergence in *Macropoma* and *Swenzia* under SLOW optimization. The presence of a splenial without ornament (64[0]) is alternatively a synapomorphy of clade 19 (FAST optimization), or a synapomorphy of clade 17 that is lost by convergence in *Holophagus* and *Undina*. The sister-taxon relationship between *Latimeria* and *Swenzia* is supported by two unambiguous synapomorphies: the absence of pit lines making postparietals (26[1]), and the presence of a spiracular (30[1]). These synapomorphies where also supporting this clade in the phylogenetic analysis of Clément [8] and both represent a reversal towards the ancestral condition in actinistians.

### 3. Systematic paleontology

Based on the topology proposed by Cloutier [10], Schultze [15] erected the suborder Latimerioidei (node 15 in [10]) comprising the family Mawsoniidae Schultze, 1993 (node I in [10]) and the family Latimeriidae Berg, 1940 (node 16 in [10]). Although diagnoses for each taxa cited above were given by Berg [39] and Forey [12], these taxa remain undefined. The confusion between taxon definition and taxon diagnosis has been discussed by Ghiselin [40] and Rowe [41], who emphasized the difference between these two expressions.

Phylogenetic taxonomy aims to formulate taxonomic definitions based on the phylogenetic relationships and to make a clear distinction between taxonomic diagnosis and taxonomic definition [42]–[46]. Three classes of definitions have been initially stated in phylogenetic taxonomy: apomorphy-based, stem-based, and node-based definitions [42], [47]. Apomorphy-based definition makes reference to characters, and defines the membership of a taxon as the clade stemming from the “first ancestor with a particular synapomorphy” [42], [43], [45], [46]. Apomorphy-based definitions are not used here because they encounter three problems: character ambiguity, variation in characters optimization, and homoplasy [46].

A node-based definition specifies the membership of a taxon by “the least inclusive clade that contains at least two internal specifiers”, while a stem-based definition specifies the membership of a taxon by “the most inclusive clade that contains at least one internal specifier” [46]. Stem-based and node-based definitions thus rely on the use of a definitional component termed as specifiers, i.e species or specimens stated in the phylogenetic definition as a reference point. Both definitional types have at least one internal specifier (anchor within the ingroup defined), but stem-based definition contains optionally an external specifier to define the exclusion group. An optional definitional component termed as “qualifier” can also be used to provide conditions on the clade membership. Qualifiers can be species, specimens, or features of species or specimens.

We propose here phylogenetic definitions to the taxa Latimeriidae, Mawsoniidae and Latimerioidei. The genera *Latimeria* Smith 1938 and *Mawsonia* Woodward 1907 are given as reference taxa within Latimerioidei because they are well-known and complete, deeply nested, and their position is stable in the successive topologies that have been proposed up to now [8]–[14], [28]. We thus consider *Latimeria chalumnae* and *Mawsonia gigas* reliable enough to preserve the taxonomic content of the taxa we define here.

Our study supports the presence of two major clades of coelacanths (clade 14 and 21) within a more inclusive clade (clade 13) that are retained in the strict consensus trees of both analyses performed (Figures 21, 22). However, their inter- and intra-relationships are still weakly supported. Indeed clade 14 is supported by a single unambiguous synapomorphy in the second analysis (Figure 22), and only by ambiguous synapomorphies in the first analysis we performed (Figure 21), raising a polytomy between *Ticinepomis*, clade 21 and clade 15 when branches with minimum length of zero are collapsed. Consequently, we decided to give a stem-based definition of Latimeriidae and Mawsoniidae. Moreover, it is worth noting that the family Mawsoniidae as coined by Schultze [15] is a *nomen nudum*. Actually, the name is invalid in regard with the Article 13.1 of the ICZN [48], because neither diagnostic characters nor definitions are stated in the publication. A diagnosis was subsequently given by Forey [12], but no definition has been stated since the name was coined. Based on previous topology and ours, we propose the following definitions:

Latimeriidae Berg, 1940: the most inclusive clade containing *Latimeria chalumnae* Smith, 1938 but excluding *Mawsonia gigas* Woodward, 1907.

Mawsoniidae Schultze, 1993: the most inclusive clade containing *Mawsonia gigas* Woodward, 1907 but excluding *Latimeria chalumnae* Smith, 1938.

The dichotomy between Latimeriidae and Mawsoniidae is traditionally recognized in the phylogenies, stating the relationship “Latimerioidei  =  Latimeriidae + Mawsoniidae”. In order to handle the taxa Latimerioidei in the case of relocation of species, clades, or changes in the apomorphies, we use a node-stem triplet (NST). The NST is the only phylogenetic definition that preserves the taxonomic content at a dichotomy by combining a node-based definition of a taxon with two stem-based definitions of more inclusive taxa [49]. Criteria of diversity, morphology and tradition are recommended for establishing a NST for a certain taxa [49]. With the NST the traditional equivalence statement “Latimerioidei  =  Latimeriidae + Mawsoniidae” is anchored and will always be stable in the case of a taxon branching basally to the clade 14 or to clade 21. The NTS we propose consists in a node-based definition for Latimerioidei composed of two stem-based taxa, Latimeriidae and Mawsoniidae. Using *Latimeria chalumnae* and *Mawsonia gigas* as nested specifiers, the phylogenetic definition of Latimerioidei is proposed as follows:

Latimerioidei Schultze, 1993: the least inclusive clade containing *Latimeria chalumnae* Smith, 1938 and *Mawsonia gigas* Woodward, 1907;

Latimeriidae Berg, 1940: the most inclusive clade containing *Latimeria chalumnae* Smith, 1938 but excluding *Mawsonia gigas* Woodward, 1907;

Mawsoniidae Schultze, 1993: the most inclusive clade continaining *Mawsonia gigas* Woodward, 1907 but excluding *Latimeria chalumnae* Smith, 1938.

*Megalocoelacanthus* was previously included in the family Coelacanthidae Agassiz, 1844 by Schwimmer *et* *al*. [1]. However, the family Coelacanthidae as defined by Agassiz [50] should not be longer recognized. It is actually inconsistent with the topology obtained here and in previous studies because it does not represent a monophyletic group [8]–[14], [28]. Based on the hypothesis of relatedness obtained here and using the new definitions we stated, we thus propose the following taxonomy for *Megalocoelacanthus* as well as a new diagnosis:

**OSTEICHTHYES** Huxley, 1880 [51].

**SARCOPTERYGII** Romer, 1955 [52].

**ACTINISTIA** Cope, 1891 [53].

**LATIMERIOIDEI** Schultze, 1993 [15] new definition.

**LATIMERIIDAE** Berg, 1940 [49] new definition.

***Megalocoelacanthus dobiei*** Schwimmer, Stewart & Williams, 1994.

#### Holotype

CCK-8-2-1, basisphenoid, left lower jaw, right and left palatoquadrates, pectoral girdles, left opercular, zygal plate, many branchial elements, dorsal fin spine, many indeterminate bones.

#### Paratype

AUMP 3834, right mandible, right principal coronoid, right and left palatoquadrates and isolated metapterygoids, right and left autopalatines, right gular plate, left opercular, right certohyal, single indeterminate branchial.

#### Referred material

FMNH P27534, palatoquadrate; CCK 93-6-1, and AUMP 3944, distal quadrate fragments; CCK 93-13-1, right angular fragment; AMNH 6643, principal coronoid fragment; AMNH FF 20267, ethmosphenoid and otoccipital portion of the skull, isolated snout, right and left lower jaws, isolated right and left articulars, right and left palatoquadrates, right and left autopalatines, right and left gular plates, urohyal, basibranchial associated with tooth plates, undetermined branchial arches, ceratohyal, left symplectic, right and left operculars, shoulder girdle (right and left cleithrums, clavicles, scapulocoracoids), isolated indeterminate elements.

#### Diagnosis (revised)

Parietonasal shield narrow, and longer than the posparietal shield. Supraorbital sensory line canal opening through a large groove crossed by slender pillars. Ventral descending processes present on the supratemporal and on the parietal. Basisphenoid very deep dorsoventrally. Palatoquadrate deeper than long, with distinct swelling extending from the ventral pterygoid margin immediately anterior to the quadrate. Mandibles relatively elongate posterior to the articular; articular sutured to the angular. Medial surfaces of the prearticular, palatoquadrate, and coronoid covered with tubercular shagreen. No marginal teeth on the mandible. Coronoid large, with subcircular ventral margin. Lateral surface of angular bears large pores of the sensory line canal and very faint longitudinal grooves on its posterior portion. Gular and operculum lack external ornamentation. Operculum subrhomboidal, with sharply angled anteroventral margin. Gular plates diverge strongly along posterior midline.

## Discussion and Conclusions

Since its first description, *Megalocoelacanthus* was previously related to the latimeriids *Latimeria* and *Macropoma* based on few meristic data [1]. Our phylogenetic analysis of *Megalocoelacanthus* supports the sister-group relationship with *Libys*, an Upper Jurassic genus from Bavaria, Germany. Although it is significantly smaller in size, *Libys* shares many features with *Megalocoelacanthus*. Notably, this genus also possesses a supraorbital sensory line canal that opens through a large, continuous groove stretched by pillars on either side of the parietonasal shield. When specimens of *Libys* are observed under binocular microscope (Dutel pers. obs. on BSPG XIV 501b, BSPG 1870 XIV 502, BMNH P3337), one can distinguish a suture between the base of adjacent pillars. It is thus probable that the same condition is present in *Megalocoelacanthus*, but because of the poor preservation, the suture between the supraorbitals cannot be seen. Despite the size difference, the lower jaw of *Megalocoelacanthus* and *Libys* are virtually identical, i.e. a slender and elongated mandible opened by large pores for the mandibular sensory line canal. These two genera also share the absence of a suture between the parasphenoid and the basisphenoid, which suggests fusion of the two bones, the presence of a narrow and unornamented parietonasal shield, and the palatoquadrate being very deep and short in length with a ventral swelling on the palate. Very few elements from the postcranial skeleton are present on the specimen AMNH FF 20267. However, the pectoral girdle which is preserved is virtually identical in shape and proportion in *Megalocoelacanthus* and *Libys*: the cleithrum is slender and elongated, and the clavicle is relatively very small compared to the whole pectoral girdle.

A very interesting aspect of *Megalocoelacanthus* lies in the shape and the proportion of its skull. In genera such as *Axelrodichthys* and *Mawsonia* where the skull is shallow and elongated, the basisphenoid has a short dorsum sellae, with laterally well-expanded wings, as well as a palatoquadrate longer than high [5]. In contrast, the well preserved basisphenoid of the holotype specimen CCK 88-2-1 is deeper and narrower than in these genera. The palatoquadrate of *Megalocoelacanthus* is deeper than long, and appears to be proportionally shorter in length than those of the latimeriids *Latimeria*, *Macropoma*, *Holophagus* and of the mawsoniids *Mawsonia*, *Axelrodichthys* and *Parnaibaia*. The shoulder girdle also appears to be much deeper and more slender than that of the mawsoniids *Diplurus*, *Mawsonia* and *Axelrodichthys*. *Megalocoelacanthus* clearly shows features of Latimeriidae, but the proportions of the elements of the skull and shoulder girdle are closer to what can be observed in *Libys* than in other genera of this clade.

In all the latimerioids examined, the parietals are much wider than the supraorbitals and the parietonasal shield thus represents most of the width of skull roof. *Megalocoelacanthus* and *Libys* are also very unique in that their parietonasal shield is considerably narrower compared to other coelacanths, and the skull is mainly roofed by the supraorbitals that are lying alongside the parietonasal shield. However, we cannot determine whether the bony surface within the vacuities of the supraorbital sensory line canal is a lateral extension of the parietonasal shield that is overlapped by the supratemporal. Although the skull of AMNH FF 20267 is strongly flattened laterally, the skull roof was most probably well-vaulted. In any case, it is clear that the top of the skull was much narrower than the buccal floor. When the jaws and the gular plates of AMNH FF 20267 are assembled, the minimum width of the buccal floor is much wider than the skull roof width. Taken together, these elements enable us to depict *Megalocoelacanthus* as a large-sized coelacanth with a short, laterally compressed and very deep skull, with a bell-like shape in transverse section. This is thus quite different from the more ovoid transverse section of the skull of the extant coelacanth *Latimeria chalumnae*.

Coelacanths have been depicted as a conservative group that experienced little anatomical change during their evolution. However, this widespread idea has been challenged at several times by the discovery of Paleozoic coelacanths such as *Allenypterus* [17], [54], *Holopterygius* [13] and *Miguashaia* [21], whose morphologies differ significantly from that of *Latimeria*. It now appears that coelacanths experienced a wide range of morphologies and ecologies very early in their evolutionary history. Based on geometric morphometric analysis Friedman & Coates [13] showed that the highest morphological disparity in coelacanths was reached by the Middle Devonian, but dropped in post-Carboniferous forms despite a significant increase in the taxonomic diversity. Indeed, Mesozoic coelacanths actually fit to the “*Latimeria* bodyplan” and anatomical variations in Mesozoic coelacanths seem to lie in variations of proportion of the skeletal elements rather than in radical morphological shifts.

The evolution of Mesozoic coelacanths also appears to now be marked by a significant increase in body size in at least two lineages during the Cretaceous. Nevertheless, the skull morphology of these forms is far from being homogenous and clearly falls into two morphotypes: long, shallow, wide skulls in the mawsoniids *Mawsonia* and *Axelrodichthys* and short, deep, narrow skulls in the latimeriids such as in *Latimeria*, *Macropoma*, which is amplified in *Libys* and *Megalocoelacanthus*. It is worth noting that these variations may focus considerable interest in the intracranial joint kinetic, and thereby in the feeding strategy of these large-sized taxa. Based on a better understanding of the intracranial joint kinetic in *Latimeria*, this question will deserve further investigation in the future.

The paleoenvironments represented by the occurrences of *Megalocoelacanthus* differ widely. The marine chalks of western Kansas and western Alabama represent offshore, deep-water marine environments, whereas the detrital sediments from eastern Alabama, Georgia and New Jersey (including the holotype occurrence) represent near shore, shallow marginal marine to estuarine environments. Given the range of occurrences, this suggests that *Megalocoelacanthus* was eurytopic and favored both marine and brackish waters. The records of *Megalocoelacanthus* [55] collectively suggest that it was a fairly common fish, but it is not frequently recognized in fossil assemblages and its distribution may still be underestimated. Further study will have to be carried on in order to determine the potential ecological niches that these common large, toothless, coelacanths were occupying in the Western Interior Seaway during the Late Cretaceous.

By comparison with *Libys* and *Latimeria*, the length of AMNH FF 20267 is estimated to range between 2.30 m and 3 m. However, a large isolated principal coronoid found near the holotype site in eastern Alabama extrapolates the maximum length of *Megalocoelacanthus* to ∼4.5 m [56]. Similar dimensions were previously known only through the genus *Mawsonia* from the Lower Cretaceous of Brazil, Morocco and Niger, which largest specimens are estimated to range between 3.5 and 6.3 m [2], [57], [58], and the poorly known genus *Trachymetopon* from the lower Toarcian of the Posidonia Shale of Germany [59]. *Mawsonia* was certainly non-marine and restricted to continental and estuarine environments [2], [60], whereas *Trachymetopon* was clearly marine. Although the phylogenetic position of *Trachymetopon* has still to be elucidated, our study suggests that large coelacanths evolved in at least two different lineages during the Mesozoic, in both marine and non-marine environments.

## Supporting Information

Information S1 **Character list.**
(DOC)

Information S2 **Data matrix used in the phylogenetic analysis.**
(DOC)

Information S3 **Nexus file of the first phylogenetic analysis.**
(TXT)

Information S4 **Nexus file of the second phylogenetic analysis.**
(TXT)

Information S5 **Diagnostic information for the node and terminal taxa of the strict consensus tree illustrated** **Figure 22** **.**
(DOC)
