# Correction: The Giant Cretaceous Coelacanth (Actinistia, Sarcopterygii) *Megalocoelacanthus dobiei* Schwimmer, Stewart & Williams, 1994, and Its Bearing on Latimerioidei Interrelationships

**DOI:** 10.1371/annotation/269db7d0-3c0c-478a-b44f-0aec12d0c343

**Published:** 2013-06-25

**Authors:** Hugo Dutel, John G. Maisey, David R. Schwimmer, Philippe Janvier, Marc Herbin, Gaël Clément

Author Gaël Clément is associated with the wrong affiliation. The following is the correct affiliation:

1 UMR 7207 CNRS-MNHN, Centre de Recherches sur la Paléobiodiversité et les Paléoenvironnements (CR2P), Département Histoire de la Terre, Muséum national d'Histoire naturelle, Paris, France 

