# Supplementary material for: The Giant Cretaceous Coelacanth (Actinistia, Sarcopterygii) Megalocoelacanthus dobiei Schwimmer, Stewart & Williams, 1994, and Its Bearing on Latimerioidei Interrelationships
Source: PLoS One. 2012 Nov 27;7(11):e49911. doi: 10.1371/journal.pone.0049911 (PMC3507921; doi:10.1371/journal.pone.0049911)
Supplement: Information S1 — Character list. (DOC) [file pone.0049911.s001.doc]

# Supporting information 1: Character list

**Characters taken from Forey (1998)**

Characters in blot are those that have been modified from the original list of characters published by Forey (1998).

1. Intracranial joint margin straight (0), strongly interdigitate (1).
2. Snout bones lying free from one another (0), snout bones consolidated (1).
3. Single median rostral (0), several median rostrals (internasals) (1).
4. Paired premaxillae (0), fragmented premaxillae (1).
5. Premaxilla with dorsal lamina (0), without dorsal lamina (1).
6. Anterior opening of the rostral organ contained within premaxilla (0), within separated rostral ossicle (1).
7. One pair of parietal (1), two pairs (2).
8. Anterior and posterior pairs of parietals of similar size (0), dissimilar size (1).
9. Number of supraorbitals/tectals; fewer then eight (0), more than 10 (1).
10. Preorbital absent (0), present (1).
11. Parietal descending process absent (0), present (1).
12. Intertemporal absent (0), present (1).
13. Postparietal descending process absent (0), present (1).
14. Supratemporal descending process absent (0), present (1).
15. Extrascapulars sutured with posparietals (0), free (1).
16. Extrascapulars behind level of neurocranium (0), forming part of the skull roof (1).
17. Number of extrascapulars: three (0), five (1), more than seven (2).
18. Posterior margin of the skull roof straight (0), embayed (1).
19. Supraorbital sensory canal running through centre of ossification (0), following sutural course (1).
20. Medial branch of otic canal absent (0), present (1).
21. Otic canal joining supratemporal canal within lateral extrascapular (0), in supratemporal (1).
22. Anterior branches of supratemporal commissure absent (0), present (1).
23. **Supraorbital sensory canals opening through bones as single large pores (0), bifurcating pores (1), many tiny pores (2), a large, continuous groove crossed by pillars (3).**

Libys *was coded 0 as well as* Rhabdoderma, Caridosuctor, Allenypterus, Lochmocercus, Whiteia, Laugia, Diplurus, *and* Latimeria *in Forey (1998). We here consider that the condition observed in* Libys *and* Megalocoelacanthus *(i.e. a sensory canal opening through bone as a large and continuous groove crossed by pillars) is different from that of these genera, and need to be coded as a novel state of character (3).*

1. Anterior pit line absent (0), present (1).
2. Middle and posterior pit lines within posterior half of postparietals (0), within anterior third (1).
3. Pit lines making postparietals (0), not making postparietals (1).
4. Parietals and postparietals ornamented with enamel-capped ridges/tubercles (0), bones unornamented (1), bones marked by coarse rugosities (2).
5. Parietals and postparietals without raised areas (0), with raised areas (1).
6. Cheek bones sutured to one another (0), separated from one another (1).
7. Spiracular (postspiracular) absent (0), present (1).
8. Preoperculum absent (0), present (1).
9. Suboperculum absent (0), present (1).
10. Quadratojugal absent (0), present (1).
11. Squamosal limited to the mid-level of cheek (0), extending behind the postorbital to reach the skull roof (1).
12. Lachrymojugal not expanded anteriorly (0), expanded anteriorly (1).
13. Lachrymojugal ending without anterior angle (0), angled anteriorly (1).
14. Squamosal large (0), reduced to a narrow tube surrounding the jugal sensory canal only (1).
15. Preoperculum large (0), reduced to a narrow tube surrounding the preopercular canal only (1).
16. Preoperculum undifferentiated (0), developed as a posterior tube-like canal-bearing portion and an anterior blade-like portion (1).
17. Postorbital simple, without anterodorsal excavation (0), anterodorsal excavation in the postorbital (1).
18. Postorbital without anterior process (0), with anterior process (1).
19. Postorbital large (0), reduced to a narrow tube surrounding the sensory canal only (1).
20. Postorbital entirely behind the level of the intracranial joint (0), spanning the intracranial joint (1).
21. Infraorbital canal within the postorbital, with simple pores opening directly from the main canal (0), anterior and posterior branches with the postorbital (1).
22. Infraorbital sensory canal running through centre of postorbital (0), running at the anterior margin of the postorbital (1). *Taxa coded (1) for the character 42 will automatically have a (1) here*.
23. Jugal sensory canal simple (0), with prominent branches (1).
24. Jugal canal running through centre of bone (0), running along the ventral margin of the squamosal (1).
25. Pit lines marking cheek bones (0), failing to mark cheek bones (1).
26. Ornaments upon cheek bones absent (0), tubercular (1), represented as a coarse superficial rugosity (2).
27. **Infraorbital, jugal and preopercular sensory canals opening through many tiny pores (0), opening through a few large pores (1),** **a large, continuous groove crossed by pillars (2).**

*Libys was coded “1” in Forey (1998). We here consider that the condition observed in* Libys *and* Megalocoelacanthus *is different from that of other coelacanth genera, and need to be coded as a novel state of character (2).*

1. Lachrymojugal sutured to preorbital and lateral rostral (0), lying in a sutural contact with the tectal-supraorbital series (1).
2. Sclerotic ossicles absent (0), present (1).
3. Retroarticular and articular co-ossified (0), separated (1).
4. Dentary teeth fused to the dentary (0), separated from dentary (1).
5. Number of coronoids, coded as integers.
6. Coronoid opposite to the posterior end of dentary not modified (0), modified (1).
7. Dentary simple (0), dentary hook-shaped (1).
8. Oral pit line confined to angular (0), oral pit line reaching forward to the dentary and/or the splenial (1)
9. Oral pit line located at centre of ossification of angular (0), removed from centre of ossification (1).
10. Subopercular branch of the mandibular sensory canal absent (0), present (1).
11. Dentary sensory pore absent (0), present (1).
12. Ridged (0) or granular ornaments (1).
13. Dentary with ornament (0), without ornament (1).
14. Splenial with ornament (0), without ornament (1).
15. Dentary without prominent lateral swelling (0), with swelling (1).
16. Principal coronoid lying free (0), sutured to angular (1).
17. Coronoid fangs absent (0), present (1).
18. Prearticular and/or coronoid teeth pointed and smooth (0), rounded and marked with fine striations radiating from the crown (1).
19. Orbitosphenoid and basisphenoid regions co-ossified (0), separate (1).
20. Basisphenoid extending forward to enclose the optic foramen (0), optic foramen lying within separate interorbital ossification or cartilage (1).
21. Processus connectens meeting parasphenoid (0), failing to meet parasphenoid (1).
22. Basipterygoid process absent (0), present (1).
23. Antotic process not covered by parietal descending process (0), covered (1).
24. Temporal excavation lined with bone (1), not lined (0).
25. Otico-occipital solid (0), separated to prootic/opisthotic (1).
26. Supraoccipital absent (0), present (1).
27. Vestibular fontanelle absent (0), present (1).
28. Buccohypophysial canal closed (0), opening through parasphenoid (1).
29. Parasphenoid without ascending laminae anteriorly (0), ascending laminae (1).
30. Suprapteygoid process absent (0), present (1).
31. Vomers not meeting in the midline (0), meeting medially (1).
32. Prootic without complex suture with the basioccipital (0), with a complex suture (1).
33. Superficial ophthalmic branch of anterodorsal lateral line nerve not piercing antotic process (0), piercing antotic process (1).
34. Process on braincase for articulation of infrabranchial 1 absent (0), present (1).
35. Separate lateral ethmoids absent (0), present (1).
36. Separate basioccipital absent (0), present (1).
37. Dorsum sellae small (0), large and constricting entrance to cranial cavity anterior to the intracranial joint (1).
38. Extracleithrum absent (0), present (1).
39. Anocleithrum simple (0), forked (1).
40. Posterior neural and haemal spines abutting one another (0), not abutting (1).
41. Occipital neural arches not expanded (0), expanded (1).
42. Ossified ribs absent (0), present (1).
43. Diphycercal tail absent (0), present (1).
44. Fin rays more numerous than radials (0), equal in number (1).
45. Fin ray branched (0), unbranched (1).
46. Fin rays in D1 > 10 = (0), 8-9 = (1), < 8 = (2).
47. Caudal lobes symmetrical (0), asymmetrical (1).
48. D1 without denticles (0), with denticles (1).
49. Paired fin rays not expanded (0), expanded (1).
50. Pelvics abdominal (0), thoracic (1).
51. Basal plate of D1 with smooth ventral margin (0), emarginated and accommodating the tips of adjacent neural spines (1).
52. D2 basal support simple (0), forked anteriorly (1).
53. Median fin rays not expanded (0), expanded (1).
54. Scale ornament not differentiated (0), differentiated (1).
55. Lateral line openings in scales single (0), multiple (1).
56. Scales ornament of ridges or tubercles (0), rugose (1).
57. **Ossified bladder absent (0), present (1).**
58. Pelvic bones of each side remain separate (0), pelvic bones of either side fused in midline (1).

**Character taken from Friedman & Coates (2006)**

1. Ventral keel scales absent (0), present (1).

**Newly recognized characters**

1. Ventral swelling of the palatoquadrate absent (0), present (1).
