# Supplementary material for: The Giant Cretaceous Coelacanth (Actinistia, Sarcopterygii) Megalocoelacanthus dobiei Schwimmer, Stewart & Williams, 1994, and Its Bearing on Latimerioidei Interrelationships
Source: PLoS One. 2012 Nov 27;7(11):e49911. doi: 10.1371/journal.pone.0049911 (PMC3507921; doi:10.1371/journal.pone.0049911)
Supplement: Information S2 — Data matrix used in the phylogenetic analysis. (DOC) [file pone.0049911.s002.doc]

# Supporting information 2: Data matrix used in the phylogenetic analysis

|  | **1** | **2** | **3** | **4** | **5** | **6** | **7** | **8** | **9** | **10** | **11** | **12** | **13** | **14** | **15** | **16** | **17** | **18** | **19** | **20** | **21** | **22** | **23** | **24** |
| --- | --- | --- | --- | --- | --- | --- | --- | --- | --- | --- | --- | --- | --- | --- | --- | --- | --- | --- | --- | --- | --- | --- | --- | --- |
| Actinopterygians | ? | 0 | 0 | 0 | 0 | ? | 1 | ? | ? | 0 | 0 | 1 | 0 | 0 | 0 | 0 | ? | 0 | 0 | 0 | 0 | 0 | 2 | 1 |
| Porolepiforms | 0 | 0 | 1 | 0 | 0 | ? | 1 | ? | ? | 0 | 0 | 0 | 0 | 0 | 0 | 0 | 0 | 0 | 0 | 0 | 0 | 0 | 2 | 1 |
| *Diplocercides* | 0 | 0 | 1 | ? | ? | ? | 2 | 1 | 0 | 1 | 0 | 0 | 0 | 0 | 0 | 0 | 0 | 0 | 1 | 0 | 0 | 0 | 2 | 1 |
| *Rhabdoderma* | 1 | 0 | 0 | 0 | 0 | 0 | 2 | 0 | 0 | 1 | 1 | 0 | 0 | 1 | 0 | 0 | 1 | 0 | 1 | 1 | 0 | 0 | 0 | 0 |
| *Caridosuctor* | 1 | 0 | 0 | 0 | 0 | 0 | 2 | 1 | 0 | 1 | ? | 0 | ? | ? | 0 | 0 | 1 | 0 | 1 | 1 | 0 | 0 | 0 | 0 |
| *Hadronector* | 0 | 0 | 1 | 0 | 0 | 0 | 2 | 1 | ? | 1 | ? | 0 | ? | ? | 0 | 0 | 0 | 0 | 1 | 0 | 0 | 0 | 1 | 0 |
| *Polyosteorhynchus* | 0 | 0 | ? | 0 | 0 | 0 | 2 | 1 | 0 | 1 | ? | ? | ? | 1 | 0 | 0 | ? | 0 | 1 | 0 | 1 | 0 | 1 | ? |
| *Allenypterus* | 0 | ? | ? | ? | ? | ? | 2 | 0 | 0 | 1 | ? | 0 | ? | ? | 0 | 0 | 0 | 0 | 1 | 0 | 0 | 0 | 0 | 0 |
| *Lochmocercus* | ? | ? | ? | ? | ? | ? | ? | ? | ? | ? | ? | ? | ? | ? | ? | ? | ? | ? | 1 | 0 | 1 | 0 | 0 | ? |
| *Coelacanthus* | 0 | 0 | ? | 1 | 1 | ? | 2 | 1 | 0 | 0 | 1 | 0 | 0 | 1 | 0 | 0 | 1 | 1 | ? | 1 | 1 | 0 | ? | 0 |
| *Spermatodus* | 1 | 0 | 0 | 0 | 0 | 0 | 2 | 1 | 0 | 1 | 1 | 0 | 0 | 1 | 1 | 0 | ? | 1 | 1 | 1 | ? | 0 | 2 | 0 |
| *Whiteia* | 0 | 0 | ? | 0 | 0 | 0 | 2 | 0 | 0 | 1 | 1 | 0 | 0 | 1 | 1 | 0 | 1 | 1 | 1 | 1 | 1 | 0 | 0 | 0 |
| *Laugia* | 1 | 1 | ? | 0 | ? | ? | 1 | ? | 0 | 1 | 1 | 0 | 0 | 1 | 0 | 0 | 1 | 0 | 1 | 1 | 0 | 0 | 0 | 0 |
| *Sassenia* | 1 | 0 | ? | ? | ? | ? | 2 | ? | 0 | 1 | 1 | 0 | 0 | 1 | 0 | 0 | ? | 0 | 1 | ? | ? | 0 | 2 | 0 |
| *Chinlea* | 0 | 0 | ? | 0 | 1 | ? | 2 | 0 | 0 | 0 | 1 | 0 | ? | ? | 0 | 0 | 1 | 1 | ? | 1 | 1 | 0 | ? | ? |
| *Diplurus* | 0 | 0 | 0 | 0 | 1 | ? | 2 | 1 | 1 | 0 | 1 | 0 | 1 | 0 | 1 | 0 | 2 | 1 | 1 | 1 | 1 | 0 | 0 | 0 |
| *Holophagus* | 1 | 0 | ? | 0 | 1 | ? | 2 | 1 | 1 | 0 | 1 | 0 | 1 | 1 | 1 | 0 | ? | 1 | 1 | 1 | 1 | ? | 2 | 0 |
| *Undina* | 0 | 0 | ? | 0 | 0 | ? | 2 | 1 | 1 | 0 | 1 | 0 | 1 | 1 | 1 | 0 | 2 | 1 | 1 | ? | 1 | ? | 2 | ? |
| *Coccoderma* | 1 | 0 | ? | 0 | 1 | 1 | 2 | 1 | 0 | ? | 1 | 0 | 0 | 1 | 0 | 0 | 1 | 0 | 1 | 1 | 0 | 0 | 2 | 0 |
| *Libys* | 0 | ? | ? | ? | 1 | ? | ? | ? | ? | ? | 1 | 0 | 1 | 1 | 1 | ? | ? | 1 | 1 | 1 | 1 | ? | 3 | 0 |
| *Mawsonia* | 1 | ? | ? | ? | ? | ? | 2 | 1 | 0 | 0 | 1 | 0 | 1 | 0 | 0 | 1 | 1 | 1 | ? | 1 | 1 | 0 | 2 | 0 |
| *Macropoma* | 0 | 1 | ? | 0 | ? | 1 | 2 | 1 | 1 | 0 | 1 | 0 | 1 | 1 | 1 | 0 | 2 | 1 | 1 | 1 | 1 | 1 | 2 | 0 |
| *Latimeria* | 0 | 0 | 1 | 1 | 1 | 1 | 2 | 1 | 1 | 0 | 1 | 0 | 1 | 1 | 1 | 0 | 2 | 1 | 1 | 1 | 1 | 1 | 0 | 0 |
| *Miguashaia* | 0 | 0 | ? | 0 | 0 | 0 | 1 | ? | 0 | ? | 0 | 1 | ? | ? | 0 | 0 | 0 | 0 | 0 | 0 | 0 | 0 | 2 | ? |
| *Axelrodichthys* | 1 | 0 | 0 | 0 | 1 | 1 | 2 | 1 | 0 | 0 | 1 | 0 | 1 | 0 | 0 | 1 | 1 | 1 | 1 | ? | 1 | 0 | 2 | 0 |
| *Holopterygius* | ? | 0 | ? | 0 | ? | ? | ? | ? | ? | ? | ? | ? | ? | ? | ? | ? | ? | ? | ? | ? | ? | ? | ? | ? |
| *Garnbergia* | ? | ? | ? | ? | ? | ? | 2 | 1 | ? | 0 | ? | 0 | ? | ? | ? | ? | ? | 1 | ? | ? | ? | ? | ? | ? |
| *Parnaibaia* | 0 | 1 | 1 | 1 | ? | ? | 2 | 0 | 0 | 0 | ? | 0 | ? | ? | 0 | 0 | 1 | 1 | ? | 1 | ? | ? | 2 | ? |
| *Swenzia* | 0 | 1 | ? | 0 | ? | ? | 2 | ? | ? | ? | ? | 0 | 1 | 1 | ? | ? | ? | 1 | ? | ? | 1 | 1 | ? | 0 |
| *Guizhoucoelacanthus* | 0 | 0 | 0 | 0 | ? | ? | 1 | 1 | 0 | 1 | ? | 0 | ? | ? | 0 | 0 | 0 | 1 | 1 | 1 | 1 | 0 | 0 | 0 |
| *Piveteauia* | ? | ? | ? | ? | ? | ? | ? | ? | ? | ? | 1 | ? | 1 | ? | 1 | 0 | ? | 1 | ? | 1 | ? | ? | ? | 0 |
| *Euporosteus* | ? | ? | ? | ? | ? | 0 | ? | 1 | ? | ? | ? | 2 | 0 | ? | 1 | 0 | ? | ? | ? | ? | ? | ? | ? | ? |
| *Axelia* | ? | ? | ? | ? | ? | ? | 1 | ? | 0 | 1 | 1 | 0 | ? | 1 | 1 | 0 | 2 | 1 | 1 | ? | 1 | 0 | 0 | ? |
| *Wimania* | ? | ? | ? | ? | ? | ? | 1 | ? | ? | ? | 1 | 0 | 0 | 1 | ? | ? | ? | ? | ? | ? | ? | ? | ? | ? |
| *Ticinepomis* | 0 | ? | ? | 0 | ? | ? | ? | ? | ? | ? | ? | 0 | ? | ? | 1 | ? | ? | 1 | ? | ? | ? | ? | ? | ? |
| *Rebellatrix* | ? | ? | ? | ? | ? | ? | ? | ? | ? | ? | ? | ? | ? | ? | ? | ? | ? | ? | ? | ? | ? | ? | ? | ? |
| *Megalocoelacanthus* | 0 | 1 | ? | 0 | 0 | 1 | 2 | 1 | 0 | 0 | 1 | 0 | 1 | 1 | 1 | 0 | ? | 1 | 1 | 1 | 1 | ? | 3 | 0 |

|  | **25** | **26** | **27** | **28** | **29** | **30** | **31** | **32** | **33** | **34** | **35** | **36** | **37** | **38** | **39** | **40** | **41** | **42** | **43** | **44** | **45** |
| --- | --- | --- | --- | --- | --- | --- | --- | --- | --- | --- | --- | --- | --- | --- | --- | --- | --- | --- | --- | --- | --- |
| Actinopterygians | 1 | ? | 0 | 0 | 0 | 0 | 1 | 1 | 1 | 1 | ? | ? | ? | 0 | 0 | ? | ? | ? | ? | ? | ? |
| Porolepiforms | 0 | ? | 0 | 0 | 0 | 1 | 1 | 1 | 1 | 1 | ? | ? | 0 | 0 | 0 | 0 | 0 | 0 | 0 | 0 | 0 |
| *Diplocercides* | 1 | 0 | 0 | 0 | 0 | 1 | 1 | 1 | 0 | 1 | 0 | 0 | 0 | 0 | 0 | 0 | 0 | 0 | 0 | 0 | 0 |
| *Rhabdoderma* | 1 | 0 | 0 | 0 | 0 | 1 | 1 | 1 | 0 | 0 | 0 | 0 | 0 | 0 | 0 | 0 | 0 | 0 | 0 | 0 | 1 |
| *Caridosuctor* | 1 | 0 | 0 | 0 | 0 | 1 | 1 | 1 | ? | ? | 0 | 0 | 0 | 0 | 0 | 0 | 0 | 0 | 0 | ? | ? |
| *Hadronector* | 1 | 0 | 0 | 0 | 0 | 1 | 1 | 1 | 0 | 0 | ? | ? | 0 | 0 | 0 | 0 | 0 | 0 | 0 | ? | 1 |
| *Polyosteorhynchus* | ? | ? | 0 | 0 | 0 | 1 | 1 | 1 | ? | 0 | 0 | 0 | 0 | 0 | 0 | 0 | 0 | 0 | 0 | ? | 1 |
| *Allenypterus* | 0 | 0 | 0 | 0 | 1 | ? | 1 | 1 | 0 | 0 | 0 | 0 | 0 | 0 | 0 | 0 | 0 | 1 | 0 | 0 | 1 |
| *Lochmocercus* | ? | ? | 0 | 0 | 0 | 1 | 1 | 1 | ? | ? | 0 | 0 | 0 | 0 | 0 | 0 | 0 | 0 | ? | 0 | 1 |
| *Coelacanthus* | ? | 1 | 1 | 0 | 1 | ? | ? | ? | 0 | 0 | 0 | 0 | 1 | ? | ? | 0 | 0 | 1 | 0 | ? | 1 |
| *Spermatodus* | ? | 1 | 0 | 0 | 1 | ? | 1 | 1 | 0 | 1 | 0 | 0 | 0 | 0 | 0 | 0 | 0 | 0 | ? | ? | ? |
| *Whiteia* | 1 | 0 | 0 | 0 | 1 | 1 | 1 | 1 | 0 | 0 | 0 | 1 | 0 | 0 | 0 | 0 | 0 | 0 | 0 | 1 | 1 |
| *Laugia* | ? | 1 | 0 | 0 | 1 | 0 | 0 | 0 | 0 | 0 | 0 | 0 | 0 | 0 | 0 | 0 | 0 | 0 | 0 | 1 | 0 |
| *Sassenia* | 1 | 0 | 0 | 0 | 1 | 1 | 1 | 1 | 0 | 1 | 0 | 0 | 0 | 0 | 0 | 0 | 0 | 0 | 0 | 0 | 1 |
| *Chinlea* | ? | ? | 2 | 0 | 1 | 1 | 1 | 0 | 0 | ? | 1 | 1 | 0 | 0 | 0 | 0 | 0 | 0 | 1 | ? | 0 |
| *Diplurus* | ? | 1 | 1 | 0 | 1 | 1 | 1 | 0 | 0 | 0 | 1 | 0 | 0 | 0 | 0 | 0 | 0 | 1 | 0 | 0 | 1 |
| *Holophagus* | ? | 1 | 0 | 0 | 1 | 0 | 1 | 1 | 0 | 0 | 1 | 0 | 0 | 0 | 0 | 0 | 0 | 0 | 0 | ? | 1 |
| *Undina* | ? | ? | 0 | 1 | 1 | 0 | 1 | 1 | 0 | 0 | ? | ? | 0 | 0 | 0 | 0 | 0 | 0 | 0 | ? | 1 |
| *Coccoderma* | 1 | 0 | 1 | 0 | 1 | 0 | 1 | 0 | 0 | ? | 0 | 0 | 1 | 1 | 0 | 0 | 0 | 0 | 0 | 1 | 0 |
| *Libys* | ? | 1 | 1 | 0 | 1 | 0 | 1 | 0 | 0 | 0 | 1 | 0 | 0 | 0 | 1 | 0 | 0 | 0 | 0 | ? | 1 |
| *Mawsonia* | ? | 1 | 2 | 0 | 1 | 0 | ? | 0 | 0 | 0 | 1 | 0 | 0 | 1 | 0 | 0 | 1 | 0 | 1 | ? | 0 |
| *Macropoma* | ? | 1 | 0 | 0 | 1 | 0 | 1 | ? | 0 | 0 | 1 | 0 | 0 | 0 | 1 | 1 | 0 | 0 | 0 | 1 | 1 |
| *Latimeria* | 1 | 0 | 2 | 1 | 1 | 1 | 1 | 1 | 0 | 0 | 1 | 0 | 0 | 0 | 1 | 1 | 0 | 0 | 0 | 1 | 1 |
| *Miguashaia* | 0 | 0 | 0 | 0 | 0 | ? | 1 | 1 | 0 | 1 | ? | ? | 0 | 0 | 0 | 0 | 0 | 0 | 0 | ? | 0 |
| *Axelrodichthys* | ? | 1 | 2 | 0 | 1 | 0 | 1 | 0 | 0 | 0 | 1 | 0 | 1 | 1 | 0 | 0 | 1 | 0 | 1 | 1 | 0 |
| *Holopterygius* | 0 | ? | ? | ? | ? | ? | ? | ? | ? | ? | ? | ? | ? | ? | ? | ? | ? | ? | ? | ? | ? |
| *Garnbergia* | ? | ? | ? | ? | 1 | ? | 1 | 0 | 0 | 0 | 1 | 1 | 0 | 0 | ? | 0 | 0 | 0 | 0 | ? | ? |
| *Parnaibaia* | ? | 1 | 1 | 0 | 1 | 1 | 1 | 0 | 0 | 0 | 1 | 1 | 0 | 0 | 0 | 0 | 0 | 0 | 1 | ? | ? |
| *Swenzia* | 1 | 0 | 1 | 1 | 1 | 1 | 1 | ? | 0 | 0 | 1 | 0 | 0 | 0 | 1 | 1 | 0 | 0 | 0 | 1 | 1 |
| *Guizhoucoelacanthus* | 0 | 0 | 1 | 0 | 1 | ? | 1 | 0 | 1 | 0 | 0 | 1 | 0 | 0 | 0 | 0 | 0 | 0 | ? | ? | 0 |
| *Piveteauia* | ? | ? | ? | ? | 0 | 1 | 1 | ? | ? | 0 | 0 | 0 | ? | ? | ? | ? | ? | ? | ? | ? | ? |
| *Euporosteus* | ? | ? | ? | ? | ? | ? | ? | 0 | ? | ? | ? | ? | ? | ? | ? | ? | ? | ? | ? | ? | ? |
| *Axelia* | ? | ? | 0 | 0 | ? | ? | ? | ? | ? | ? | ? | ? | ? | ? | ? | ? | ? | ? | ? | ? | ? |
| *Wimania* | ? | ? | ? | ? | 1 | ? | 0 | ? | ? | ? | 0 | 1 | 0 | 0 | 0 | ? | 0 | 0 | ? | ? | ? |
| *Ticinepomis* | ? | ? | ? | ? | 1 | ? | 1 | ? | 0 | ? | ? | ? | ? | ? | ? | 0 | 0 | 1 | 0 | ? | ? |
| *Rebellatrix* | ? | ? | ? | ? | ? | ? | ? | ? | ? | ? | ? | ? | ? | ? | ? | ? | ? | ? | ? | ? | ? |
| *Megalocoelacanthus* | ? | ? | 1 | 0 | 1 | ? | ? | ? | ? | ? | ? | ? | ? | ? | ? | ? | ? | ? | ? | ? | ? |

|  | **46** | **47** | **48** | **49** | **50** | **51** | **52** | **53** | **54** | **55** | **56** | **57** | **58** | **59** | **60** | **61** | **62** | **63** | **64** | **65** | **66** |
| --- | --- | --- | --- | --- | --- | --- | --- | --- | --- | --- | --- | --- | --- | --- | --- | --- | --- | --- | --- | --- | --- |
| Actinopterygians | ? | ? | 0 | 1 | 0 | ? | 1 | 0 | 0 | 4 | 0 | 0 | 0 | ? | 0 | 0 | 0 | 0 | 0 | 0 | 0 |
| Porolepiforms | 0 | 0 | 0 | 1 | 0 | 0 | 1 | 0 | 0 | 3 | 0 | 0 | 0 | ? | 0 | 0 | ? | 0 | 0 | 0 | 0 |
| *Diplocercides* | 0 | 0 | 0 | 1 | 0 | 0 | 1 | 0 | 0 | 3 | 0 | 0 | 1 | 0 | 0 | 0 | 0 | 0 | 0 | 0 | 0 |
| *Rhabdoderma* | 1 | 0 | 0 | 1 | 0 | 0 | 1 | 0 | 1 | 4 | 1 | 0 | 0 | 0 | 0 | 1 | 0 | 0 | 1 | 0 | 0 |
| *Caridosuctor* | 0 | 0 | 0 | 1 | 0 | 0 | 1 | 0 | 1 | 4 | 1 | 0 | 0 | 0 | 0 | 1 | 0 | 1 | 0 | 0 | 0 |
| *Hadronector* | ? | 0 | ? | 1 | 0 | 0 | 1 | 0 | ? | ? | ? | 0 | 0 | 1 | 0 | 0 | 0 | 0 | 0 | 0 | 0 |
| *Polyosteorhynchus* | ? | 0 | 0 | 1 | 0 | 0 | 1 | 0 | 1 | ? | 0 | 0 | 0 | 1 | 0 | 1 | 0 | ? | ? | 0 | 0 |
| *Allenypterus* | ? | ? | 1 | 0 | 1 | 0 | 1 | 0 | 1 | ? | ? | 0 | 1 | 1 | 0 | 0 | 0 | 1 | 0 | 0 | 0 |
| *Lochmocercus* | 0 | 0 | 0 | ? | 0 | 0 | 1 | ? | 0 | ? | 0 | 0 | ? | ? | 0 | 0 | 0 | ? | ? | 0 | 0 |
| *Coelacanthus* | ? | 1 | ? | 1 | 0 | 0 | 1 | 0 | 1 | 4 | 1 | 0 | ? | ? | 0 | 1 | ? | 1 | 1 | 0 | 0 |
| *Spermatodus* | ? | ? | 0 | 1 | 0 | 0 | 1 | 0 | 1 | 4 | 1 | ? | 0 | 0 | 0 | ? | 1 | ? | ? | ? | 0 |
| *Whiteia* | 1 | 0 | 0 | 1 | 0 | 0 | 1 | 1 | 1 | 4 | 1 | 1 | 0 | 1 | ? | 1 | 1 | 1 | 1 | 0 | 0 |
| *Laugia* | 0 | 1 | 1 | 1 | 0 | 0 | 1 | 0 | 1 | 4 | 0 | 0 | 0 | 0 | 0 | 1 | 1 | 1 | 1 | 0 | 0 |
| *Sassenia* | 0 | 0 | 0 | 1 | 0 | 0 | 1 | ? | 1 | ? | 1 | 0 | ? | ? | 0 | 1 | 1 | 1 | 1 | 0 | 0 |
| *Chinlea* | ? | ? | ? | 2 | 0 | 1 | 0 | ? | ? | ? | 1 | 1 | ? | ? | ? | 1 | ? | 1 | 1 | 0 | 0 |
| *Diplurus* | 1 | 1 | 1 | 0 | 1 | 1 | 0 | 1 | ? | ? | 0 | 0 | 0 | 0 | 0 | 1 | ? | 1 | 1 | 0 | 0 |
| *Holophagus* | ? | ? | 1 | 1 | 0 | 1 | 0 | 1 | ? | ? | 1 | 1 | 0 | 0 | 1 | 1 | 1 | 1 | 0 | 0 | 0 |
| *Undina* | 0 | 1 | 1 | 1 | 0 | 1 | 0 | 1 | ? | ? | 1 | 1 | 0 | 1 | ? | 1 | 1 | 0 | 0 | 0 | 0 |
| *Coccoderma* | 0 | 1 | 1 | 0 | 0 | 0 | 1 | ? | 1 | 4 | 1 | 0 | 0 | 0 | 0 | 1 | 1 | 1 | 1 | 0 | 0 |
| *Libys* | ? | ? | ? | 0 | 2 | ? | 0 | ? | 1 | 4 | 1 | 1 | 0 | 0 | 1 | 1 | ? | 1 | 1 | 0 | 0 |
| *Mawsonia* | 0 | 0 | 1 | 2 | 0 | 0 | 0 | 1 | 1 | ? | ? | 1 | ? | ? | 0 | 1 | ? | 1 | 1 | 1 | 1 |
| *Macropoma* | 0 | 1 | 1 | 1 | 0 | 1 | 0 | 1 | 1 | 4 | ? | 1 | 0 | 1 | 1 | 1 | 1 | 1 | 1 | 0 | 0 |
| *Latimeria* | 1 | 1 | 1 | 1 | 1 | 1 | 0 | 1 | 1 | 4 | 1 | 1 | 0 | 1 | 1 | 1 | ? | 1 | 1 | 0 | 0 |
| *Miguashaia* | 0 | 0 | ? | 1 | 0 | ? | 1 | ? | 0 | ? | ? | 0 | 0 | 1 | ? | ? | 0 | ? | ? | 0 | 0 |
| *Axelrodichthys* | 0 | 0 | 1 | 2 | 0 | 1 | 0 | 1 | 1 | 4 | 0 | 1 | ? | ? | 0 | 1 | ? | 1 | 1 | 1 | 1 |
| *Holopterygius* | ? | ? | ? | ? | ? | ? | ? | ? | ? | 0 | 0 | ? | ? | 0 | ? | ? | ? | ? | ? | ? | ? |
| *Garnbergia* | ? | ? | ? | 1 | ? | 1 | 0 | ? | ? | ? | ? | ? | ? | ? | ? | ? | ? | ? | ? | ? | ? |
| *Parnaibaia* | ? | ? | ? | 1 | ? | 1 | 0 | 1 | ? | ? | 0 | 1 | ? | ? | ? | 1 | 1 | 1 | 1 | 1 | ? |
| *Swenzia* | 0 | 1 | 1 | 1 | 0 | ? | 0 | ? | 1 | ? | ? | 1 | 0 | 1 | ? | 1 | ? | 1 | 1 | 0 | ? |
| *Guizhoucoelacanthus* | ? | 0 | 0 | 1 | 0 | 0 | 0 | ? | ? | ? | ? | 0 | 0 | 0 | ? | 1 | 1 | 1 | 1 | 0 | 0 |
| *Piveteauia* | 1 | 0 | ? | 1 | ? | ? | 1 | 0 | ? | ? | ? | 0 | ? | ? | ? | ? | ? | ? | ? | ? | 0 |
| *Euporosteus* | ? | ? | ? | ? | ? | ? | ? | ? | ? | ? | ? | ? | ? | ? | ? | ? | ? | ? | ? | ? | ? |
| *Axelia* | ? | ? | ? | ? | ? | ? | 1 | ? | ? | ? | ? | 0 | ? | ? | ? | 1 | ? | ? | ? | ? | 0 |
| *Wimania* | ? | 0 | ? | ? | ? | 0 | 1 | ? | ? | ? | 1 | 0 | ? | ? | ? | ? | ? | ? | ? | ? | ? |
| *Ticinepomis* | ? | ? | ? | ? | ? | ? | ? | ? | 1 | ? | ? | 0 | 0 | ? | ? | 1 | 1 | 1 | 1 | 0 | 0 |
| *Rebellatrix* | ? | ? | ? | 1 | ? | ? | ? | 1 | ? | ? | ? | ? | ? | 1 | ? | ? | ? | ? | ? | ? | ? |
| *Megalocoelacanthus* | ? | ? | ? | ? | 2 | ? | 0 | 1 | 1 | ? | 0 | 1 | ? | ? | 1 | 1 | ? | 1 | 1 | 0 | 0 |

|  | **67** | **68** | **69** | **70** | **71** | **72** | **73** | **74** | **75** | **76** | **77** | **78** | **79** | **80** | **81** | **82** | **83** | **84** | **85** | **86** | **87** |
| --- | --- | --- | --- | --- | --- | --- | --- | --- | --- | --- | --- | --- | --- | --- | --- | --- | --- | --- | --- | --- | --- |
| Actinopterygians | 0 | 0 | 0 | ? | ? | 1 | ? | ? | 0 | 0 | 1 | 1 | 0 | 0 | 0 | 0 | 0 | 1 | 0 | 0 | 0 |
| Porolepiforms | 1 | 0 | 0 | 1 | 0 | 1 | ? | 0 | 0 | 0 | ? | 1 | 0 | 1 | 0 | 0 | 0 | ? | 0 | ? | 0 |
| *Diplocercides* | 0 | 0 | 0 | 1 | 1 | 1 | 0 | 1 | 0 | 0 | 1 | 1 | 0 | 1 | ? | 0 | 1 | 0 | 0 | 0 | 0 |
| *Rhabdoderma* | 1 | 0 | 1 | 1 | 1 | 0 | 1 | 1 | 0 | ? | 0 | 0 | 0 | 0 | ? | 0 | 0 | 0 | 1 | 0 | 1 |
| *Caridosuctor* | 1 | ? | ? | ? | ? | ? | ? | ? | ? | ? | ? | ? | ? | ? | ? | ? | ? | ? | ? | ? | ? |
| *Hadronector* | ? | ? | ? | ? | ? | ? | ? | ? | ? | ? | ? | ? | ? | ? | ? | ? | ? | ? | ? | ? | ? |
| *Polyosteorhynchus* | 0 | ? | ? | ? | ? | ? | ? | ? | ? | ? | ? | ? | ? | ? | ? | ? | ? | ? | ? | ? | ? |
| *Allenypterus* | ? | ? | ? | ? | ? | ? | ? | ? | ? | ? | ? | ? | ? | ? | ? | ? | ? | ? | ? | ? | ? |
| *Lochmocercus* | 0 | ? | ? | ? | ? | ? | ? | ? | ? | ? | ? | ? | ? | ? | ? | ? | ? | ? | ? | ? | ? |
| *Coelacanthus* | 0 | 0 | ? | ? | ? | ? | ? | ? | ? | ? | ? | ? | ? | ? | ? | ? | ? | ? | ? | ? | ? |
| *Spermatodus* | 1 | 1 | 1 | 0 | 1 | 0 | 1 | ? | ? | 0 | 1 | 1 | 0 | ? | ? | 0 | 0 | ? | 1 | 0 | 1 |
| *Whiteia* | 0 | 0 | 1 | 0 | 0 | 0 | 1 | 1 | 1 | ? | ? | 0 | 0 | ? | 1 | 1 | 0 | 1 | 1 | 1 | 1 |
| *Laugia* | 0 | 0 | 1 | 1 | 1 | 0 | 1 | 1 | 1 | 0 | 1 | 1 | 0 | ? | ? | 0 | 0 | 0 | 1 | 0 | 1 |
| *Sassenia* | ? | ? | 0 | 1 | 1 | 0 | 1 | 1 | 0 | 0 | 1 | ? | 0 | 1 | ? | 0 | 0 | 1 | 1 | 0 | 1 |
| *Chinlea* | 1 | ? | ? | ? | ? | ? | ? | ? | ? | ? | ? | ? | ? | ? | ? | ? | ? | ? | ? | ? | ? |
| *Diplurus* | 0 | 0 | 1 | 0 | 0 | 0 | 1 | ? | 1 | 1 | ? | 0 | 0 | ? | ? | ? | 0 | ? | 1 | 1 | 1 |
| *Holophagus* | 1 | ? | ? | ? | ? | ? | ? | ? | 1 | 1 | ? | ? | ? | ? | ? | 1 | ? | ? | 1 | ? | ? |
| *Undina* | 1 | 0 | 1 | 0 | 0 | 0 | 1 | 0 | 1 | 1 | ? | 0 | 1 | ? | ? | 1 | ? | ? | 1 | 1 | 1 |
| *Coccoderma* | 0 | ? | ? | ? | ? | ? | ? | ? | ? | ? | ? | ? | ? | ? | ? | ? | ? | ? | ? | ? | ? |
| *Libys* | 0 | 1 | ? | ? | ? | ? | ? | ? | ? | ? | ? | ? | ? | ? | ? | ? | ? | ? | ? | ? | ? |
| *Mawsonia* | 0 | 1 | ? | ? | ? | ? | ? | ? | ? | ? | ? | ? | ? | ? | ? | ? | ? | ? | ? | ? | ? |
| *Macropoma* | 0 | 0 | 1 | 0 | 0 | 0 | 1 | 0 | 1 | 1 | ? | 0 | 1 | ? | 1 | 1 | 0 | ? | 1 | 1 | 1 |
| *Latimeria* | 1 | 0 | 1 | 0 | 0 | 0 | 1 | 0 | 1 | 1 | 0 | 0 | 1 | 0 | 1 | 1 | 0 | 0 | 1 | 1 | 1 |
| *Miguashaia* | 0 | 0 | ? | ? | ? | ? | ? | ? | ? | ? | ? | ? | ? | ? | ? | ? | ? | ? | ? | ? | ? |
| *Axelrodichthys* | 0 | 1 | 1 | 0 | 0 | 0 | 1 | 1 | 1 | 1 | ? | 0 | ? | ? | 1 | 1 | 0 | ? | 1 | 1 | 1 |
| *Holopterygius* | ? | ? | 0 | 0 | ? | ? | ? | ? | ? | ? | ? | ? | ? | 0 | 0 | ? | ? | ? | ? | ? | ? |
| *Garnbergia* | ? | ? | ? | ? | ? | ? | ? | ? | ? | ? | ? | ? | ? | ? | ? | ? | ? | ? | ? | ? | ? |
| *Parnaibaia* | 0 | 0 | ? | ? | ? | ? | ? | ? | ? | ? | ? | ? | ? | ? | ? | ? | ? | ? | ? | ? | ? |
| *Swenzia* | ? | 0 | ? | ? | ? | ? | ? | ? | ? | ? | ? | ? | 1 | ? | ? | ? | ? | ? | ? | ? | ? |
| *Guizhoucoelacanthus* | 0 | ? | ? | ? | ? | ? | ? | ? | ? | ? | ? | ? | ? | ? | ? | ? | ? | ? | ? | ? | ? |
| *Piveteauia* | ? | ? | ? | ? | ? | ? | ? | ? | ? | ? | ? | ? | ? | ? | ? | ? | ? | ? | ? | ? | ? |
| *Euporosteus* | ? | ? | 0 | 1 | 1 | ? | 0 | ? | ? | ? | ? | 1 | 0 | 1 | 0 | ? | 1 | ? | 0 | ? | 0 |
| *Axelia* | 0 | 1 | ? | ? | ? | ? | ? | ? | ? | ? | ? | ? | ? | ? | ? | ? | ? | ? | ? | ? | ? |
| *Wimania* | ? | ? | ? | ? | ? | ? | ? | ? | ? | ? | ? | ? | ? | ? | ? | ? | ? | ? | ? | ? | ? |
| *Ticinepomis* | ? | ? | ? | ? | ? | ? | ? | ? | ? | ? | ? | ? | ? | ? | ? | ? | ? | ? | ? | ? | ? |
| *Rebellatrix* | ? | ? | ? | ? | ? | ? | ? | ? | ? | ? | ? | ? | ? | ? | ? | ? | ? | ? | ? | ? | ? |
| *Megalocoelacanthus* | 0 | 1 | 1 | 1 | 0 | 0 | 1 | 1 | 1 | ? | ? | 0 | 1 | ? | ? | ? | 0 | ? | 1 | ? | 1 |

|  | **88** | **89** | **90** | **91** | **92** | **93** | **94** | **95** | **96** | **97** | **98** | **99** | **100** | **101** | **102** | **103** | **104** | **105** | **106** |
| --- | --- | --- | --- | --- | --- | --- | --- | --- | --- | --- | --- | --- | --- | --- | --- | --- | --- | --- | --- |
| Actinopterygians | 0 | ? | 1 | 0 | 0 | 0 | 0 | 0 | 0 | 0 | 0 | 0 | 0 | 0 | 0 | 0 | 0 | 0 | 0 |
| Porolepiforms | 0 | 0 | 0 | 0 | 0 | 0 | 0 | 0 | 0 | ? | 0 | 0 | 0 | 0 | 0 | 0 | 0 | 0 | 0 |
| *Diplocercides* | ? | ? | 0 | 0 | 0 | 1 | 0 | 1 | 0 | 0 | 0 | 0 | 0 | ? | ? | 0 | 0 | 0 | 0 |
| *Rhabdoderma* | 1 | 0 | 1 | 0 | 0 | 1 | 1 | 1 | 0 | 0 | 0 | 0 | 0 | 1 | 1 | 0 | 0 | 0 | 0 |
| *Caridosuctor* | 1 | 0 | 1 | ? | 0 | 1 | 1 | 1 | 0 | 1 | 0 | 0 | 0 | 1 | 1 | 0 | 0 | ? | 0 |
| *Hadronector* | 1 | 0 | 1 | ? | 0 | 1 | 1 | 1 | 0 | 0 | 0 | 0 | 0 | 0 | 1 | 0 | ? | ? | 0 |
| *Polyosteorhynchus* | 1 | ? | 1 | 1 | 0 | 1 | 1 | 1 | ? | 1 | 0 | 0 | 0 | 1 | 0 | 0 | 0 | ? | 0 |
| *Allenypterus* | 1 | 0 | 1 | 0 | 0 | 1 | 0 | 1 | 0 | 1 | 0 | 0 | 0 | 0 | 0 | 0 | 0 | 0 | 0 |
| *Lochmocercus* | 1 | 0 | 1 | ? | ? | 1 | 0 | 1 | 0 | 0 | 0 | 0 | 0 | 1 | 0 | 0 | ? | ? | 0 |
| *Coelacanthus* | 1 | 0 | 1 | 0 | 0 | 1 | 1 | 1 | 0 | 1 | 0 | 0 | 0 | 0 | 1 | 0 | 0 | ? | 0 |
| *Spermatodus* | ? | ? | ? | ? | ? | ? | ? | ? | ? | ? | ? | ? | ? | ? | ? | ? | 0 | ? | 0 |
| *Whiteia* | 1 | ? | 1 | 0 | 0 | 1 | 1 | 1 | 2 | 0 | 1 | 0 | 0 | 0 | 1 | 0 | 0 | 1 | 0 |
| *Laugia* | 1 | 0 | 1 | 1 | 0 | 1 | 1 | 1 | 1 | 1 | 0 | 1 | 1 | 0 | 1 | 0 | 0 | ? | 0 |
| *Sassenia* | 1 | ? | ? | ? | ? | ? | ? | ? | ? | ? | ? | ? | ? | ? | ? | ? | 0 | ? | 0 |
| *Chinlea* | 1 | ? | 1 | ? | 1 | 1 | 1 | 1 | 1 | ? | 0 | 0 | 0 | ? | 1 | 0 | 1 | ? | 1 |
| *Diplurus* | 1 | 0 | 1 | 1 | 1 | 1 | 1 | 1 | 1 | 0 | 1 | 0 | 0 | 0 | 1 | 0 | 1 | ? | 0 |
| *Holophagus* | 1 | ? | 1 | 1 | 0 | 1 | 1 | 1 | 1 | ? | 1 | 1 | 0 | 0 | 1 | 1 | 0 | ? | 0 |
| *Undina* | 1 | 0 | 1 | ? | 0 | 1 | 1 | 1 | 1 | 0 | 1 | 0 | 0 | 0 | 1 | 0 | 0 | 1 | 0 |
| *Coccoderma* | 1 | 1 | 1 | 1 | 0 | 1 | 1 | 1 | 0 | 0 | 0 | 1 | 1 | 0 | 1 | 0 | 0 | 1 | 1 |
| *Libys* | 1 | 1 | 1 | 1 | 0 | 1 | 1 | 1 | 1 | 0 | 1 | 1 | 0 | 0 | 1 | 1 | 0 | 1 | 0 |
| *Mawsonia* | ? | ? | 1 | ? | ? | 1 | 1 | 1 | ? | 0 | 1 | 0 | 0 | 0 | 1 | 0 | 1 | ? | 1 |
| *Macropoma* | 1 | 1 | 1 | 1 | 0 | 1 | 1 | 1 | 2 | 0 | 1 | 0 | 0 | 0 | 1 | 0 | 1 | 1 | 0 |
| *Latimeria* | 1 | 0 | 1 | 1 | 0 | 1 | 1 | 1 | 2 | 0 | 1 | 0 | 0 | 0 | 1 | 0 | 0 | 1 | 0 |
| *Miguashaia* | 1 | ? | 0 | ? | ? | 0 | 0 | 0 | 0 | ? | 0 | 0 | 0 | ? | ? | 0 | 0 | 0 | 0 |
| *Axelrodichthys* | 1 | ? | 1 | 1 | 1 | 1 | 1 | 1 | 1 | 0 | 1 | 0 | 0 | 0 | 1 | 0 | 1 | ? | 1 |
| *Holopterygius* | ? | ? | 1 | 0 | ? | 0 | 0 | 1 | 0 | 1 | ? | 1 | ? | ? | ? | ? | 0 | 0 | ? |
| *Garnbergia* | ? | ? | ? | ? | ? | ? | ? | ? | 1 | ? | 0 | ? | ? | 0 | 1 | 0 | 0 | ? | 0 |
| *Parnaibaia* | 1 | ? | 1 | ? | 1 | 1 | 1 | 1 | 1 | 0 | 1 | 0 | 0 | 0 | ? | 0 | 1 | ? | 0 |
| *Swenzia* | ? | ? | 1 | ? | 0 | 1 | 1 | ? | ? | 0 | ? | ? | 0 | 0 | 1 | ? | 0 | ? | 0 |
| *Guizhoucoelacanthus* | 1 | ? | 1 | 0 | 0 | 1 | 1 | 0 | 2 | 0 | 0 | 0 | 0 | 0 | 1 | 0 | 0 | 0 | 1 |
| *Piveteauia* | 1 | ? | ? | ? | ? | 1 | ? | 1 | 2 | 0 | 0 | 0 | 1 | 0 | 1 | 0 | ? | ? | 0 |
| *Euporosteus* | ? | ? | ? | ? | ? | ? | ? | ? | ? | ? | ? | ? | ? | ? | ? | ? | ? | ? | ? |
| *Axelia* | ? | ? | ? | ? | ? | ? | ? | ? | ? | ? | ? | 0 | ? | ? | ? | ? | 0 | ? | 0 |
| *Wimania* | ? | ? | ? | ? | 0 | ? | ? | ? | ? | ? | ? | ? | ? | ? | ? | ? | 0 | ? | 0 |
| *Ticinepomis* | 1 | ? | 1 | ? | 0 | 1 | 1 | 1 | 1 | 0 | 1 | 0 | 0 | 0 | 0 | 1 | 0 | 0 | ? |
| *Rebellatrix* | 1 | 0 | 1 | 1 | 0 | 1 | 1 | 1 | 0 | 0 | 0 | 0 | 0 | 0 | 1 | 0 | 0 | 0 | 0 |
| *Megalocoelacanthus* | 1 | ? | ? | 1 | ? | ? | ? | ? | ? | ? | ? | ? | ? | ? | ? | ? | ? | ? | ? |

|  | **107** | **108** | **109** | **110** |
| --- | --- | --- | --- | --- |
| Actinopterygians | 0 | 0 | 0 | 0 |
| Porolepiforms | 0 | 0 | 0 | 0 |
| *Diplocercides* | ? | ? | ? | 0 |
| *Rhabdoderma* | 1 | 0 | 0 | 0 |
| *Caridosuctor* | 1 | 0 | 0 | ? |
| *Hadronector* | 1 | 0 | 0 | 0 |
| *Polyosteorhynchus* | 1 | 0 | 0 | 0 |
| *Allenypterus* | 1 | 0 | 1 | 0 |
| *Lochmocercus* | ? | 0 | 0 | 0 |
| *Coelacanthus* | 1 | 0 | 0 | 0 |
| *Spermatodus* | ? | ? | ? | ? |
| *Whiteia* | 0 | 0 | 0 | 0 |
| *Laugia* | 1 | 1 | 0 | 0 |
| *Sassenia* | ? | ? | 0 | 0 |
| *Chinlea* | ? | 0 | 0 | ? |
| *Diplurus* | 0 | 0 | 0 | 0 |
| *Holophagus* | 1 | 0 | 0 | ? |
| *Undina* | 1 | 0 | 0 | 1 |
| *Coccoderma* | 1 | 1 | 0 | 0 |
| *Libys* | 1 | 0 | 0 | 1 |
| *Mawsonia* | 1 | 0 | ? | 0 |
| *Macropoma* | 1 | 0 | 0 | 1 |
| *Latimeria* | 0 | 0 | 0 | 1 |
| *Miguashaia* | ? | ? | 0 | ? |
| *Axelrodichthys* | 1 | 0 | 0 | 0 |
| *Holopterygius* | ? | ? | 1 | ? |
| *Garnbergia* | ? | ? | 0 | ? |
| *Parnaibaia* | 1 | 0 | ? | ? |
| *Swenzia* | 1 | ? | ? | ? |
| *Guizhoucoelacanthus* | ? | ? | 0 | ? |
| *Piveteauia* | 1 | ? | 0 | ? |
| *Euporosteus* | ? | ? | ? | ? |
| *Axelia* | ? | ? | ? | 0 |
| *Wimania* | ? | ? | ? | 0 |
| *Ticinepomis* | 0 | ? | 0 | ? |
| *Rebellatrix* | ? | 0 | ? | ? |
| *Megalocoelacanthus* | ? | ? | ? | 1 |
