# Supplementary material for: The Giant Cretaceous Coelacanth (Actinistia, Sarcopterygii) Megalocoelacanthus dobiei Schwimmer, Stewart & Williams, 1994, and Its Bearing on Latimerioidei Interrelationships
Source: PLoS One. 2012 Nov 27;7(11):e49911. doi: 10.1371/journal.pone.0049911 (PMC3507921; doi:10.1371/journal.pone.0049911)
Supplement: Information S5 — Diagnostic information for the node and terminal taxa of the strict consensus tree illustrated Figure 22 . (DOC) [file pone.0049911.s005.doc]

# Supporting information 5: Diagnostic information for the nodes and terminal taxa of the strict consensus tree illustrated Fig. 22

Node number correspond to the strict consensus tree illustrated Fig. 20. Characters and character states are given in Appendix 1. Only unambiguous synapomorphies are listed. Asterisks indicate non-homoplastic synapomorphies. CI: Consistency Index.

| Node | Character | Change | CI |
| --- | --- | --- | --- |
| 1 | 33 | 1🡪0 | 0.500 |
|  | 88 | 0🡪1* | 1.000 |
| 2 | 7 | 1🡪2 | 0.333 |
|  | 19 | 0🡪1* | 1.000 |
|  | 93 | 0🡪1 | 0.500 |
|  | 95 | 0🡪1 | 0.500 |
| 3 | 23 | 2🡪0 | 0.333 |
|  | 24 | 1🡪0* | 1.000 |
|  | 34 | 1🡪0 | 0.333 |
|  | 45 | 0🡪1 | 0.250 |
|  | 90 | 0🡪1 | 0.500 |
| 4 | 54 | 0🡪1* | 1.000 |
|  | 63 | 0🡪1 | 0.333 |
|  | 97 | 0🡪1 | 0.250 |
| 5 | 61 | 0🡪1* | 1.000 |
|  | 94 | 0🡪1 | 0.500 |
|  | 101 | 0🡪1 | 0.333 |
| 6 | 1 | 0🡪1 | 0.250 |
|  | 20 | 0🡪1* | 1.000 |
|  | 56 | 0🡪1 | 0.200 |
|  | 59 | 1🡪0 | 0.167 |
|  | 102 | 0🡪1 | 0.333 |
| 7 | 64 | 0🡪1 | 0.333 |
|  | 97 | 1🡪0 | 0.250 |
| 8 | 23 | 0🡪2 | 0.333 |
|  | 29 | 0🡪1 | 0.333 |
|  | 62 | 0🡪1* | 1.000 |
|  | 101 | 1🡪0 | 0.333 |
| 9 | 1 | 1🡪0 | 0.250 |
|  | 18 | 0🡪1 | 0.500 |
|  | 21 | 0🡪1 | 0.333 |
|  | 70 | 1🡪0 | 0.250 |
|  | 71 | 1🡪0 | 0.500 |
|  | 78 | 1🡪0 | 0.500 |
|  | 82 | 0🡪1* | 1.000 |
|  | 86 | 0🡪1* | 1.000 |
| 10 | 5 | 0🡪1 | 0.250 |
|  | 10 | 1🡪0 | 0.500 |
|  | 26 | 0🡪1 | 0.250 |
|  | 27 | 0🡪1 | 0.286 |
|  | 47 | 0🡪1 | 0.333 |
| 11 | 53 | 0🡪1 | 0.500 |
|  | 91 | 0🡪1 | 0.333 |
| 12 | 96 | 0🡪1 | 0.500 |
| 13 | 98 | 0🡪1 | 0.333 |
| 14 | 103 | 0🡪1 | 0.500 |
| 15 | 57 | 0🡪1 | 0.333 |
|  | 105 | 0🡪1 | 0.333 |
| 16 | 23 | 2🡪3 | 0.333 |
|  | 50 | 0🡪2 | 0.500 |
|  | 68 | 0🡪1 | 0.250 |
| 17 | 9 | 0🡪1 | 0.500 |
|  | 27 | 1🡪0 | 0.286 |
|  | 32 | 0🡪1 | 0.200 |
|  | 67 | 0🡪1 | 0.143 |
| 18 | 59 | 0🡪1 | 0.167 |
|  | 103 | 1🡪0 | 0.500 |
| 19 | 39 | 0🡪1 | 0.500 |
|  | 40 | 0🡪1* | 1.000 |
|  | 96 | 1🡪2 | 0.500 |
| 20 | 26 | 1🡪0 | 0.250 |
|  | 30 | 0🡪1 | 0.200 |
| 21 | 14 | 1🡪0 | 0.500 |
|  | 56 | 1🡪0 | 0.200 |
|  | 92 | 0🡪1* | 1.000 |
|  | 104 | 0🡪1 | 0.500 |
| 22 | 43 | 0🡪1* | 1.000 |
|  | 57 | 0🡪1 | 0.333 |
| 23 | 27 | 1🡪2 | 0.286 |
|  | 49 | 1🡪2 | 0.400 |
|  | 106 | 0🡪1 | 0.333 |
| 24 | 1 | 0🡪1 | 0.250 |
|  | 16 | 0🡪1* | 1.000 |
|  | 30 | 1🡪0 | 0.200 |
|  | 38 | 0🡪1 | 0.500 |
|  | 41 | 0🡪1* | 1.000 |
|  | 66 | 0🡪1* | 1.000 |
| 25 | 46 | 0🡪1 | 0.250 |
|  | 96 | 0🡪2 | 0.500 |
| 26 | 36 | 0🡪1 | 0.250 |
| 27 | 7 | 2🡪1 | 0.333 |
| 28 | 30 | 1🡪0 | 0.200 |
|  | 32 | 1🡪0 | 0.200 |
|  | 45 | 1🡪0 | 0.250 |
|  | 47 | 0🡪1 | 0.333 |
|  | 48 | 0🡪1 | 0.333 |
|  | 91 | 0🡪1 | 0.333 |
|  | 99 | 0🡪1 | 0.250 |
|  | 100 | 0🡪1 | 0.500 |
|  | 108 | 0🡪1* | 1.000 |
| 29 | 25 | 1🡪0 | 0.250 |
|  | 109 | 0🡪1* | 1.000 |
| 30 | 83 | 0🡪1* | 1.000 |
| *Miguashaia* | Ambiguous autapomorphies | | |
| *Euporosteus* | 12 | 0🡪2 | 0.667 |
|  | 15 | 0🡪1 | 0.167 |
|  | 32 | 1🡪0 | 0.200 |
| *Lochmocercus* | 21 | 0🡪1 | 0.333 |
|  | 101 | 0🡪1 | 0.333 |
| *Hadronector* | 23 | 0🡪1 | 0.333 |
|  | 94 | 0🡪1 | 0.500 |
|  | 102 | 0🡪1 | 0.333 |
| *Holopterygius* | 59 | 1🡪0 | 0.167 |
|  | 93 | 1🡪0 | 0.500 |
|  | 99 | 0🡪1 | 0.250 |
| *Polyosteorhynchus* | 21 | 0🡪1 | 0.333 |
|  | 23 | 0🡪1 | 0.333 |
|  | 91 | 0🡪1 | 0.333 |
| *Rhabdoderma* | 8 | 1🡪0 | 0.200 |
|  | 46 | 0🡪1 | 0.250 |
|  | 63 | 1🡪0 | 0.333 |
|  | 77 | 1🡪0 | 0.500 |
|  | 78 | 1🡪0 | 0.500 |
| *Sassenia* | 34 | 0🡪1 | 0.333 |
|  | 69 | 1🡪0 | 0.500 |
|  | 80 | 0🡪1 | 0.333 |
|  | 84 | 0🡪1 | 0.333 |
| *Coccoderma* | 27 | 0🡪1 | 0.286 |
|  | 37 | 0🡪1 | 0.333 |
|  | 38 | 0🡪1 | 0.500 |
|  | 49 | 1🡪0 | 0.400 |
|  | 89 | 0🡪1 | 0.333 |
|  | 106 | 0🡪1 | 0.333 |
| *Laugia* | 2 | 0🡪1 | 0.200 |
|  | 7 | 2🡪1 | 0.333 |
|  | 23 | 2🡪0 | 0.333 |
|  | 26 | 0🡪1 | 0.250 |
|  | 31 | 1🡪0 | 0.500 |
|  | 56 | 1🡪0 | 0.200 |
|  | 96 | 0🡪1 | 0.500 |
|  | 97 | 0🡪1 | 0.250 |
| *Spermatodus* | 15 | 0🡪1 | 0.167 |
|  | 18 | 0🡪1 | 0.500 |
|  | 26 | 0🡪1 | 0.250 |
|  | 34 | 0🡪1 | 0.333 |
|  | 68 | 0🡪1 | 0.250 |
|  | 70 | 1🡪0 | 0.250 |
| *Piveteauia* | 13 | 0🡪1 | 0.500 |
|  | 29 | 1🡪0 | 0.333 |
|  | 100 | 0🡪1 | 0.500 |
| *Wimania* | 31 | 1🡪0 | 0.500 |
| *Axelia* | Ambiguous autapomorphies | | |
| *Guizhoucoelacanthus* | 15 | 1🡪0 | 0.167 |
|  | 27 | 0🡪1 | 0.286 |
|  | 52 | 1🡪0 | 0.500 |
|  | 106 | 0🡪1 | 0.333 |
| *Whiteia* | 8 | 1🡪0 | 0.200 |
|  | 57 | 0🡪1 | 0.333 |
|  | 59 | 0🡪1 | 0.167 |
|  | 98 | 0🡪1 | 0.333 |
|  | 105 | 0🡪1 | 0.333 |
| *Coelacanthus* | 4 | 0🡪1 | 0.333 |
|  | 37 | 0🡪1 | 0.333 |
|  | 42 | 0🡪1 | 0.250 |
|  | 97 | 0🡪1 | 0.250 |
| *Rebellatrix* | 59 | 0🡪1 | 0.167 |
| *Garnbergia* | 36 | 0🡪1 | 0.250 |
| *Diplurus* | 9 | 0🡪1 | 0.500 |
|  | 23 | 2🡪0 | 0.333 |
|  | 42 | 0🡪1 | 0.250 |
|  | 44 | 1🡪0 | 0.333 |
|  | 46 | 0🡪1 | 0.250 |
|  | 49 | 1🡪0 | 0.400 |
|  | 50 | 0🡪1 | 0.250 |
|  | 107 | 1🡪0 | 0.200 |
| *Parnaibaia* | 2 | 0🡪1 | 0.200 |
|  | 3 | 0🡪1 | 0.250 |
|  | 4 | 0🡪1 | 0.333 |
| *Axelrodichthys* | 37 | 0🡪1 | 0.333 |
| *Mawsonia* | 51 | 1🡪0 | 0.500 |
| *Chinlea* | 56 | 0🡪1 | 0.200 |
|  | 67 | 0🡪1 | 0.143 |
|  | 98 | 1🡪0 | 0.333 |
| *Ticinepomis* | 42 | 0🡪1 | 0.250 |
|  | 102 | 1🡪0 | 0.333 |
|  | 107 | 1🡪0 | 0.200 |
| *Latimeria* | 4 | 0🡪1 | 0.333 |
|  | 46 | 0🡪1 | 0.250 |
|  | 50 | 0🡪1 | 0.500 |
|  | 107 | 1🡪0 | 0.200 |
| *Macropoma* | 67 | 1🡪0 | 0.143 |
|  | 89 | 0🡪1 | 0.333 |
|  | 104 | 0🡪1 | 0.500 |
| *Undina* | 5 | 1🡪0 | 0.250 |
|  | 63 | 1🡪0 | 0.333 |
| *Holophagus* | 1 | 0🡪1 | 0.250 |
| *Megalocoelacanthus* | 5 | 1🡪0 | 0.250 |
|  | 56 | 1🡪0 | 0.200 |
